# A systematic review of cost-utility analyses of screening methods in latent tuberculosis infection in high-risk populations

**DOI:** 10.1186/s12890-022-02149-x

**Published:** 2022-10-05

**Authors:** James Mahon, Sophie Beale, Hayden Holmes, Mick Arber, Vladyslav Nikolayevskyy, Riccardo Alagna, Davide Manissero, David Dowdy, Giovanni Battista Migliori, Giovanni Sotgiu, Raquel Duarte

**Affiliations:** 1 York Health Economics Consortium, University of York, York, UK; 2 QIAGEN, Manchester, UK; 3 QIAGEN SRL, Via Filippo Sassetti 16, 20124 Milan, Italy; 4 Johns Hopkins Bloomberg School of Public Health, Baltimore, MD USA; 5 Servizio di Epidemiologia Clinica delle Malattie Respiratorie, Istituti Clinici Scientifici Maugeri IRCCS, Tradate, Italy; 6 Scinze Mediche Chirurgiche E Sperimentali, Universita’ degli Studi di Sassari, Sassari, Italy; 7 EPI Unit, Instituto de Saúde Pública da Universidade do Porto, Porto, Portugal; 8 Unidade de Investigação Clínica da Administração Regional de Saúde do Norte, Porto, Portugal; 9 Departamento de Ciências de Saúde Pública, Ciências Forenses e Educação Médica, Universidade do Porto, Porto, Portugal; 10 Serviço de Pneumologia, Centro Hospitalar de Vila Nova de Gaia/Espinho, Vila Nova de Gaia, Portugal

**Keywords:** Tuberculosis, Systematic Review, Cost-effectiveness, Cost-utility, Testing

## Abstract

**Background:**

The World Health Organisation (WHO) recommends that testing and treatment for latent tuberculosis infection (LTBI) should be undertaken in high-risk groups using either interferon gamma release assays (IGRAs) or a tuberculin skin test (TST). As IGRAs are more expensive than TST, an assessment of the cost-effectiveness of IGRAs can guide decision makers on the most appropriate choice of test for different high-risk populations. This current review aimed to provide the most up to date evidence on the cost-effectiveness evidence on LTBI testing in high-risk groups—specifically evidence reporting the costs per QALY of different testing strategies.

**Methods:**

A comprehensive search of databases including MEDLINE, EMBASE and NHS-EED was undertaken from 2011 up to March 2021. Studies were screened and extracted by two independent reviewers. The study quality was assessed using the Bias in Economic Evaluation Checklist (ECOBIAS). A narrative synthesis of the included studies was undertaken.

**Results:**

Thirty-two studies reported in thirty-three documents were included in this review. Quality of included studies was generally high, although there was a weakness across all studies referencing sources correctly and/or justifying choices of parameter values chosen or assumptions where parameter values were not available. Inclusions of IGRAs in testing strategies was consistently found across studies to be cost-effective but this result was sensitive to underlying LTBI prevalence rates.

**Conclusion:**

While some concerns remain about uncertainty in parameter values used across included studies, the evidence base since 2010 has grown with modelling approaches addressing the weakness pointed out in previous reviews but still reaching the same conclusion that IGRAs are likely to be cost-effective in high-income countries for high-risk populations. Evidence is also required on the cost-effectiveness of different strategies in low to middle income countries and countries with high TB burden.

**Supplementary Information:**

The online version contains supplementary material available at 10.1186/s12890-022-02149-x.

## Introduction

In 2020, the World Health Organisation (WHO) Consolidated Guideline on Tuberculosis [1] and in 2022 the Clinical Standards for TB Infection recommend that testing for and treatment of latent tuberculosis infection (LTBI) should be undertaken in high-risk groups (household contact > 5 years old and other risks groups including silicosis, dialysis, anti-TNF agent treatment, preparation for transplantation or other risks in national guidelines) for prevention of active TB. This intervention has been included under Pillar 1 of the End TB strategy and is considered a core activity to pursue for TB Elimination [2]. The Guideline and the Standards recommend that testing should be with an interferon-gamma release assay (IGRA) or with a tuberculin skin test (TST) but considered that whilst TST likely required fewer resources than IGRA there was insufficient evidence to suggest that one test was better than the other at predicting progression to active TB [1]. Given the differences in resource use for TST and IGRA, a review of the cost-effectiveness evidence on the use of TST or IGRA in high-risk groups could aid decision makers on the appropriate choice of the most suitable test to utilise to diagnose LTBI infection.

Evidence from studies published before 2010 all consistently point to IGRA being cost-effective, relative to TST in high-risk populations in high income countries [3]. Since 2010, there have been several systematic reviews that have assessed the cost-effectiveness of different testing strategies for LTBI [4–6]. These reviews have highlighted that studies have generally been of moderate-to-high quality [6] in mostly middle to high income settings and that LTBI screening is most cost-effective if screening is restricted to high-risk groups such as migrants from high-burden countries [5]

However, reviews have also identified the following weaknesses in studies:

- The accuracy of tests in predicting future reactivation to active TB is unknown and not included in models [4]
- Model structures fail to account for onward transmission [4]
- The lack of consistent methods between studies makes it difficult to draw firm conclusions [6]

This current review aimed to provide the most up to date evidence on the cost-effectiveness evidence on LTBI testing in high-risk groups—specifically evidence reporting the costs per QALY of different testing strategies to account for some of the inconsistency in methods between studies.

## Methods

The systematic review was registered with the NIHR PROSPERO international prospective database of systematic reviews (ID: CRD42021240148).

### Eligibility criteria

The target population of the review was people at high-risk of having LTBI or progressing from LTBI to active TB [1]. Populations of specific interest were: migrants, contacts of people with active TB, children, healthcare workers, immunocompromised and people with HIV. The following LTBI tests were considered eligible: IGRA (QuantiFERON TB Gold GIT or Plus (QIAGEN, Hilden. Germany) or T-SPOT (Oxford Immunotec, Abingdon,UK) if presented separately), TST, chest X-ray (CXR). Only cost-utility studies were eligible for inclusion i.e. presented results as incremental cost-effectiveness ratios (ICERS) per Quality Adjusted Life Year (QALY) gained. Studies published only as conference abstracts were included in the review if adequate information was provided to allow appropriate study assessment. Editorials, letters, news articles and comments were not eligible for inclusion in the review.

### Search strategy

A MEDLINE (OvidSP) search strategy was designed to identify eligible studies. The final MEDLINE strategy and resources searched (using translations of the strategy where required) are presented in Additional file 1: Appendix A. The strategy was restricted by date to studies published from 2011 to March 2021. In addition to searching the HTA database, targeted searches of the technology assessment and regulatory agency websites listed above (NICE, CADTH and ICER) were conducted.

Recent research published as conference abstracts was identified by searching Embase (which indexes a significant number of conference publications). We also checked the reference lists of any included studies and relevant systematic reviews published in the last 5 years for any eligible studies that might have been missed by the database searches.

### Study selection

A single reviewer (MA) assessed the search results according to their relevance in providing information on economics evaluations and removed the obviously irrelevant records. Two reviewers (JM, SB) independently assessed the titles and abstracts of remaining records for relevance against the eligibility criteria. Two reviewers (JM, SB) independently assessed the full texts for relevance against the eligibility criteria. A third reviewer (HH) adjudicated any disagreements at both abstract and full text assessment stages.

The number of records included and removed at each selection stage were recorded with studies excluded after assessment of the full document also recorded with the reasons for exclusion.

Where results for one study are reported in more than one paper, all related papers were identified and grouped together to ensure that results from studies were only included once.

### Data extraction

One reviewer extracted data from the eligible studies (SB) and a second reviewer checked all the data points (JM). A data extraction sheet was developed in Excel (Microsoft, Redmond, USA) and piloted on a number of studies before progressing to full data extraction.

Information was extracted on study: details (title, author, year of study); overview (objective, conclusions, limitations (self-reported); characteristics (study design, number of participating centres and countries, setting, population description [including high-risk groups)], subgroups analysed, eligibility criteria, number of patients analysed); interventions (testing strategies considered, sensitivity, specificity, negative predictive value (NPV) and positive predictive value (PPV) of testing strategies); model (country perspective, type, time horizon, cycle, health states, assumptions); data sources; cost currency and year; outcomes (QALYs, total costs, ICERs); and sensitivity analyses (methods and results).

### Quality assessment

One reviewer (SB) assessed the risk of bias in each included study using the ECOBIAS tool [7]. A second reviewer checked the risk of bias assessment (JM).

### Data synthesis

The extracted data from studies were summarised in tables focusing on methods and results. The studies were synthesised narratively for each risk group separately, with an overall discussion of the methodological approaches taken and the consistency and robustness of the findings across all studies on the cost-effectiveness of different testing strategies across high-risk groups.

## Results

**Figure 1:**
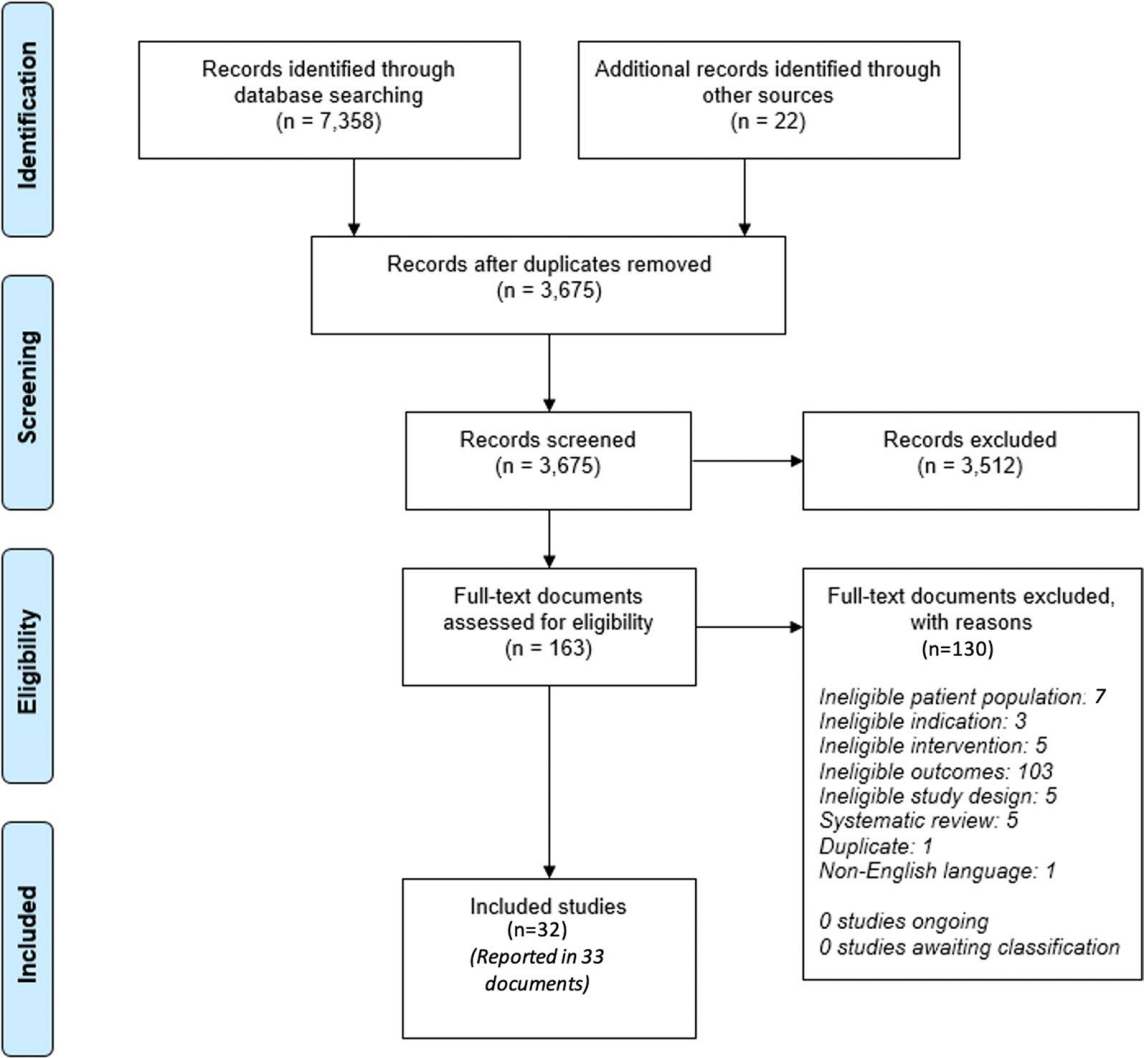
Study selection PRISMA diagram

The database searches, website searches and conference hand-searches were conducted between 09/12/2020 and 22/12/2020. The searches identified 7,380 records. Following deduplication, 3,675 records were assessed for relevance. There were 3,512 records excluded based upon the abstract alone with 163 full text documents assessed for eligibility for inclusion. On full text review, there were 32 studies in 33 [8–40] publications that met the inclusion criteria and were extracted and quality assessed. There were no disagreements between reviewers at any stage and so no third reviewer was required. A full PRISMA diagram including reasons for exclusion at the full text stage is provided in Fig. 1.

A summary of the populations covered by studies is shown in Table 1, noting that studies could report findings for more than one population. All included studies were in high income countries. The characteristics of included studies are summarised in Table 2.

**Table 1:** Included studies by population of interest

Population	Number of studies
Migrants	13 (14 publications)
HIV	10
Contacts	5
Immunocompromised	7
Healthcare workers	5
Other high-risk populations	3
Children	2

**Table 2:** Included studies

Author/trial ID	Country/region	Description of population	Tests assessed
Abubakar et al. [17]	UK	Migrants, Contacts	1. QFT with 4R 2. QFT with 6H 3. QFT with 3HP 4. TST with 4R 5. TST with 6H 6. TST with 3HP 7. CXR
Al Abri et al. [20]	Oman	Migrants	TST QFT-GIT T-SPOT.TB CXR
Auguste et al. [16]	UK	Migrants, Immunocompromised, Children	TST/INH TST/RIF IGRA/INH IGRA/RIF SEQ/INH SEQ/RIF (SEQ = BC + TST at time of medical examination) No intervention
Campbell et al. [13]	Canada	Migrants	Base case (no screening or treatment for LTBI) IGRA/INH TST/INH
Campbell et al. [14]	Canada	Migrants	Base case (BC): CXR + medical history + symptom screen BC + TST/INH BC + TST/RIF BC + IGRA/INH BC + IGRA/RIF SEQ/INH (SEQ = BC + TST at time of medical examination) SEQ/RIF
Campbell et al.a [15]	Canada (British Columbia)	Migrants	QFT, TST
Capocci et al. [22]	UK	HIV+	NICE Guideline (not in abstract but taken from guideline) IGRA + TST only if CD4 < 500 BHIVA Guideline (not in abstract but taken from guideline) IGRA test only if from sSA: ART < 2 years from MI: CD4 < 400 and ART < 2 years from low TB incidence country:CD4 < 350 and ART < 6 months
Capocci et al. [23]	UK	HIV+	NICE Guideline (not in abstract but taken from guideline) IGRA + TST only if CD4 < 500 BHIVA Guideline (not in abstract but taken from guideline) IGRA test only if from sSA: ART < 2 years from MI: CD4 < 400 and ART < 2 years from low TB incidence country:CD4 < 350 and ART < 6 months
Capocci et al. [24]	UK	HIV+	Testing all patients IGRA for all NICE Guideline IGRA + TST only if CD4 < 500 BHIVA Guideline IGRA test only if from sSA: ART < 2 years from MI: CD4 < 400 and ART < 2 years from low TB incidence country:CD4 < 350 and ART < 6 months
Capocci et al. [25]	UK	HIV+	TST, IGRA
Capocci et al. [26]	UK	HIV+	TST, IGRA, CXR
Capocci et al. [27]	UK	HIV+	TST, IGRA, CXR
Eralp et al. [35]	UK	Healthcare workers	TST IGRA TST + IGRA (X-ray used to confirm +ve test result)
Goodell et al. [12]	USA	Migrants	QFT, TSPOT, TST, CXR
Hayama et al. [30]	UK	Contacts	TST alone, IGRA (QFT-GIT or T-SPOT) alone, TST +ve followed by IGRA, TST −ve followed by IGRA
Jo et al. [11]	USA	Migrants	TST, IGRA
Kowada [18]	Japan	Migrants	TST QFT T-SPOT TST/QFT TST/T-SPOT CXR
Kowada [19]	Japan	Migrants, HIV+, contacts	TST QFT T-SPOT TST/QFT TST/T-SPOT CXR
Kowada [21]	Japan	Healthcare workers	For LTBI: TST, QFT For active TB: CXR
Kowada [28]	Japan	HIV+	QFT, TST, TST/QFT, CXR
Kowada [29]	Japan	Other (care home residents)	TST QFT T-SPOT CXR
Kowada [31]	Japan	Contacts	IGRA (QFT-GIT or T-SPOT), TST
Kowada [32]	Japan	Immunocompromised	QFR TSPOT TST CXR
Kowada [36]	Japan (assumed)	Healthcare workers	Annual QFT alone vs an initial QFT followed by annual CXR
Kowada [38]	Japan (assumed)	Other (Mental health patients who are smokers)	QFT T-SPOT TST TST followed by QFT TST followed by T-SPOT CXR No screening
Kowada [40]	Japan	Children	QFT TST CXR
Laskin et al. [33]	USA	Immunocompromised	TST IGRA Questionnaire
Li et al. [39]	Hong Kong	Other (Older people on entry to residential care)	QFT followed by confirmatory CXR and then smear test
Linas et al. [8, 9]	USA	Migrants, contacts, immunocompromised	1. QFT with 4R 2. QFT with 6H 3. QFT with 3HP 4. TST with 4R 5. TST with 6H 6. TST with 3HP 7. CXR
Png et al. [37]	Tertiary care hospital in Singapore	Healthcare workers	QFT
Tasillo et al. [10]	USA	Migrants, HIV+	IGRA
Van der Have et al. [34]	Europe	Immuoncompromised	TST with CXR TST with CXR followed by QFT

### Summary of modelling approaches

#### Model structure

In all studies, a decision tree was employed to determine LTBI test and treatment outcomes. Six studies subsequently employed a discrete event simulation model to estimate long term TB development [11, 16–19, 40], while the remainder used a Markov model (or model of unspecified type) for long term outcomes. The conclusions of studies by modelling approach did not differ substantially. The perspective in all cases was from a third-party payer, although this was often described as ‘societal’. Time horizons were at least 20 years and only shorter when the life expectancy of people screened was significantly less than 20 years.

#### Test sensitivity and specificity

All studies included IGRA testing in the cost-effectiveness model, either in isolation or as part of a strategy including TST and/or CXR, and all but five included TST [10, 13, 36, 37, 39],as a comparator. When reported, sources of sensitivity and specificity were for IGRAs were largely from systematic reviews on test accuracy [41, 42]. With no gold standard, the specificities were derived from testing people from healthy low risk populations. There was variance in the presumed accuracy of tests between studies; for example, sensitivity of QFT ranged from just above 0.5 (although most commonly above 0.8) to 1.0. IGRAs were consistently modelled as having higher sensitivity and specificity than TST, with QFT was having slightly lower sensitivity but slightly higher specificity than T-SPOT. Only two studies used evidence on test results linked directly to the development of active TB rather than detection of LTBI [16, 17].

#### LTBI epidemiology

Prevalence of LTBI in the population of interest varied widely between studies from below 1% in children in Japan [40] to over 40% in migrants from high incidence countries [14]. Only one study included prevalence rates declining over time [21]. Secondary or onward transmission of LTBI from patients who develop active TB was included in five studies [11, 19–21, 27], with each active case assumed to infect between 0.2 and 0.31 people.

#### LTBI treatments, adverse events and TB mortality

All but four studies (which were only available as abstracts) [25, 30, 36, 38] stated the treatment for LTBI, and in all cases this was either INH or RIF in isolation or combination. Duration of treatment was not reported in most studies and where reported was either once weekly isoniazid for 12 weeks (3HP) [15, 21, 22, 26] or six or nine months isoniazid therapy (6H or 9H) [22, 26, 33, 38]. Twenty-seven studies considered adverse events of treatment [8–24, 26–29, 31–35, 37], notably INH related hepatoxicity (with nine considering hepatotoxicity-related death). The baseline mortality rate from active TB was approximately 5% in people with no underlying health conditions but varied by age and comorbidity.

#### Costs and utilities

In all but one study (where utilities were drawn from a trial underpinning the cost-effectiveness analysis), utilities were either drawn from published literature or assumed. Base case utilities for people without active TB were in all studies assumed to be 1.0, with decrements, where applied, of 0.01-0.03 for LTBI treatment. Active TB had utility values of between 0.4 and 0.8 depending on drug resistance and hospitalization status. Costs were drawn from published literature or national pricing bodies. IGRA tests were in all cases less than $85 per person and TST less than $20. The highest unit costs in models were treatment of isoniazid related hepatitis or active TB, with multi drug resistant TB when included costing over $100,000 to treat [16].

### Limitations of studies and quality assessment

The Ecobias economic evaluation checklist highlighted no substantive concerns in any included study in terms of overall bias or model specific bias (structure or data) beyond a weakness across all studies in either referencing sources correctly, justifying choices of parameter values chosen (such as discount rates and efficacy) or having to assume values for key parameters such as utility values, the prevalence of LTBI and LTBI activation rates. This was, as is usually the case, especially true for those studies where only abstracts were available. Full risk of bias assessments against the Ecobias tool can be found in Additional file 2: Appendix I.

### Summary of study cost-effectiveness findings by high-risk group

#### Migrants

Thirteen studies in fourteen publications [8–20] explored for cost effectiveness of testing for LTBI for migrants. Four studies in five publications [8–12] were on migrants entering the USA, three studies were on migrants entering Canada [13–15], two studies were on migrants entering the UK [16, 17], two studies on migrants entering Japan [18, 19] and there was a single study on migrants entering Oman [20]. Extraction tables for the modelling methods employed and parameter values used for studies on migrants are presented in Additional file 2: Appendix B. In all but the two UK studies [16, 17], IGRA compared to TST was found to be a dominant strategy (costing less and generating more QALYs) or have an ICER per QALY gained that would be considered cost-effective by pricing and reimbursement agencies in each country. The two UK studies [16, 17] found that TST could be the most cost-effective option, either alone [16] or in combination with an IGRA [17]. One UK study [17] included secondary transmission and evidence on sensitivity and specificity of tests to identify LTBI that would go on to become active TB. In all studies, results were sensitive to the prevalence of LTBI in the migrant population being screened.

#### People with HIV

Nine studies explored the cost effectiveness of testing for LTBI in people living with HIV [10, 19, 22–28]. Six of the studies were on people with HIV in the UK [22–27], two studies were people with HIV in Japan [19, 28] and one study was of people with HIV in the USA [10]. Extraction tables for the modelling methods employed and parameter values used for studies on people with HIV are presented in Additional file 2: Appendix C.

Between 2012 and 2020 six studies were produced by the same research team using data from a HIV clinic in London [22–27]. Rather than use specificity and sensitivity of tests from literature, all used actual data from testing results and long term outcomes for patients attending the clinic. The studies had a particular focus on assessing the National Institute for Health and Care Excellence (NICE) strategy for testing (IGRA + TST if CD4 < 500) and the BHIVA strategy (IGRA alone—If from Sub-Saharan Africa: Any blood CD4, duration ART < 2 years. Middle TB incidence country: Blood CD4 < 500, duration ART < 2 years. Low TB incidence country: Blood CD4 < 350, duration ART < 6 months). Whilst in the earlier studies [22–26] the NICE strategy (IGRA + TST) was cost-effective, over time as prevalence of LTBI has declined the authors concluded that testing all HIV patients in the UK is unlikely to be cost-effective regardless of testing strategy employed [27]. Nevertheless, testing of migrants with HIV from high or middle income countries may still be cost-effective [27].

The two studies in Japan [19, 28] concluded that IGRA alone or in combination with TST were likely the most cost-effective strategies for HIV populations in Japan who were prisoners or pregnant women. The one study in the USA [8, 9] found that IGRA was cost-effective compared to TST in people with HIV.

#### Contacts of people with active TB

Five studies in six papers explored the cost effectiveness of testing for LTBI in contacts of known positive cases of active TB [8, 9, 17, 19, 30, 31]. Two of the studies were in Japan [19, 31], two in the UK [17, 30] and one in the USA [8, 9]. Extraction tables for the modelling methods employed and parameter values used for studies on contacts are presented in Additional file 2: Appendix D. All studies concluded that either IGRA alone or in combination with TST were the most cost-effective testing strategies.

#### Immunocompromised

Six studies reported results for the immunocompromised population (ie with chronic conditions that make people more susceptible to infection or taking medication that suppress the immune system) [8, 9, 16, 32–34]. One study was carried out in Japan [29], two in the USA [8, 9, 33] and one each in Europe [34] and the UK [16]. Extraction tables for the modelling methods employed and parameter values used for studies on people who are immunocompromised are presented in Additional file 2: Appendix E. The findings from studies were somewhat conflicting, with cost-effectiveness of different testing strategies varying by the specific population.

In the study in Japan [32] nursing home residents with chronic kidney disease IGRA + TST was the most cost-effective strategy. Two US studies looking at idiopathic nephrotic syndrome in children [33] and patients on immunosuppressant medications [8, 9] found no testing strategy to be cost-effective. The latter finding is supported by a European study [34] who found that LTBI testing of patients commencing TNF-alpha therapy is not cost-effective but contradicted by the UK study [16] who found IGRA followed by TST the most cost-effective strategy.

#### Healthcare workers

Four included studies reported results for annual testing for LTBI of HCWs [21, 35–37]. One study was carried out in each of the following countries: UK [35], Japan [21] and Singapore [37]. The location in which the other study was carried out was not reported but presumably was Japan [36]. Extraction tables for the modelling methods employed and parameter values used for studies on healthcare workers are presented in Additional file 2: Appendix F. All studies found screening of healthcare workers for LTBI using IGRA was a cost-effective strategy.

#### Other high-risk populations

Three studies reported results for other high-risk populations not already covered [29, 38, 39]. Two studies were carried out in Japan [29, 38] and one in Hong Kong [39]. Extraction tables for the modelling methods employed and parameter values used for studies on other high-risk populations are presented in Additional file 2: Appendix G. The populations considered included nursing home residents, mental health inpatients, diabetics, prisoners and the homeless. In all instances except for diabetics and prisoners (where no testing was cost-effective), testing with IGRA was the most cost-effective strategy.

#### Children

Two studies reported results for LTBI testing in children (not already covered previously as being specific high-risk populations) [16, 40]. One of the studies was in Japan [40] and one in the UK [16]. Extraction tables for the modelling methods employed and parameter values used for studies on children are presented in Additional file 2: Appendix H. In the study in Japan [40] it was found that if immunocompetent 16 year olds were tested, then IGRA was the most cost-effective strategy. This contrasts slightly with the UK study [16] that reported that if five year olds in the UK were to be tested for LTBI, IGRA + TST was the most cost-effective testing strategy.

## Discussion

Whilst some concerns remain around uncertainty in key LTBI/TB parameters such as TB activation rates and LTBI/TB utility values, this review would agree with the findings of previous literature reviews of LTBI testing—that the studies available are robust enough to draw conclusions on the relative cost-effectiveness of different LTBI testing strategies at least in high income/low burden countries.

Highlighted by studies that looked at populations with differing risk, the cost-effectiveness of any testing strategy is closely linked to the prevalence of LTBI in a population with the higher the prevalence, the more likely testing will be cost-effective. Including secondary transmission in models did not materially impact ICERs in studies where sensitivity analysis removed or included onward transmission. Evidence on the predictive value of tests for development of active TB is still lacking (included in only two studies with conflicting findings on the cost-effectiveness of IGRA compared to TST).

Where QFT was compared to T-SPOT, QFT was always more cost-effective [12, 18–20, 29–32, 38]. For lower risk populations including children without increased risk factors then TST or no testing may be the most cost-effective strategy [16].

In summary, whilst there are still weaknesses in the available data to assess the cost-effectiveness of different tests for LTBI which was pointed out in previous systematic reviews, findings were consistent in suggesting that IGRAs are cost-effective in high-income settings for high-risk populations—even despite variation in model structure and parameter estimates. More accurate estimates of sensitivity and specificity with respect to predicting active TB and of utility values for people with LTBI, being treated for LTBI and with active TB are important areas for future research to refine these estimates. Evidence is also required on the cost-effectiveness of different strategies in low to middle income countries and countries with high TB burden.

## Supplementary Information

**Additional file 1.** Search strategy.

**Additional file 2.** Full extraction tables.

## Data Availability

All studies included in the systematic review are included in the reference list.
