# Supplementary material for: A systematic review of cost-utility analyses of screening methods in latent tuberculosis infection in high-risk populations
Source: BMC Pulm Med. 2022 Oct 5;22:375. doi: 10.1186/s12890-022-02149-x (PMC9533619; doi:10.1186/s12890-022-02149-x)
Supplement: Supplementary file 1 — Additional file 1. Search strategy. [file 12890_2022_2149_MOESM1_ESM.docx]

### APPENDIX A

### Search strategy

A MEDLINE (OvidSP) search strategy was designed to identify studies of the three eligible screening tests for TB (including LTBI). The final MEDLINE strategy is presented in Figure A.1.

The main structure of the strategy comprised five concepts:

TB (search lines 1 to 8)

Interferon gamma release assay (IGRA) test (including QuantiFERON / T-SPOT) (search lines 9 to 28)

Tuberculin skin test (search lines 29 to 36)

Chest X-ray (search lines 37 to 47)

Economic evaluations (search lines 54 to 70)

The concepts were combined as follows: TB AND (IGRA test OR tuberculin skin test OR chest X-ray) AND economic evaluations.

The performance of the terms for the three specific screening tests in the above search approach was tested by checking retrieval of records for studies included in five systematic reviews (two economic, three clinical) on topics related to the tests of interest. In total across the five reviews 95 unique papers were included and were available to be found in MEDLINE. The terms for the three specific screening tests retrieved records for all 95 papers. Although this suggested that database records on the interventions of interest would explicitly refer to the interventions at database record level, this was based on just a small sample of studies. The research team judged it was possible that a relevant study might refer to a test of interest at full text level but only refer to *non-specific* TB screening at database record level. The strategy therefore also included a supplementary focused search approach on non-specific TB screening (search lines 49 to 53). The concepts for this supplementary approach were combined as follows: non-specific TB screening AND economic evaluations.

The strategy was devised using a combination of subject indexing terms and free text search terms in the Title, Abstract and Keyword Heading Word fields. The search terms for population and intervention concepts were identified through discussion within the research team, scanning background literature and browsing database thesauri.

The search terms for the economic evaluations concept (search lines 54 to 70) used the filter developed by the University of York Centre for Reviews and Dissemination (CRD) for identification of economic evaluations to include in NHS Economic Evaluation Database (NHS EED) [5].

The strategy excluded animal studies from MEDLINE using a standard algorithm (search line 74).

The strategy was restricted by date to studies published from 2011 to date. QIAGEN identified this date as appropriate for the identification of new research published since the date of the 2011 systematic review by Nienhaus et al [6]. No language restrictions were applied.

The performance of the strategy was tested by checking retrieval of records for the 28 studies included in the 2016 systematic review by Koufopoulou et al (on methods used in economic evaluations of tuberculin skin tests and interferon gamma release assays) [7], the 13 studies included in the 2011 systematic review by Nienhaus et al (on cost and cost-effectiveness of different TB-screening strategies) [6] and 21 studies supplied by QIAGEN (51 unique records in total). Before the date restriction was applied, the strategy retrieved records for 48/51 of the included papers. The records for the three non-retrieved papers did not include economics or cost terms. The three non-retrieved papers were assessed by the research team and were judged ineligible for inclusion in this review.

The final Ovid MEDLINE strategy was peer-reviewed by a second Information Specialist for errors in spelling, syntax and line combinations.

Figure A.1: Final search strategy for MEDLINE ALL

1 exp Tuberculosis/ (193406)

2 Mycobacterium tuberculosis/ (51073)

3 (tuberculosis or tuberculoses).ti,ab,kf. (217050)

4 tb.ti,ab,kf. (57829)

5 (mdrtb or xdrtb or ltb or ltbi or ltbis).ti,ab,kf. (4229)

6 (tuberculous or tubercular or tuberculoma or tuberculomas or tuberculid or tuberculosa).ti,ab,kf. (39395)

7 (koch$ adj (disease$ or disorder$)).ti,ab,kf. (10)

8 or/1-7 (283194)

9 Interferon-gamma Release Tests/ (1523)

10 ((interferon or ifn) adj2 release).ti,ab,kf. (4107)

11 ((interferongamma or interferong or interferony) adj2 release).ti,ab,kf. (8)

12 ((ifngamma or ifng or ifny) adj2 release).ti,ab,kf. (188)

13 ((gammainterferon or ginterferon or yinterferon) adj2 release).ti,ab,kf. (0)

14 ((gammaifn or gifn or yifn) adj2 release).ti,ab,kf. (1)

15 ((interferon-gamma or interferongamma or interferon-g or interferong or interferon-y or interferony) adj11 (assay$ or detect$ or diagnos$ or screen$ or test or tests or testing or testings or tested)).ti,ab,kf. (5970)

16 ((ifn-gamma or ifngamma or ifn-g or ifng or ifn-y or ifny) adj11 (assay$ or detect$ or diagnos$ or screen$ or test or tests or testing or testings or tested)).ti,ab,kf. (11862)

17 ((gamma-interferon or gammainterferon or g-interferon or ginterferon or y-interferon or yinterferon) adj11 (assay$ or detect$ or diagnos$ or screen$ or test or tests or testing or testings or tested)).ti,ab,kf. (1063)

18 ((gamma-ifn or gammaifn or g-ifn or gifn or y-ifn or yifn) adj11 (assay$ or detect$ or diagnos$ or screen$ or test or tests or testing or testings or tested)).ti,ab,kf. (1614)

19 (igra or igras).ti,ab,kf. (1375)

20 or/9-19 (19020)

21 quantiferon$.ti,ab,kf. (1950)

22 quanti-feron$.ti,ab,kf. (15)

23 qft$.ti,ab,kf. (1258)

24 or/21-23 (2106)

25 Enzyme-Linked Immunospot Assay/ (1226)

26 (enzyme-linked immunospot$ or enzyme linked immunosorbent spot$ or elispot$).ti,ab,kf. (6646)

27 (t-spot$ or tspot$).ti,ab,kf. (673)

28 or/25-27 (7560)

29 Tuberculin Test/ (13798)

30 (tuberculin$ adj11 (allerg$ or assay$ or detect$ or diagnos$ or hypersensitivit$ or negativ$ or positiv$ or reaction$ or reactivit$ or response$ or screen$ or sensitivit$ or test or tests or testing or testings or tested)).ti,ab,kf. (13344)

31 (tuberculum$ adj11 (allerg$ or assay$ or detect$ or diagnos$ or hypersensitivit$ or negativ$ or positiv$ or reaction$ or reactivit$ or response$ or screen$ or sensitivit$ or test or tests or testing or testings or tested)).ti,ab,kf. (62)

32 (pirquet$ adj11 (allerg$ or assay$ or detect$ or diagnos$ or hypersensitivit$ or negativ$ or positiv$ or reaction$ or reactivit$ or response$ or screen$ or sensitivit$ or test or tests or testing or testings or tested)).ti,ab,kf. (90)

33 ((mantoux$ or endotuberculin$ or heaf$ or purified protein derivative$ or purified protein derivate$ or tine) adj11 (allerg$ or assay$ or detect$ or diagnos$ or hypersensitivit$ or injection$ or negativ$ or positiv$ or reaction$ or reactivit$ or response$ or screen$ or sensitivit$ or test or tests or testing or testings or tested)).ti,ab,kf. (3876)

34 (tuberculin skin$ or aplisol$2 or aplitest$2 or mono-vacc$2 or monovacc$2 or tubersol$2 or tubertest$).ti,ab,kf. (4909)

35 (tst or tsts or ((ppd or ppdb or ppdcg or ppdf or ppdl or ppds) adj11 (allerg$ or assay$ or detect$ or diagnos$ or hypersensitivit$ or negativ$ or positiv$ or reaction$ or reactivit$ or response$ or screen$ or sensitivit$ or test or tests or testing or testings or tested))).ti,ab,kf. (11095)

36 or/29-34 (21094)

37 radiography, thoracic/ or mass chest x-ray/ (34019)

38 (Radiography/ or X-Rays/) and Mass Screening/ (949)

39 exp Thorax/dg (4115)

40 exp Lung/dg (27482)

41 (exp Thorax/ or exp Lung/) and X-Rays/ (613)

42 (exp Thorax/ or exp Lung/) and Radiography/ (12781)

43 ((chest$ or lung$ or thorac$ or thorax$) adj6 (grenz-ray$ or grenzray$ or radiogram$ or radiograph$ or radiolog$ or roentgen$ or x-radiation$ or xradiation$ or x-ray$ or xray$)).ti,ab,kf. (67768)

44 ((grenz-ray$ or grenzray$ or radiogram$ or radiograph$ or radiolog$ or roentgen$ or x-radiation$ or xradiation$ or x-ray$ or xray$) adj6 (assay$ or detect$ or diagnos$ or screen$ or test or tests or testing or testings or tested)).ti,ab,kf. (91844)

45 (thoraxradiograph$ or x-chest$).ti,ab,kf. (30)

46 (cxr or cxrs).ti,ab,kf. (2335)

47 or/37-46 (192103)

48 (8 and (20 or 24 or 28 or 35 or 47)) or 36 (34650)

49 (exp Tuberculosis/ or Mycobacterium tuberculosis/) and Mass Screening/ (3078)

50 Tuberculosis/di, dg or Tuberculosis, Pulmonary/di, dg or Latent Tuberculosis/di, dg or Mycobacterium tuberculosis/di (32000)

51 ((tuberculosis or tuberculoses or tb or mdrtb or xdrtb or ltb or ltbi or ltbis or tuberculous or tubercular or tuberculoma or tuberculomas or tuberculid or tuberculosa or koch$ disease$ or koch$ disorder$) and (assay$ or detect$ or diagnos$ or screen$ or test or tests or testing or testings or tested)).ti. (22246)

52 ((tuberculosis or tuberculoses or tb or mdrtb or xdrtb or ltb or ltbi or ltbis or tuberculous or tubercular or tuberculoma or tuberculomas or tuberculid or tuberculosa or koch$ disease$ or koch$ disorder$) adj6 (assay$ or detect$ or diagnos$ or screen$ or test or tests or testing or testings or tested)).ab,kf. (38904)

53 or/49-52 (65366)

54 Economics/ (27272)

55 exp "costs and cost analysis"/ (240582)

56 Economics, Dental/ (1913)

57 exp economics, hospital/ (24831)

58 Economics, Medical/ (9110)

59 Economics, Nursing/ (4001)

60 Economics, Pharmaceutical/ (2960)

61 (economic$ or cost or costs or costly or costing or price or prices or pricing or pharmacoeconomic$).ti,ab. (827619)

62 (expenditure$ not energy).ti,ab. (30809)

63 value for money.ti,ab. (1759)

64 budget$.ti,ab. (30153)

65 or/54-64 (981445)

66 ((energy or oxygen) adj cost).ti,ab. (4177)

67 (metabolic adj cost).ti,ab. (1461)

68 ((energy or oxygen) adj expenditure).ti,ab. (25621)

69 or/66-68 (30268)

70 65 not 69 (974498)

71 48 and 70 (1132)

72 53 and 70 (3386)

73 71 or 72 (3653)

74 exp animals/ not humans/ (4762182)

75 73 not 74 (3497)

76 limit 75 to yr="2011 -Current" (2022)

Key to Ovid symbols and commands

$ Unlimited right-hand truncation symbol

ti,ab,kf Searches are restricted to the Title (ti), Abstract (ab) and Keyword Heading Word (kf) fields

adj Retrieves records that contain terms next to each other (in the shown order)

adjN Retrieves records that contain terms (in any order) within a specified number (N) of words of each other

/ Searches are restricted to the Subject Heading field

exp The subject heading is exploded

pt. Search is restricted to the publication type field

or/1-7 Combines sets 1 to 7 using OR

.fs. Term is searched as a floating subheading

We conducted the literature search in the databases and information resources shown in Table A.1

Table A.1: Databases and information sources searched

| Resource | Interface / URL |
| --- | --- |
| MEDLINE ALL | OvidSP |
| Embase | OvidSP |
| Database of Abstracts of Reviews of Effects (DARE) | https://www.crd.york.ac.uk/CRDWeb |
| HTA Database | https://database.inahta.org/ |
| NHS Economic Evaluation Database (NHS EED) | https://www.crd.york.ac.uk/CRDWeb |
| EconLit | OvidSP |
| National Institute for Health and Care Excellence (NICE) webpages | https://www.nice.org.uk/ |
| Canadian Agency for Drugs and Technologies in Health (CADTH) webpages | https://www.cadth.ca/ |
| Institute for Clinical and Economic Review (ICER) webpages | https://icer-review.org/ |
| European Congress of Clinical Microbiology & Infectious Diseases (ECCMID) 2018 | See Appendix |
| European Congress of Clinical Microbiology & Infectious Diseases (ECCMID) 2019 | See Appendix |
| European Congress of Clinical Microbiology & Infectious Diseases (ECCMID) 2020 | See Appendix |
| Union World Conference on Lung Health 2018 | See Appendix |
| Union World Conference on Lung Health 2019 | See Appendix |
| Union World Conference on Lung Health 2020 | See Appendix |
| North America Region (NAR) TB conference 2018 | See Appendix |
| North America Region (NAR) TB conference 2019 | See Appendix |
| North America Region (NAR) TB conference 2020 | See Appendix |

In addition to searching the HTA database, targeted searches of the technology assessment and regulatory agency websites listed above (NICE, CADTH and ICER) were conducted.

Recent research published as conference abstracts was identified by searching Embase (which indexes a significant number of conference publications). The following three conferences were identified by QIAGEN as highly relevant.

European Congress of Clinical Microbiology & Infectious Diseases (ECCMID)

Union World Conference on Lung Health

North America Region (NAR) TB conference

We checked if records for these conferences were included in Embase for the last three years (2018 to 2020). Records were not found in Embase for any of the conferences. We therefore sought the abstracts via webpages or journal supplements, where they were available online free of charge.

We also checked the reference lists of any included studies and relevant systematic reviews published in the last 5 years for any eligible studies that might have been missed by the database searches.
