# Supplementary material for: A systematic review of cost-utility analyses of screening methods in latent tuberculosis infection in high-risk populations
Source: BMC Pulm Med. 2022 Oct 5;22:375. doi: 10.1186/s12890-022-02149-x (PMC9533619; doi:10.1186/s12890-022-02149-x)
Supplement: Supplementary file 2 — Additional file 2. Full extraction tables. [file 12890_2022_2149_MOESM2_ESM.docx]

# APPENDIX B

# Included studies full extraction tables: Migrants

Table B.1: Migrants: Population information and tests considered

| Author/ Trial ID | Country/region | Description of population | Tests assessed | Prevalence rate of LTBI | BCG vaccination rate | TB activation rate | Secondary transmission rate |
| --- | --- | --- | --- | --- | --- | --- | --- |
| Abubakar 2018 [16] | UK | Migrants | 1. QFT with 4R 2. QFT with 6H  3. QFT with 3HP 4. TST with 4R 5. TST with 6H 6. TST with 3HP 7. CXR | TB incidence: 0.000099 LTBI: 0.43 MDR-TB: 0.021 | NR | NR | NR |
| Al Abri 2020 [19] | Oman | Recent arrivals | TST QFT-GIT T-SPOT.TB CXR | Base case (range for SA) 0.0237 (0.0150-0.0345) | NR | Proportion still infected post-LTBI treatment: 0.345 Average number of secondary cases from one index case: 0.2 (0.1-0.3) Average delay from infection to activation (secondary cases): 2.88 | Proportion still infected post-LTBI treatment: 0.345 Average number of secondary cases from one index case: 0.2 (0.1-0.3) Average delay from infection to activation (secondary cases): 2.88 |
| Auguste 2016 [15] | UK | New permanent residents to Canada (n=260,600). Base case focuses on 6100 who were flagged for post-landing medical surveillance | TST/INH TST/RIF IGRA/INH IGRA/RIF SEQ/INH SEQ/RIF  (SEQ=BC+TST at time of medical examination) No intervention | Prevalence in country of origin (cases per 100,000) Population under surveillance <30: 0.0641 30-99: 0.1862 100-199: 0.3659  ≥200: 0.3420  Population not under surveillance <30: 0.0159 30-99: 0.0902 100-199: 0.2016 ≥200: 0.3162 | BCG vaccination (<30 cases) : 0.605 BCG vaccination (≥30 cases): 0.938 BCG vaccination uptake: 0.616 | Reactivation: 0.0011 (0.0009-0.0013) Risk increase if abnormal: 3.9 (2.7-5.5) Extended therapy: 0.124 (0.0-0.3) Relapse rate: 0.0359 (0.0274-0.0462) | NR |
| Campbell 2017 [12] | Canada | Prospective migrants | Base case (no screening or treatment for LTBI) IGRA/INH TST/INH | LTBI prevalence Low incidence: 0.336 (0.274-0.0400) Moderate incidence: 0.203 (0.187-0.219) High incidence: 0.159 (0.1706-0.2464) Very high incidence: 0.3162 (0.2686-0.3880) | BCG vaccination (<30 cases): 0.605 BCG vaccination (≥30 cases): 0.998 BCG vaccination uptake: 0.837 | NR | NR |
| Campbell 2019 [13] | Canada | Persons who obtained a Canadian permanent resident status visa between 1985 and 2012 and who became residents of British Columbia at any time between 1985 and 2013 with late-stage CKD diagnosed or who initiated dialysis therapy and did not have previous TB, LTBI screening, or LTBI treatment and did not undergo transplantation previously or within the subsequent 30 days | Base case (BC): CXR+medical history+symptom screen BC+TST/INH BC+TST/RIF BC+IGRA/INH BC+IGRA/RIF SEQ/INH (SEQ=BC+TST at time of medical examination) SEQ/RIF | LTBI prevalence Low incidence: 0.0159 (0.0135-0.0195) Moderate incidence: 0.0902 (0.1763-0.1102) High incidence: 0.2016 (0.1706-0.2464) Very high incidence: 0.3162 (0.2686-0.3880)  Abnormal CXR results or previous TB Low incidence: 0.039 (fixed) Moderate incidence: 0.029 (fixed) High incidence: 0.028 (fixed) Very high incidence: 0.039 (fixed)  Adherence to post arrival follow-up: 0.684 (0.646-0.721) | BCG vaccination (<30 cases): 0.605 (0.60-0.61) BCG vaccination (≥30 cases): 0.998 (0.997-0.999) BCG vaccination uptake: 0.837 (fixed) | 0.0011 (0.0009-0.0013)  Abnormal CXR risk change: 3.9 (3.0-4.9) Extended therapy: 0.124 (0.029-0.264) Relapse rate: 0.0359 (0.0197-0.0654) Hospitalisation duration: 17 days (fixed) | NR |
| Campbell 2019a [14] | Canada (British Columbia) | Base case: Annual testing of HCW and 2% of population (chosen at random) Four strategies involving increasing targeted testing and treatment: Strategy 1: increase in individuals with medical risk factors Strategy 2: increase in all non-US born Strategy 3: increase in all non-US born and those with medical risk factors regardless of where born Strategy 4: universal testing and treatment for all Californians | QFT, TST | Prevalence of LTBI estimated for US born and non-US born for the overall population and by sex (M/F), race/ethnicity (5 categories) and age group (14 groups) Totals US born: 2.4% Non-US born: 19.4%   Max: 30.1% (Asian, non-US born) Min: 0.4% (US born, aged 15-19 years)  Prevalence of LTBI was also varied over time by US born and non-US Born (2001, 2014, 2030, 2040 and 2050) | NR | RR of reactivation for risk factors Diabetes: 1.6 (1.3-3.6) Smoking: 2.5 (1.0-4.0) HIV: 5.4 (2.9-22) TNF-alpha: 4.7 (2.5-5.3) Solid-organ transplants: 2.4 (1.7-18) ESRD: 11 (2.0-20) | NR |
| Goodell 2019 [11] | California, USA | Immunocompetent expatriates from low incidence countries returning from long-term residence in high-incidence countries | QFT, TSPOT, TST, CXR | TB: 0.00039 LTBI: 0.03 MDR-TB: 0.036 | 0.94 | NR | NR |
| Kowada 2016 [17] | Japan | Ex pats returning from high incidence countries | TST QFT T-SPOT TST/QFT TST/T-SPOT CXR | Incidence of TB among 20 year old HIV+ve pregnant women in postpartum period: 0.0003 (0.00002-0.00065) TB risk during pregnancy: 1.29 (95% cI: 0.82-2.03) TB risk in postpartum period: 1.95 (1.24-3.07) | NR | Increased likelihood of progression from LTBI to active TB among HIV positive pregnant women: 4.5 (95%CI: 1.1-18.0) | NR |
| Kowada 2014 [18] | Japan | Four non-US born risk populations: with no comorbidities with diabetes with HIV with ESRD | • No testing • TST • IGRA • Confirm positive (initial TST, IGRA only for TST-positive results; both tests positive indicates LTBI) • Confirm negative (initial IGRA, then TST for IGRA-negative; any test positive indicates LTBI) • All strategies were coupled to treatment with 3 months of self-administered rifapentine and isoniazid | LTBI prevalence: 0.159 (range evaluated: 0.0 - 1.0) | NR | Base case value (range evaluated) Reduction in reactivation probability after complete therapy: 0.900 ( 0.5 - 1.0) | 0.25 (0.1-1.0) |
| Tasillo 2017 [9] | USA | Migrants (non-US born), diabetic, HIV, homeless, incarcerated | IGRA | 24%-43% (California only). Other States not provided | NR | NR | NR |
| Jo 2020 [10] | USA | Non USA born populations | TST, IGRA | Adult: 41.4% Children: 7.0% | NR | Adult: 0.079 per 100 person years.  Children: 0.079 per 100 person years Reduced by 10% per decade | 0.31 |
| Linas 2011 [7,8] | USA | Migrants | 1. QFT with 4R 2. QFT with 6H  3. QFT with 3HP 4. TST with 4R 5. TST with 6H 6. TST with 3HP 7. CXR | TB incidence: 0.000099 LTBI: 0.43 MDR-TB: 0.021 | NR | NR | NR |

Key: 3HP - once-weekly isoniazid- rifapentine for 12 weeks; 4R – 4 months rafimpin; 6H - 6 months isoniazid monotherapy; BCG - Bacillus Calmette–Guérin; CXR – Chest X-ray; IGRA - Interferon Gamma Release Assay; INH – isoniazid; LTBI – Latent tuberculosis infection; MDR – multi drug resistant; NR – Not recorded; QFT – QuantiFERON; RIF – rafimpin; TSPOT - T-Spot TB test; TST – Tuberculin Skin Test

Table B.2: Migrants: Modelling methods

| Author/ Trial ID | Model type | Time horizon | Health states | Perspective | Discounting |
| --- | --- | --- | --- | --- | --- |
| Abubakar 2018 [16] | Decision tree and patient level discrete event simulation model | Lifetime | **Decision tree (IGRA alone example)** Result: determinate/indeterminate For determinate result: +ve/-ve IGRA For +ve IGRA: +ve/-ve chest radiograph For +ve radiograph: active TB and treated: adherence/non-adherence or no active TB (LTBI) For -ve chest radiograph: accept/refuse CPX For accept CPX: adherence/non-adherence  **DES model** Active TB on treatment Active TB not on treatment No TB and on CPX (chemoprophylaxis) No TB and non on CPX | NHS | 3.5% pa (costs and benefits) |
| Al Abri 2020 [19] | Decision trees with Markov models | Lifetime | Decision tree **TST with 3HP/6H/4R** TST +ve/-ve CXR +ve/-ve 3HP/6H/4R adherence/non-adherence 3HP/6H/4R complication/no complication  **QFT-Plus with 3HP/6H/4R** QFT-Plys +ve/-ve CXR +ve/-ve 3HP/6H/4R adherence/non-adherence 3HP/6H/4R complication/no complication  CXR **CXR +ve/-ve  Markov model** 1. Well (no LTBI and no TB) 2. LTBI 3. LBTI taking treatment without complications 4. LBTI taking treatment with complications 5. TB 6. MDR-TB 7. Death | Omani healthcare sector | NR |
| Auguste 2016 [15] | Decision tree followed by DES | 100 years | **Decision tree**  Determinate/indeterminate result (TST only) +ve/-ve tests in isolation or combination  +ve/-ve CXR Gastric lavage Active TB and treated/no active TB (LTBI) Treatment adherence/non-adherence Accept/refuse LTBI treatment LTBI treatment adherence/non-adherence  **Dynamic transmission model** No LTBI/TB LTBI Active TB Resolved TB Secondary infections Death all causes Death TB | NHS and Personal Social Services | 3.5% costs and outcomes |
| Campbell 2017 [12] | DES | 10 years | Flagged for surveillance/not flagged for surveillance Adherent with surveillance Complete LTBI screening Complete medical evaluation LTBI treatment initiation Adverse event Death due to adverse event Imported TB Non-adherent with surveillance Fail to complete screening Fail to complete medical evaluation or screening test -ve Fail to initiate treatment Treatment default Adequately complete treatment Healthy LTBI TB Dead | Canadian healthcare system | 1.5% for costs and outcomes |
| Campbell 2019 [13] | DES | 5 years | Late stage CKD TB Dialysis Death | Canadian healthcare system | 1.50% |
| Campbell 2019a [14] | DES | 25 years | Pre-immigration medical exam Chest radiograph and medical evaluation performed Intervention LTBI diagnostic test placed +ve test/-ve test Flag for post-immigration LTBI treatment Patient arrives in destination country Patient successfully adheres and reports post-immigration Fail to initiate treatment Initiate LTBI treatment Patient experiences and AE during treatment Death Treatment cessation Patient successfully completes full course of LTBI treatment Arrives in destination country No further intervention, simulated to time horizon Patient partially completes LTBI treatment before defaulting Patient fails to adhere and does not report post-immigration Arrives in destination country | Canadian healthcare system | 3% for costs and outcomes |
| Goodell 2019 [11] | Locally interacting Markov chains | 48 years | TB health states (n=68) representing all the disease states and testing/treatment possibilities, states for six medical risk factors and age group, sex, race, length of time in US and country of birth | NR. Appears to be health service | 3% for costs and benefits |
| Kowada 2016 [17] | Decision tree followed by a markov model. | Lifetime | Decision tree Test result  CXR if +ve  LTBI treatment adherence w/wout complication   Markov model Healthy (no TB and no LTBI) LTBI TB (drug sensitive or MDR) Dead | Societal | 3%pa (costs and benefits) |
| Kowada 2014 [18] | Decision tree followed by a Markov model. | 30 years | Decision tree Test result  CXR if +ve  LTBI treatment adherence w/wout complication   Markov model Healthy (no TB and no LTBI) LTBI TB (drug sensitive or MDR) Dead | Public health payer | 3% for costs and benefits |
| Tasillo 2017 [9] | Decision trees with Markov models | Lifetime (years NR) | Decision tree captures the prevalence of LTBI, probability of testing positive and the probability of initiating treatments. Patients may be lost to follow-up before TST. After the decision tree patients enter the Markov mode: LTBI with treatment, LTBI without treatment, no LTBI with treatment, and no LTBI without treatment | US Healthcare perspective | 3% for costs and outcomes |
| Jo 2020 [10] | Unclear. A four state model but unclear whether a Markov or patient level simulation model | 30 year | Uninfected LTBI Active TB Successfully treated | State healthcare system | 3% pa (costs and benefits) |
| Linas 2011 [7,8] | Decision trees with Markov models | Lifetime | **In decision tree (nodes)** Test result (positive or negative) Adherence to therapy Complications from therapy  **In Markov mode**l INH therapy without toxicity Non-fatal INH hepatitis Active TB Post Active TB Death | Health care costs | 3% pa (costs and benefits) |

Key: 3HP - once-weekly isoniazid- rifapentine for 12 weeks; 4R – 4 months rafimpin; 6H - 6 months isoniazid monotherapy; BCG - Bacillus Calmette–Guérin; CXR – Chest X-ray; DES – discrete event simulation; IGRA - Interferon Gamma Release Assay; INH – isoniazid; LTBI – Latent tuberculosis infection; MDR – multi drug resistant; NR – Not recorded; QFT – QuantiFERON; RIF – rafimpin; TSPOT - T-Spot TB test; TST – Tuberculin Skin Test

Table B.3: Migrants: Cost and utility parameter values

| Author/ Trial ID | Sources of utility values | Utility values used in model (mean (SD)) | Cost year | Currency | Source of test costs | Test costs in model | Sources of treatment costs | Treatment costs in model | Sources of other costs | Other costs in model |
| --- | --- | --- | --- | --- | --- | --- | --- | --- | --- | --- |
| Abubakar 2018 [16] | Published literature (Guo et al 2009; Dion et al 2004) | Well (no LTBI and no TB): 1 LTBI: 1 LTBI taking treatment without complications: 0.99 LTBI taking treatment with complications: 0.85 TB: 0.8 MDR-TB: 0.58 Dead: 0 | 2020 | Omani riyals (OMR) converted to US dollars (1OMR=USD2.60) | Omani sources | QFT-Plus: 20.5 TST: 23.5 CXR: 26.0 | Omani sources | 6H: 99.7 3HP with DOT: 369.20 4R: 100.8 Treatment of non MDR-TB: 8,038.4 | Kowada 2016 | Treatment of drug-induced hepatotoxicity by LTBI treatment: 4,576 |
| Al Abri 2020 [19] | Kowada | **Decrements** Active TB (while on treatment): 0.15 Treatment for LTBI: 0.001 | 2012/13 | GB£ | Pooran et al and NHS Reference costs 2012/13 | TST: 17.48 QFT-GIT: 48.73 T-SPOT.TB: 59.57 CXR:35.00 | Bothamley et al NHS drug tariff Pareek et al | Adherence to active TB treatment: 5461.12 Non-adherence to active TB treatment: 910.19 Adherence to LTBI treatment: 677.07 Non-adherence to LTBI treatment: 112.85 Treatment of isoniazid-induced hepatitis: 389.51 | NHS Reference Costs 2012/13 | Sputum examination: 7.00 |
| Auguste 2016 [15] | Published literature (Bauer et al 2015, Guo 2008, Marra 2008) | LTBI: 0.81 Healthy: 0.81 AE disutility: 0.2 TB: 0.69 Hospitalisation: 0.5 Dead: 0 | 2016 | Can$ | Literature | Complete TST: $31 Incomplete TST: $21 IGRA: $54  CXR: $35 | Literature (plus, for TB, expert opinion) | Full INH: $992 Full RIF: $575 Partial INH: $462 Partial RIF: $319  TB: $20,532 | Literature | LBTI AE: $732 Hospitalisation: $6641 Death: $26,933 |
| Campbell 2017 [12] | Published literature (Davidson et al, Yang et al, Bauer et al, Holland et al, Awan et al) | CKD: 0.66 Dialysis: 0.62 LTBI adjustment: 1 AE adjustment: 0.8 Hospitalisation: 0.4 Dead: 0 | 2016 | Can$ | Literature | Complete TST: $31 Incomplete TST: $21 IGRA: $54 CXR: $42 | Literature | LTBI costs Isoniazid: $992 Partial isoniazid treatment: $462 | Literature | LTBI costs LTBI adverse event: $732 LTBI hospitalisation: $6,641  TB costs Drug, workup, follow-up costs $1,620 Contact tracing (per contact): $369 Cost per hospitalisation: $15,740 TB with hospitalisation CKD: $40,111 TB with hospitalisation Dialysis: $46,015 TB without hospitalisation CKD: $8,631 TB without hospitalisation Dialysis: $14,535 |
| Campbell 2019 [13] | Published literature (Holland 2009, Marra 2008, Bauer 2015) | LTBI: 0.81 Healthy: 0.81 AE disutility: 0.2 TB: 0.69 Hospitalisation: 0.5 Dead: 0 | 2016 | Can$ | Literature | Complete TST: $31 Incomplete TST: $21 IGRA: $54 CXR: $42 | Literature (plus, for TB, expert opinion) | Full INH: $992 Full RIF: $575 Partial INH: $462 Partial RIF: $319  TB: $20,532 | Literature | LBTI AE: $732 Hospitalisation: $6641 Death: $26,933 |
| Campbell 2019a [14] | Miller 2004, calculated | QALYs lost per TB case (no medical risk factors): 1.7 (1.3-2.6) QALYs lost per TB case (medical risk factors): 1.1 (0.83-1.7) | NR | US$ | Centers for Medicare and Medicaid Services | TST: 9.51 (8.37-10.59) QFT: 84.35 (74.00-105.44) | Oh, 2015 Centers for Medicare and Medicaid Services Curry International TB Center and California Department of Public Health | Cost of active TB case (California): 31, 400 (13,377-39,250) 6H: 431.47 (323.60-539.34) 3HP: 840.64 (630.48-1050.80) | NR | NR |
| Goodell 2019 [11] | Published sources (Guo 2009, Dion 2002, Dion 2004) | Well: 1.00 LTBI: 1.00 LTBI with treatment without complication: 0.99 LTBI with treatment with complication: 0.85 Active non-MDR TB during treatment and before: 0.80 Active MDR-TB during treatment and before: 0.58 | 2012 | US$ | Medical insurance reimbursement table in Japan | QFT: $60.6 TSPOT: $60.6 TST: $15.4 CXR: $36.3 | Medical insurance reimbursement table in Japan. Published sources (Holland 2009) | 9H for LBTI: $773.6 3HP for LBTI: $503.5 non-MDR TB for 6 months: $14,879 MDR-TB: $192,921.1 Drug-induce hepatoxicity by LBTI: $11,903 | Ministry of Health, Labor and Welfare. Basic survey on wage structure | Smears, cultures and drug sensitivity tests: $159.7 Physician income per hour: $52.6 Radiologist and lab technician income per hour: $23.0 |
| Kowada 2016 [17] | Guo 2009 Resch 2006 | Non-LTBI and non-TB in HIV +ve pregnant state: 1 LTBI taking no chemoprophylaxis in HIV positive pregnant state: 1 LTBI taking chemoprophylaxis without complication in HIV positive pregnant state: 0.99 LTBI taking chemoprophylaxis with liver dysfunction in HIV positive pregnant state: 0.85 Active non MDR-TB during treatment and before in HIV +ve pregnant state: 0.80 Active MDR-TB during treatment and before in HIV positive pregnant state: 0.58 Dead: 0 | 2012 | US$ | Medical insurance reimbursement table | QFT: 60.6 (30.3-120.2) T-SPOT: 60.6 (30.3-120.2) TST: 15.4 (7.7-30.8) CXR: 36.3 (18.2-61.6) | Kowada 2008 Resch 2006 | INH chemoprophylaxis for 6 months: 515.7 (257.9-1,035.4) Treatment of INH-induced hepatitis by chemoprophylaxis: 11,903 (5951.5-23,806) Treatment of non MDR-TB for 6 months: 14,879 (7440-29578) Treatment of MDR-TB: 192,921 (96,461-385842) | Medical insurance reimbursement table | Smears and cultures of sputum examination: 69.9 (35.0-139.8) |
| Kowada 2014 [18] | LTBI and post-TB quality of life: assumptions Hepatotoxicity and active TB: literature | Base case (range evaluated) LTBI: 1 (0.99-1) Hepatotoxicity: 0.750 (0.6-1.0) Active TB: 0.830 (0.75-1.0)  Post-TB quality of life: 1 (0.87-1.0) | 2015 | US$ | Published literature | Base case (range evaluated) TST: 7.870 (5-15) IGRA: 84.350 (50-100) | Published literature | Base case (range evaluated) Complete course of therapy: 582 (300-1000) Treatment for hepatotoxicity: 323 (250-500) Treatment for non-severe active TB: 2900 (1500-4500) Treatment for severe active TB: 28,692 (10,000-40,000)  Monthly health care costs Base case (range evaluated) NC: 106-1374 (53-2061) HIV: 2061 (1030-3091) Diabetes: 788-2056 (394-3084) ESRD: 3900-5168 (1750-7752) | NR | NR |
| Tasillo 2017 [9] | Published literature (Guo et al 2009; Shepardson et al 2013) | TB disease treatment: 0.76 LTBI treatment: 0.97 LTBI treatment toxicity - no hospitalisation (disutility): 0.25 LTBI treatment toxicity - with hospitalisation (disutility): 0.5 | 2018 | US$ | Medicare fee schedule | IGRA (Quantiferon): From $75 (Texas) to $85 (California) | Published sources (Shepardson 2013) Medicare fee schedule 2020 Holland 2009 US Healthcare cost and utilization project 2020 Marks 2014 | 3HP for 3 months (self-administered): from $394 (Texas) to $451 (California) 3HP for 3 months (observed): from $622 (Texas) to $711 (California) | Published literature (Marks 2014, Castro 2016) | 3HP toxicity not requiring hospitality: From $189 (Texas) to $216 (California) 3HP toxicity requiring hospitality: From $6053 (Texas) to $6926 (California) Outpatient cost of TB treatment: From $3102 (Texas) to $3550 (California) Inpatient cost of TB treatment: From $18,350 (Texas) to $42,356 (California) |
| Jo 2020 [10] | Published sources (Guo 2009, Dion 2002, Dion 2004) | Well: 1.00 LTBI: 1.00 LTBI with treatment without complication: 0.99 LTBI with treatment with complication: 0.85 Active non-MDR TB during treatment and before: 0.80 Active MDR-TB during treatment and before: 0.58 Dead: 0 | 2012 | US$ | Medical insurance reimbursement table in Japan | QFT: $60.6 TSPOT: $60.6 TST: $15.4 CXR: $36.3 | Medical insurance reimbursement table in Japan. Published sources (Holland 2009) | 9H for LBTI: $773.6 3HP for LBTI: $503.5 non-MDR TB for 6 months: $14,879 MDR-TB: $192,921.1 Drug-induce hepatoxicity by LBTI: $11,903 | Ministry of Health, Labor and Welfare. Basic survey on wage structure | Smears, cultures and drug sensitivity tests: $159.7 Physician income per hour: $52.6 Radiologist and lab technician income per hour: $23.0 |
| Linas 2011 [7,8] | Published literature (Guo et al 2009; Dion et al 2004) | Well (no LTBI and no TB): 1 LTBI: 1 LTBI taking treatment without complications: 0.99 LTBI taking treatment with complications: 0.85 TB: 0.8 MDR-TB: 0.58 Dead: 0 | 2020 | Omani riyals (OMR) converted to US dollars (1OMR=USD2.60) | Omani sources | QFT-Plus: 20.5 TST: 23.5 CXR: 26.0 | Omani sources | 6H: 99.7 3HP with DOT: 369.20 4R: 100.8 Treatment of non MDR-TB: 8,038.4 | Kowada 2016 | Treatment of drug-induced hepatotoxicity by LTBI treatment: 4,576 |

Key: 3HP - once-weekly isoniazid- rifapentine for 12 weeks; 4R – 4 months rafimpin; 6H - 6 months isoniazid monotherapy; BCG - Bacillus Calmette–Guérin; CXR – Chest X-ray; CKD – chronic kidney disease; DES – discrete event simulation; IGRA - Interferon Gamma Release Assay; INH – isoniazid; LTBI – Latent tuberculosis infection; MDR – multi drug resistant; NR – Not recorded; QFT – QuantiFERON; RIF – rafimpin; TSPOT - T-Spot TB test; TST – Tuberculin Skin Test

Table B.4: Migrants: Sensitivity and specificity of LTBI tests

| Author/ Trial ID | Source of test accuracy | Sensitivity | | | | | Specificity | | | | |
| --- | --- | --- | --- | --- | --- | --- | --- | --- | --- | --- | --- |
|  |  | *IGRA* | *QFT* | *TSPOT* | *TST* | *CXR* | *IGRA* | *QFT* | *TSPOT* | *TST* | *CXR* |
| Abubakar 2018 [16] | Drawn directly from PREDICT trial. HRs for combinations of tests also provided. Highest HR (and so highest identification of cases) was 0.02003 for TST+(≥6mm or ≥15mm with BCG) in combination with a positive T-SPOT test | NR | **Hazard of developing TB** **per person year** Positive: 0.01039  Negative: 0.00187 | **Hazard of developing TB** **per person year** Positive: 0.01360  Negative: 0.00149 | **Hazard of developing TB** **per person year** TST+(>=5mm): 0.00682  TST-(<5mm):0.00117 TST+(>=6mm or >=15mm with BCG): 0.01121 TST-(<6mm or <15mm with BCG): 0.00157 | NR | NR | NR | NR | NR | NR |
| Al Abri 2020 [19] | Literature | NR | For LTBI: 0.91 For TB: 0.94 | NR | 0.77 | 0.77 | NR | For LTBI: 0.95 For TB: 0.95 | NR | Non-BCG vaccinated: 0.97 BCG vaccinated: 0.59 | 0.6 |
| Auguste 2016 [15] | Meta-analysis of published literature |  | 0.5548 (0.2473-0.8373)  Conditional on -ve TST (LTBI arm): 0.6102 (0.5775-0.6421) Conditional on +ve TST (LTBI arm): 0.4807 (0.0225-0.9724)  Determinate QFT-GIT: 0.97 | 0.7001 (0.3978-0.9242)  Determinate T-SPOT.TB: 0.97 | ≥5mm: 0.9356 (0.7786-0.9977) ≥10mm:0.5915 (0.3584-0.8172)  TST read: 0.94 (0.6-1.0) |  |  | 0.8227 (0.8052-0.8396)  Conditional on +ve TST (no LBTI arm): 0.6102 (0.5775-0.6421) Conditional on -ve TST (no LBTI arm): 0.9746 (0.9555-0.9893) | 0.3992 (0.3439-0.4554) | <5mm: 0.5011 (0.4790-0.5229) <10mm: 0.79297 (0.77809-0.8073) |  |
| Campbell 2017 [12] | Literature | 0.889 | NR | NR | 0.782 | NR | 0.957 | NR | NR | No BCG: 0.974 BCG:0.602 | NR |
| Campbell 2019 [13] | NR | CKD: 0.780 Dialysis: 0.670 | NR | NR | CKD: 0.651 Dialysis: 0.519 | NR | 0.957 Indeterminant=treated as a negative results if it occurred twice in a row; was equally likely to occur in those with and without LTBI Indeterminant CKD: 0.041 Indeterminant dialysis: 0.067 | NR | NR | No BCG: 0.974 BCG: 0.602 | NR |
| Campbell 2019a [14] | Literature | 0.889 | NR | NR | 0.782 | NR | 0.957 | NR | NR | No BCG: 0.974 BCG:0.602 | NR |
| Goodell 2019 [11] | NR |  | USB: 0.85 (0.70-0.97) Non USB: 0.85 (0.70-0.97) |  | USB: 0.83 (0.71-0.87) Non USB: 0.83 (0.71-0.87)  TST positivity prevalence (%) Non-USB: 19.4 (16.1-25.8) USB: 2.4 (0.9-2.6) |  |  | USB: 0.99 (0.98-1.0) Non USB: 0.99 (0.98-1.0) |  | USB: 0.95 (0.82-0.99) Non USB: 0.82 (0.47-0.92) |  |
| Kowada 2016 [17] | QFT sensitivity: Diel 2010 QFT specificity: Diel 2011  TSPOT sensitivity: Diel 2010 TSPOT specificity: Diel 2011  TST sensitivity: Pai 2008 TST specificity: Pai 2008  CXR sensitivity and specificity: Tattevin 1999, Cohen 1996 | NR | 0.84 | 0.89 | 0.77 | 0.70 (active TB) | NR | 0.99 | 0.98 | 0.59 (BCG vaccinated). 0.97 (non-BCG vaccinated) | 0.60 (for active TB) |
| Kowada 2014 [18] | Literature |  | 0.61 (95%CI:0.54-0.67) | 0.65 (95%CI:0.56-0.74) | 0.43 (0.37-0.5) | For active TB 0.7 (0.59-0.82) |  | 0.99 (95%CI:0.98-1.0) | 0.98 (95%CI:0.94-0.99) | For LTBI BCG vaccinated: 0.59 (95%CI: 0.46-0.73) Non-BCG vaccinated: 0.97 (95%CI:0.95-0.99) | For active TB 0.6 (0.52-0.63) |
| Tasillo 2017 [9] | NR | Base case (range evaluated) NC: 79 (0.5-1.0) with HIV: 77 (0.5-1.0) With diabetes: 78 (0.5-1.0) With ESRD: 78 (0.5-1.0) |  |  | Base case (range evaluated) NC: 71 (0.5-1.0) With HIV: 67 (0.5-1.0) With diabetes: 67 (0.5-1.0) With ESRD: 67 (0.5-1.0) |  | Base case (range evaluated) NC: 99 (0.5-1.0) With HIV: 99 (0.5-1.0) With diabetes: 98 (0.5-1.0) With ESRD: 98 (0.5-1.0) |  |  | Base case (range evaluated) NC: 89 (0.5-1.0) With HIV: 87 (0.5-1.0) With diabetes: 87 (0.5-1.0) With ESRD: 87 (0.5-1.0) |  |
| Jo 2020 [10] | Sandgren 2016 | 0.85 | NR | NR | NR | NR | NR | NR | NR | NR | NR |
| Linas 2011 [7,8] | Mazurek 2010, Oxlade 2007, Harada 2008, Detjen 2007, Lee 2006, Palazzo 2008, Ruhwald 2008, Aichelburg 2009, Bartu 2008, Chee 2008, Tsiouris 2006 | 0.83 | NR | NR | 0.89 | NR | 0.99 | NR | NR | Foreign born: 0.92 US born: 0.98 | NR |

Key: 3HP - once-weekly isoniazid- rifapentine for 12 weeks; 4R – 4 months rafimpin; 6H - 6 months isoniazid monotherapy; BCG - Bacillus Calmette–Guérin; CXR – Chest X-ray; DES – discrete event simulation; IGRA - Interferon Gamma Release Assay; INH – isoniazid; LTBI – Latent tuberculosis infection; MDR – multi drug resistant; NR – Not recorded; QFT – QuantiFERON; RIF – rafimpin; TSPOT - T-Spot TB test; TST – Tuberculin Skin Test

Table B.5 Migrants: Treatments for LTBI

| Author/ Trial ID | LTBI treatments considered | Proportion of patients starting treatment | Efficacy of treatment | Percentage maintaining treatment | INH related hepatitis | INH related deaths | Percentage with drug resistant TB | Percentage with multi-drug resistant TB | TB death rates |
| --- | --- | --- | --- | --- | --- | --- | --- | --- | --- |
| Abubakar 2018 [16] | 3HP | 94% (but only offered to under 35s reflecting UK practice) | TB cases with complete CPX: 0.33 TB cases with incomplete CPX: 0.78 | CPX completion rate: 0.85 | 0.0040 (CPX AE) | 0.00002 (death from CPX AE) | NR | NR | 15-44: 0.012 45-64: 0.048 ≥65: 0.176 |
| Al Abri 2020 [19] | INH/RIF | NR | Efficacy: 0.8 | 6H: 0.67 (0.5-0.9) 3HP with DOT: 0.85 (0.5-0.9) 4R: 0.85 (0.5-0.9) | NR | NR | NR | NR | Mortality due to other causes: 0.0028 (0.001-0.01) Mortality of non-MDR TB: 0.07 (0.01-0.2) Mortality of MDR TB: 0.11 (0.04-0.2) |
| Auguste 2016 [15] | INH | Accepting LTBI treatment: 0.9400 (0.50-1.0) | Proportion still infected at end of treatment: 0.345 | TB treatment adherence: 1.0 (not varied) | 0.0040 (0.001-0.010) | 0.00002 (0.00001-0.0001) | NR | NR | NR |
| Campbell 2017 [12] | INH/RIF | Initiate therapy: 0.938 (0.5-1.0) | INH risk reduction: 0.93 RIF risk reduction: 0.8 | Complete INH: 0.616 (0.5-0.7) Complete RIF: 0.814 (0.7-0.9) | NR | 0.00000988 (0-0.0001) | NR | NR | 0.0476 (0-0.08) |
| Campbell 2019 [13] | INH/RIF | 0.938 | Risk reduction: 0.9 | Complete INH: 0.616 (0.561-0.670)  Complete RIF: 0.814 (0.745-0.876) | Adverse event INH: 0.049 (0.044-0.055) Adverse event RIF: 0.021 (0.018-0.025) | 0.00000988 (0-0.00002) | NR | NR | 0.0476 (0.0391-0.0566) |
| Campbell 2019a [14] | INH | 0.735 | Risk reduction: 0.93 | Complete isoniazid: 0.767 (0.690-0.836) | Adverse event INH: 0.089 (0.046-0.143) | Isoniazid: 0.000023 (0.000002-0.00007) | NR | NR | CKD: 0.313 (0.221-0.410) Dialysis: 0.25 (0.14-0.38) |
| Goodell 2019 [11] | INH/RIF | 6h: 0.63 3HP: 0.82 | Efficacy of treatment 9H: 0.92 6H: 0.69 3HP: 0.92 | Proportion of started who complete 6HP: 0.63 (0.54-0.71) 3HP: 0.82 (0.62-1.00) | Mild: 0.0075 Severe: 0.002 | Reported to be included but probability not provided | NR | NR | NR |
| Kowada 2016 [17] | 9H, 3HP | NR | 0.2 | 9H: 0.69 3HP: 0.82 | 9H: 0.027 3HP: 0.004 | NR | NR | 0.036 | NR |
| Kowada 2014 [18] | INH | NR | Efficacy of LTBI treatment: 0.68 | Adherence rate of standard 6-month INH chemoprophylaxis protocol for IGRAs: 0.8 (0-1) Adherence rate of standard 6-month INH chemoprophylaxis protocol for TST: 0.5 (0-1) | Probability of INH induced hepatitis by INH prophylaxis: 0.011 (0.003-0.061) | NR | NR | NR | Increased likelihood of mortality by active TB among HIV+ve pregnant women: 5.2 (95% CI:1.7-15.6) |
| Tasillo 2017 [9] | INH+RIF | Diagnosed who initiate treatment: 0.900 (0.5-1.0) | Reduction in reactivation probability: 0.900 (0.5-1.0) | Treatment completion: 0.783 (0.5-1.0) | NR | NR | NR | NR | 0.05 (0.025-0.075) |
| Jo 2020 [10] | 3HP | 85% | Treatment 93% effective if course completed | NR | Probability of toxicity during LBTI treatment without hospitalisation: 3.2% Probability of toxicity during LBTI treatment with hospitalisation: 0.015 | NR | NR | NR | 5.80% |
| Linas 2011 [7,8] | 9H, 3HP | NR | 0.2 | 9H: 0.69 3HP: 0.82 | 9H: 0.027 3HP: 0.004 | NR | NR | 0.036 | NR |
| Abubakar 2018 [16] | INH | 0.9 | Risk reduction with INH: 3-5mth completed: 30% 6-8mth completed: 60% 9mth completed: 90% | 51% | <35 years: 0.001 >=35 years: 0.01 | 0.01 per case of hepatitis | NR | NR | No medical comorbidities: 0.05 Chronic conditions: 0.06 |

Table B.6 Migrants: model results

| Author/ Trial ID | Total QALYs by strategy | Total costs by strategy | Incremental analyses, e.g. ICER (per QALY gained) | Sensitivity analyses outcomes | Author conclusions | Limitations identified by author |
| --- | --- | --- | --- | --- | --- | --- |
| Abubakar 2018 [16] | TST+(>=5mm): 21.042235 TST+(>=5mm)+IGRA: 21.042235 T-SPOT: 21.042253 TST+(>=5mm)+T-SPOT: 21.042212 TST+(>=6mm or >=15mm with BCG): 21.042244 TST+(>=5mm)+QFT: 21.042205 TST+(>=6mm or >=15mm with BCG)+IGRA: 21.042242 QFT: 21.042230 TST+(>=6mm or >=15mm with BCG)+T-SPOT: 21.042215 TST+(>=6mm or >=15mm with BCG)+QFT: 21.042213 No test: 21.042017 | TST+(>=5mm): £273.62 TST+(>=5mm)+IGRA: £233.06 T-SPOT: £218.03 TST+(>=5mm)+T-SPOT: £211.15 TST+(>=6mm or >=15mm with BCG): £205.43 TST+(>=5mm)+QFT: £201.63 TST+(>=6mm or >=15mm with BCG)+IGRA: £197.80 QFT: £192.88 TST+(>=6mm or >=15mm with BCG)+T-SPOT: £187.27 TST+(>=6mm or >=15mm with BCG)+QFT: £182.32 No test: £27.03 | **INB (vs not test)** TST+(>=5mm): -£242.23 TST+(>=5mm)+IGRA: -£201.67 T-SPOT: -£186.28 TST+(>=5mm)+T-SPOT: -£180.22 TST+(>=6mm or >=15mm with BCG): -£173.86 TST+(>=5mm)+QFT: -£170.84 TST+(>=6mm or >=15mm with BCG)+IGRA: -£166.27 QFT: -£161.58 TST+(>=6mm or >=15mm with BCG)+T-SPOT: -£156.27 TST+(>=6mm or >=15mm with BCG)+QFT: -£151.36 | Testing becomes more cost-effective as baseline incidence of TB increases. If T-SPOT costs were equal to QFT then T-SPOT becomes the optimal testing strategy. If CPX uptake was lower (59.9%) and completion was lower (57%) then QFT becomes the most cost-effective strategy. In a scenario with secondary contacts no testing remained the most cost-effective strategy. | There were modest differences between tests or combinations of tests, in identifying individuals who would go on to develop active TB. However, a two-step approach that combined TST based upon prior BCG vaccination with an IGRA test was most cost-effective | Participants who have left the UK would not have been included in the progression data resulting in an underestimate of the overall progression rate.  Comorbidities were self-reported.  Unable to model the full population effect of better LTBI testing and treatment on the transmission of M. tuberculosis and so results likely underestimate the health benefits and cost savings associated with preventing progression through LTBI testing. The model included a number of simplifying assumptions and some of the sources for input parameter estimates were weak |
| Al Abri 2020 [19] | 1. QFT with 4R: 28.2635 2. QFT with 6H: 28.2074 3. QFT with 3HP: 28.2841 4. TST with 4R: 27.4345 5. TST with 6H: 27.4314 6. TST with 3HP:27.4344 7. CXR: 26.8374 | 1. QFT with 4R: 1420 2. QFT with 6H: 1430 3. QFT with 3HP: 1480 4. TST with 4R: 1872 5. TST with 6H: 1884 6. TST with 3HP: 1951 7. CXR: 3277 | 1. QFT with 4R: 0 2. QFT with 6H: Dominated 3. QFT with 3HP: 2915 4. TST with 4R: Dominated 5. TST with 6H: Dominated 6. TST with 3HP: Dominated 7. CXR: Dominated | Results were sensitive to adherence rates for LTBI treatments QFT-Plus with 3HP was more cost-effective than QFT-Plus with 4R at a WTP level of USD100,000 per QALY gained when adherence rate for 3HP was over 0.80 PSA results showed that QFT-Plus with 3HP was cost effective in 82% of iterations at a WTP level of USD100,000 per QALY gained | IGRA testing followed by 3 months of preventive treatment with 3HP was the most cost-effective intervention | Many parameters were obtained from studies performed in other countries Costs of interventions may vary over time Population assumed to be aged 20 years old |
| Auguste 2016 [15] | TST (≥5mm) +ve then QFT-GIT:19.909 TSPOT.TB: 19.915 QFT-GIT: 19.917 TST (≥5mm): 19.922 TST (≥5mm) -ve then QFT-GIT: 19.923 | TST (≥5mm) +ve then QFT-GIT:300.10 TSPOT.TB: 400.12 QFT-GIT: 291.13 TST (≥5mm): 298.75 TST (≥5mm) -ve then QFT-GIT: 353.47 | TST (≥5mm) +ve then QFT-GIT: Dominated TSPOT.TB: Dominated QFT-GIT: NR TST (≥5mm): 1524 (fs QFT-GIT) TST (≥5mm) -ve then QFT-GIT: 58,720 (vs TST (≥5mm)) | Univariate In the majority of scenarios, as in the base case, the TST (≥5mm)-alone strategy was the most cost-effective A decrease in the prevalence of LTBI, increase in the sensitivity of QFT-GIT and decrease in the sensitivity of TST all led to strategies where QFT-GIT became the most cost-effective PSA At a WTP threshold of £20,000 per QALY gained TST (≥5mm) is cost effective in the highest proportion of simulations (approx. 50%) | For recent arrivals , based on the limited evidence available, TST (≥5mm) was the most cost effective strategy for diagnosing LTBI that progresses to active TB | Evidence available is limited (particularly in terms of inconsistent performance of tests in high- compared with low-incidence TB settings) the prospective assessment of progression to active TB for those at high risk the relative benefits of two- compared with one-step testing with different tests improved classification of people at high and low risk of LTBI |
| Campbell 2017 [12] | TST/INH: 45,026.1 TST/RIF: 45, 025.4 IGRA/INH: 45,030.9 IGRA/RIF: 45,030.1 SEQ/INH: 45,025.8 SEQ/RIF: 45,024.8 No intervention: 45,015.0 | TST/INH: $3,137,675 TST/RIF: $2,194,913 IGRA/INH: $2,946,383 IGRA/RIF: $2,784,661 SEQ/INH: $2,853649 SEQ/RIF: $2,756,316 No intervention: $2,616,436 | TST/RIF: 312,952 (SW quadrant) IGRA/INH: Dominant IGRA/RIF: Dominant SEQ/INH: 1,064,235 (SW quadrant) SEQ/RIF: 308,919 (SW quadrant) No intervention: 51,581 | Univariate Extending the time horizon had the most significant impact in favour of IGRA/RIF (NMB increased by over $1.2m if extended to 50 years) Reducing the effectiveness of a full course of RIF to 50% had the most significant impact against IGRA/RIF (NMB reduced by over $600,000) IGRA/RIF robust to other univariate SA (no other change resulted in base case having a higher NMB)  PSA Migrants under surveillance: IGRA/RIF had a probability of being cost effective 64.9% at a WTP of $100,000 per QALY gained | Screening migrants under surveillance with IGRA and treating with rifampin is cost saving but does not impact TB incidence. Cost of universal LTBI screening and treatment is cost-prohibitive. | • Remote infections directly linked to intervention (if few TB cases due to reactivation then few TB cases due to remote transmission) • Assumption that all migrants who report to clinic are offered LBTI screening (unlikely to reflect reality) • LBTI test may not be reliable in people with previous diagnosis of TB (due to a lasting immune response) • Assumed drop outs were random (some pts will never be offered therapy due to age, co-morbid conditions or feasibility) • Comorbid conditions that may increase risk of TB were not considered |
| Campbell 2019 [13] | Age <60 years [Results also available for ≥60 years] Low TB incidence BC: 2.79916 IGRA/INH: 2.79946 TST/INH: 2.79932  Moderate TB incidence BC: 2.77204 IGRA/INH: 2.77393 TST/INH: 2.77337  High TB incidence BC: 2.79032 IGRA/INH: 2.79260 TST/INH: 2.79189  Very high TB incidence BC: 2.78068 IGRA/INH: 2.78464 TST/INH: 2.78347 | Age <60 years [Results also available for ≥60 years] Low TB incidence BC: $93.22 IGRA/INH: $148.22 TST/INH: $203.50  Moderate TB incidence BC: $558.43 IGRA/INH: $555.95 TST/INH: $663.30  High TB incidence BC: $665.25 IGRA/INH: $656.54 TST/INH: $759.94  Very high TB incidence BC: 2$1,128.83 IGRA/INH: $1,063.92 TST/INH: $1,165.36 | Age <60 years [Results also available for ≥60 years] Versus BC Low TB incidence IGRA/INH: $183,187 TST/INH: $686,536  Moderate TB incidence IGRA/INH: Dominant TST/INH: $78,578  High TB incidence IGRA/INH: Dominant TST/INH: $60,443  Very high TB incidence IGRA/INH: Dominant TST/INH: $13,112 | PSA (Aged <60 years, IGRA/RIF [most CE strategy] number of iterations CE at WTP threshold of $100,000 Low incidence: not likely to be cost effective Moderate incidence: 79.4% High incidence: 82.3% Very high incidence: 90.5% | Pre-immigration IGRA screening and post-arrival RIF treatment was preferred in deterministic analysis. Our analysis provides evidence of the cost-effectiveness of pre-immigration LTBI screening for migrants to low-incidence countries. | • For some persons the risk for SAEs may outweigh the benefits of treatment • Social factors and concurrent conditions may increase the risk for reactivation of LTBI • reactivation rate of LBTI sourced from literature but many studies were based on TB incidence in those who were positive by TST and therefore the predictive value of the TST caused underestimation of true reactivation rates • Three months or once-weekly isoniazid and rifapentine not considered (as not universally available) but may yield similar results to rifampin treatment • Costs incurred by persons experiencing the intervention not considered |
| Campbell 2019a [14] | QALYs per 1000 persons • Most cost effective strategy • Low incidence: SEQ/RIF 13,761.30 • Moderate incidence: IGRA/RIF 13,736.66 • High incidence: IGRA/RIF 13,705.48 Very high incidence: IGRA/RIF 13,671.50 | Costs per 1000 persons • Most cost effective strategy • Low incidence: SEQ/RIF $60,996 • Moderate incidence: IGRA/RIF $129,036 • High incidence: IGRA/RIF $199,878 Very high incidence: IGRA/RIF $268,840 | Costs per 1000 persons • Most cost effective strategy • Low incidence: SEQ/RIF $191,889 • Moderate incidence: IGRA/RIF $43,343 • High incidence: IGRA/RIF $26,350 Very high incidence: IGRA/RIF $16,291 | PSA (WTP threshold of $50,000 [$100,000]) • Most cost effective strategy • Low incidence: SEQ/RIF 49.1% [50.7%] • Moderate incidence: IGRA/RIF 57.5% [59.8%] • High incidence: IGRA/RIF 68.2% [70.6%] Very high incidence: IGRA/RIF 73.2% [75.2%] | LTBI screening should be considered in non-Canadian born residents initiating dialysis therapy and those with late stage CKD who are older (>60 years) | Limited generalisability to different epidemiologic settings  Clinical discretion in prescribing LTBI treatments (namely, rifapentine/isoniazid, rifampin [4m], rifapentine/isoniazid [3m]) not considered due to difficulty in modelling the complex risk-benefit considerations involved Only a healthcare perspective taken Risks that potentially affect longitudinal TB risk that were not taken into account include abnormal CSR findings, socioeconomic status and substance misuse |
| Goodell 2019 [11] | NR | US$ (billion) Base case: 5.6 (5.4-6.6) Non-US born (x2): 7.8 (7.1-8.3) Medical risk factor (x2): 7.1 (6.6-7.7) Non-US born+medical risk factor (x2): 8.6 (7.9-9.2) Universal (x2): 11 (10-12) | California only US$ (thousand) Base case: NR Non-US born (x2): 167 (116-347) Medical risk factor (x2): 258 (183-780) Non-US born+medical risk factor (x2): 226 (159-455) Universal (x2): 408 (314-946) | None of the main strategies were cost-effective at a WTP threshold of $100,000; however, restricting to single lifetime testing generates an ICER of $80,0000 per QALY gained (doubles TTT using QFT tests and 3HP/DOT treatment in the non-USB population) | Substantial gains can be made in TB control in coming years by scaling-up current testing and treatment in non-US born and those with medical risks | 3HP was modelled as DOT Economies of scale not modelled A non-geographic transmission model was used, instead modelled non-random mixing by nativity and race/ethnicity (important determinants) Use of two phases of reactivation rates (recent vs remote transmission) is a simplification National rather than Californian-specific estimates of LTBI prevalence were used but were adjusted to better reflect Californian demographics Poorly accounts for the undocumented and unhoused populations (high risk populations) Changes in immigration patterns not modelled |
| Kowada 2016 [17] | QFT with 3HP: 25.95660 QFT with 9H: 25.95499 TSPOT with 3HP: 25.94438 TSPOT with 9H: 25.94288 TST with 3HP: 25.34343 TST with 9H: 25.34959 CXR: 24.62917 | QFT with 3HP: $674.8 QFT with 9H: $688.1 TSPOT with 3HP: $829.6 TSPOT with 9H: $847.1 TST with 3HP: $5795.9 TST with 9H: $5941.3 CXR: $13,666.8 | QFT with 3HP is the dominant strategy | QFT remains a dominant strategy across range of sensitivities and specificities considered (although treatment with 3H no longer dominates). QFT with 3HP treatment had a 75%-79% of being the most cost effective treatment at WTP thresholds up to $50,000 per QALY | Entry LTBI screening using QFT and 3HP (if needed) is recommended on the basis of cost effectiveness for long-term expatriates from low to high incidence countries | Little data on LTBI and TB prevalence for this population No LTBI test and LTBI treatment for MDR-TB Parameter inputs were obtained from multiple countries No data of periodic TB screening for expatriates Social and economic context of the expatriates not considered |
| Kowada 2014 [18] | Non-BCG vaccinated cohort during pregnancy TST/QFT: 20.68700 TST/T-SPOT: 20.68667 TST: 20.68126 QFT:20.67790 T-SPOT: 20.67707  BCG vaccinated cohort during pregnancy TST/QFT: 20.68663 TST/T-SPOT: 20.68595 QFT: 20.67790 T-SPOT: 20.67707 TST: 20.61889  Non-BCG vaccinated cohort in postpartum period TST/QFT: 20.64669 TST/T-SPOT: 20.64622 TST: 20.64021 QFT: 20.63565 T-SPOT: 20.63408  BCG vaccinated cohort in postpartum period TST/QFT: 20.64641 TST/TSPOT: 20.64568 QFT: 20.63565 T-SPOT: 20.63408 TST: 20.59238 | Non-BCG vaccinated cohort during pregnancy TST/QFT: 5754.29 TST/T-SPOT: 5776.08 TST: 6039.64 QFT: 6200.78 T-SPOT: 6292.33  BCG vaccinated cohort during pregnancy TST/QFT: 5793.30 TST/T-SPOT: 5827.71 QFT: 6200.78 T-SPOT: 6292.33 TST: 7718.16  Non-BCG vaccinated cohort in postpartum period TST/QFT: 6405.75 TST/T-SPOT: 6437.81 TST: 6761.41 QFT: 7047.88 T-SPOT: 7153.29  BCG vaccinated cohort in postpartum period TST/QFT: 6434.97 TST/TSPOT: 6076.35 QFT: 7047.88 T-SPOT: 7153.29 TST: 8034.15 | "Non-BCG vaccinated cohort during pregnancy TST/QFT: -  TST/T-SPOT: Dominated TST: Dominated QFT: Dominated T-SPOT: Dominated  BCG vaccinated cohort during pregnancy TST/QFT: - TST/T-SPOT: Dominated QFT: Dominated T-SPOT: Dominated TST: Dominated  Non-BCG vaccinated cohort in postpartum period TST/QFT: - TST/T-SPOT: Dominated TST: Dominated QFT: Dominated T-SPOT: Dominated  BCG vaccinated cohort in postpartum period TST/QFT: - TST/TSPOT: Dominated QFT: Dominated T-SPOT: Dominated TST: Dominated | Results were sensitive to the sensitivity of - T-SPOT and the sensitivity of QFT in non-BCG vaccinated immigrants - T-SPOT in BCG vaccinated immigrants | Using an IGRA for TB screening of high-risk HIV +ve pregnant women in low TB incidence countries is recommended on the basis of cost effectiveness | Estimates of each of the variables in the model were obtained from studies conducted in numerous countries The sensitivity and specificity estimates for IGRAs in HIV+ve pregnant women were obtained from meta-analyses of published literature and assumptions gut little is known on effect of pregnancy on sensitivity and specificity of IGRAs and TST Prevention of TB spread by pregnant women is a more urgent problem in developing countries than in developed countries Routine use of chest radiography in the absence of clear symptoms of active TB may be unwarranted and raise ethical considerations Women may be less likely than men to submit good-quality sputum The use of chemoprophylaxis for pregnant women is controversial There is little data of TB incidence and LTBI prevalence in HIV +ve pregnant women There is no data of TB risk in pregnancy trimester Different countries have different policies and resources for TB screening - generalisability of results unclear Costs of the side effects of MDR-TB therapy was not included in the model Use of chemoprophylaxis for pregnant women is controversial |
| Tasillo 2017 [9] | Incremental QALYs versus no testing NC Confirm positive: 0.0013 TST: 0.0004 IGRA: 0.0006 Confirm negative: 0.003  Diabetes Confirm positive: 0.0009 TST: 0.0002 IGRA: 0.0005 Confirm negative: 0.0002  HIV Confirm positive: 0.0032 TST: 0.0010 IGRA: 0.0017 Confirm negative: 0.0008  ESRD Confirm positive: 0.0003 TST: 0.0001 IGRA: 0.0002 Confirm negative: 0.0001 | Incremental cost per person versus no testing NC Confirm positive: 47 TST: 30 IGRA: 50 Confirm negative: 42  Diabetes Confirm positive: 47 TST: 35 IGRA: 54 Confirm negative: 47  HIV Confirm positive: 58 TST: 39 IGRA: 55 Confirm negative: 50  ESRD Confirm positive: 852 TST: 267 IGRA: 492 Confirm negative: 237 | ICER NC Confirm positive: 35,000 TST: Dominated IGRA: 83,000 Confirm negative: 147,000  Diabetes Confirm positive: 53,000 TST: Dominated IGRA: 35,000 Confirm negative: 63,000  HIV Confirm positive: 18,000 TST: Dominated IGRA: 35,000 Confirm negative: 63,000  ESRD Confirm positive: 2,730,000 TST: Dominated IGRA: 2,933,000 Confirm negative: 3,546,000 | Deterministic SA: in general, the base case conclusions were robust to changes in core model parameters PSA - strategy preferred most frequently  - NC: IGRA (29% of simulations)  - Diabetes: Confirmed positive (35.9% of simulations)  - HIV: Confirmed positive (39.5% of simulations)  - EASD: Confirmed negative (63% of simulations) | Testing for and treating LTBI is likely to be cost-effective except among those with ESRD in whom competing risks of death limit benefits. Strategies including IGRA fell below a WTP threshold of $100,000 for those with NC, diabetes and HIV | • Model only considered 1 generation of TB transmission (therefore spread by dialysis patients not considered) • Uncertainty around the prevalence of LTBI and the test characteristics of TST and IGRA due to absence of a reference standard for LTBI diagnosis • Rate of TB reactivation over decades of remaining life is uncertain • Societal perspective not considered |
| Jo 2020 [10] | Total incremental QALYs with TTT over 30 years ranged from 1,285 in New York to 5,903 in California | Total incremental costs with TTT over 30 years ranged from $243,378,612 in Texas to $765,111,089 in California | ICER per QALY gained versus no TTT ranged from $81,790 in Texas to $174,152 in New York | Results were most sensitive to the State specific trend in TB incidence, cost of IGRA and impact of 3HP toxicity. In 67% of PSA iterations in all states the ICER was below $150,000 per QALY gained with TTT | Cost-effectiveness of TTT for LTBI differs across states but is most cost-effective in people with HIV followed by migrants | Lack of evidence on key model parameters (LTBI prevalence, reactivation rates and disutility in taking 3HP). Only a 30 year time horizon rather than lifetime. Only include health care system costs of testing and treating (i.e. exclude programme implementation costs and additional testing of people with abnormal radiographs). Identification of active TB not included. Multidrug resistant TB, different diagnostic algorithms, different treatment options, and risk stratification were not included in the model. |
| Linas 2011 [7,8] | Discounted quality adjusted life months presented in table **Adults** No screening: 308.26 TST: 308.31 IGRA: 308.31 **Children** No screening: 360.16 TST: 360.34 IGRA: 360.36 | **Adults** No screening: $122,560 TST: $122,700 IGRA: $122,700 **Children** No screening: $131,940 TST: $132,010 IGRA: $132,020 | **Adults** IGRA dominates TST with IGRA having an ICER per QALY gained of $35,200 compared to no testing **Children** IGRA has an ICER per QALY gained of $74,800 per QALY gained compared to no testing. TST is stated to be dominated by IGRA but TST has a higher cost and higher QALYs so this should not be the case and appears to be an error in the publication | ICERs were sensitive to rates of reactivation (lower rates resulted in higher ICERs) and higher costs of IGRA testing (above $64). Other parameters were not found to significantly alter ICERs | LBTI screening would be cost effective in high risk groups in the USA. IGRA screening is more cost effective than TST screening | True activation rate of TB unknown, no gold standard test for TB, only direct medical costs included |

Key: 3HP - once-weekly isoniazid- rifapentine for 12 weeks; 4R – 4 months rafimpin; 6H - 6 months isoniazid monotherapy; BCG - Bacillus Calmette–Guérin; CPX – chemoprophylaxis; CXR – Chest X-ray; IGRA - Interferon Gamma Release Assay; INH – isoniazid; LTBI – Latent tuberculosis infection; MDR – multi drug resistant; NR – Not recorded; QFT – QuantiFERON; RIF – rafimpin; TSPOT - T-Spot TB test; TST – Tuberculin Skin Test; ICER – Incremental Cost-Effectiveness Ratios; PSA – Probabilistic Sensitivity Analysis; QALY – Quality Adjusted Life Year;

# APPENDIX C

# Included studies full extraction tables: People with HIV

Table C.1 People with HIV: Population information and tests considered

| Author/ Trial ID | Country/region | Description of population | Tests assessed | Prevalence rate of LTBI | BCG vaccination rate | TB activation rate | Secondary transmission rate |
| --- | --- | --- | --- | --- | --- | --- | --- |
| Capocci 2020 [26] | UK (one HIV clinic in London) | All HIV patients | TST, IGRA, CXR | BA: 0.17% MI: 0.09% LI: 0.04% | 0.85 | TST+ve alone: 2%  IGRA+TST+ve: 10% TST-ve: 0.2% | 20% (scenario analysis) |
| Capocci 2016 [24] | UK | HIV population | TST, IGRA | NR | NR | NR | NR |
| Capocci 2016a [25] | UK | Recent arrivals | TST, IGRA, CXR | Base case (range for SA) 0.0237 (0.0150-0.0345) | NR | Proportion still infected post-LTBI treatment: 0.345 Average number of secondary cases from one index case: 0.2 (0.1-0.3) Average delay from infection to activation (secondary cases): 2.88 | NR |
| Capocci 2015 [23] | UK (one HIV clinic in London) | All HIV patients | Testing all patients  IGRA for all  NICE Guideline  IGRA+TST only if CD4<500  BHIVA Guideline  IGRA test only if  from sSA: ART<2 years from MI: CD4<400 and ART<2 years from low TB incidence country:CD4<350 and ART<6months | From clinic database  Sub-Saharan Africa: 13% Middle incidence TB countries: 10% Low incidence TB countries: 3% | NR | NR | NR |
| Capocci 2014 [22] | UK | All HIV patients | NICE Guideline (not in abstract but taken from guideline)  IGRA+TST only if CD4<500  BHIVA Guideline (not in abstract but taken from guideline)  IGRA test only if  from sSA: ART<2 years from MI: CD4<400 and ART<2 years from low TB incidence country:CD4<350 and ART<6months | Patients identified with LTBI  Sub-Saharan Africa: 15% Middle incidence TB countries: 6% Low incidence TB countries: 3% | NR | TST+ve/IGRA-ve: 2%  IGRA+TST+ve: 10% | NR |
| Capocci 2012 [21] | UK | All HIV patients | NICE Guideline (not in abstract but taken from guideline)  IGRA+TST only if CD4<500  BHIVA Guideline (not in abstract but taken from guideline)  IGRA test only if  from sSA: ART<2 years from MI: CD4<400 and ART<2 years from low TB incidence country:CD4<350 and ART<6months | Sub-Saharan Africa: 20% Middle incidence TB countries: 8% Low incidence TB countries: 2% | NR | NR | NR |
| Kowada 2013 [27] | Japan | Prisoners | NR | Probability of having HIV: 0.08 (0.04-0.4) | NR | Increased likelihood of progression from LTBI to active TB in advanced, untreated HIV infection: 9.9 (8.7-11) | NR |
| Kowada 2014 [18] | Japan | Pregnant HIV +ve women | TST QFT T-SPOT TST/QFT TST/T-SPOT CXR | Incidence of TB among 20 year old HIV+ve pregnant women in postpartum period: 0.0003 (0.00002-0.00065) TB risk during pregnancy: 1.29 (95% cI: 0.82-2.03) TB risk in postpartum period: 1.95 (1.24-3.07) | NR | Increased likelihood of progression from LTBI to active TB among HIV positive pregnant women: 4.5 (95%CI: 1.1-18.0) | NR |

Key: 3HP - once-weekly isoniazid- rifapentine for 12 weeks; 4R – 4 months rafimpin; 6H - 6 months isoniazid monotherapy; BCG - Bacillus Calmette–Guérin; CXR – Chest X-ray; IGRA - Interferon Gamma Release Assay; INH – isoniazid; LTBI – Latent tuberculosis infection; MDR – multi drug resistant; NR – Not recorded; QFT – QuantiFERON; RIF – rafimpin; TSPOT - T-Spot TB test; TST – Tuberculin Skin Test

Table C.2 People with HIV: Modelling methods

| Author/ Trial ID | Model type | Time horizon | Health states | Perspective | Discounting |
| --- | --- | --- | --- | --- | --- |
| Capocci 2020 [26] | Poorly described but appears to be a decision tree/payoff model | Lifetime | Decision tree nodes  Agree or decline testing Old TB or Test +ve/-ve/ and treatment Progression to TB from +ve treated and negative Reactivation of old TB | NHS | 3.5% pa (costs and benefits) |
| Capocci 2016 [24] | Not clear that a model has been used | Not reported | NR | NHS | Not reported |
| Capocci 2016a [25] | Not clear that a model has been used | NR | NR | NHS | Not reported |
| Capocci 2015 [23] | Poorly described but appears to be a decision tree/payoff model | Lifetime | Decision tree nodes  Agree or decline testing Test +ve/-ve/ and treatment | NHS | 3.5% pa (costs and benefits) |
| Capocci 2014 [22] | Poorly described but appears to be a decision tree/payoff model | Lifetime | NR | NR | NR |
| Capocci 2012 [21] | Poorly described but appears to be a decision tree/payoff model | NR | NR | NR | NR |
| Kowada 2013 [27] | Decision tree followed by a Markov model. | Lifetime (length not reported although it is stated that average time spent living in a nursing home is 4 years) | Decision tree Test result  CXR if +ve  LTBI treatment adherence w/wout complication   Markov model Healthy (no TB and no LTBI) LTBI TB (drug sensitive or MDR) Dead | Societal | 3% for costs and benefits |
| Kowada 2014 [18] | Decision tree followed by a Markov model. | 30 years | Decision tree Test result  CXR if +ve  LTBI treatment adherence w/wout complication   Markov model Healthy (no TB and no LTBI) LTBI TB (drug sensitive or MDR) Dead | Public health payer | 3% for costs and benefits |

Key: 3HP - once-weekly isoniazid- rifapentine for 12 weeks; 4R – 4 months rafimpin; 6H - 6 months isoniazid monotherapy; BCG - Bacillus Calmette–Guérin; CXR – Chest X-ray; DES – discrete event simulation; IGRA - Interferon Gamma Release Assay; INH – isoniazid; LTBI – Latent tuberculosis infection; MDR – multi drug resistant; NR – Not recorded; QFT – QuantiFERON; RIF – rafimpin; TSPOT - T-Spot TB test; TST – Tuberculin Skin Test

Table C.3 People with HIV: Cost and utility parameter values

| Author/ Trial ID | Sources of utility values | Utility values used in model (mean (SD)) | Cost year | Currency | Source of test costs | Test costs in model | Sources of treatment costs | Treatment costs in model | Sources of other costs | Other costs in model |
| --- | --- | --- | --- | --- | --- | --- | --- | --- | --- | --- |
| Capocci 2020 [26] | NICE TB Guideline | **QALY losses**  Active TB: 0.676 Treatment of symptomatic, smear negative, culture positive TB: 0.2 Treatment of LTBI: 0.007 | 2018/19 | GBP | Local charges and published studies | TST: £18.51 IGRA: £53.34 CXR: £38.10 | Local charges and published studies | Cost of LTBI: £666.04 | Local charges and published studies | Sputum induction: £100 Active TB: £9674.21 Asymptomatic TB: £1,247.62 |
| Capocci 2016 [24] | Published literature (sources not provided) | Not reported | NR | UK£ | Published literature (sources not provided) | Not reported | Published literature (sources not provided) | Not reported | Published literature (sources not provided) | Not reported |
| Capocci 2016a [25] | NR | NR | NR | NR | NR | NR | NR | NR | NR | NR |
| Capocci 2015 [23] | NICE TB Guideline | **QALY losses**  Active TB: 0.676 Treatment of LTBI: 0.007 | 2011/12 | Euro | Local charges and published studies | TST: €19.37 IGRA: €72 | Local charges and published studies | 6 months isoniazid: €943.80 | Published literature (Pooran 2010) | Treatment for active TB: €9143.60 |
| Capocci 2014 [22] | NR | NR | NR | GBP | BNF | TST: £16 IGRA: £60 CXR: £50 Sputum: £42 | BNF | £786 | BNF | Treatment for active TB: £7619 |
| Capocci 2012 [21] | NR | **QALY losses**  Active TB: 0.676 Treatment of LTBI: 0.007 | NR | GBP | NR | NR | NR | NR | NR | NR |
| Kowada 2013 [27] | Guo 2009 Dion 2004 | Well: 1 LTBI taking LTBI treatment without complication: 0.95 LTBI taking LTBI treatment with liver dysfunction: 0.85 DS-TB during treatment and before: 0.8 MDR-TB during treatment and before: 0.58 Dead: 0 | 2014 | US$ | Medical insurance re-imbursement data WHO guidelines Wage structure survey | QFT: 59.5 (22.5-97.1) T-SPOT: 59.5 (22.5-97.1) TST: 15.1 (10.9-31.5) CXR: 35.6 (17.8-71.2) | Literature | 9H chemoprophylaxis: 1219.3 (390.2-1817.2) Treatment of drug-induced hepatitis by chemoprophylaxis: 11,689 (5845-23,378) Treatment of DS-TB for 6 months: 14,612 (7306-29,224) Treatment of MDR-TB: 1,89,457 (94,729-378,914) | Literature | Smears, cultures and drug sensitivity test of sputum examination: 156.8 (78.4-313.6) |
| Kowada 2014 [18] | Guo 2009 Resch 2006 | Non-LTBI and non-TB in HIV +ve pregnant state: 1 LTBI taking no chemoprophylaxis in HIV positive pregnant state: 1 LTBI taking chemoprophylaxis without complication in HIV positive pregnant state: 0.99 LTBI taking chemoprophylaxis with liver dysfunction in HIV positive pregnant state: 0.85 Active non MDR-TB during treatment and before in HIV +ve pregnant state: 0.80 Active MDR-TB during treatment and before in HIV positive pregnant state: 0.58 Dead: 0 | 2012 | US$ | Medical insurance reimbursement table | QFT: 60.6 (30.3-120.2) T-SPOT: 60.6 (30.3-120.2) TST: 15.4 (7.7-30.8) CXR: 36.3 (18.2-61.6) | Kowada 2008 Resch 2006 | INH chemoprophylaxis for 6 months: 515.7 (257.9-1,035.4) Treatment of INH-induced hepatitis by chemoprophylaxis: 11,903 (5951.5-23,806) Treatment of non MDR-TB for 6 months: 14,879 (7440-29578) Treatment of MDR-TB: 192,921 (96,461-385842) | Medical insurance reimbursement table | Smears and cultures of sputum examination: 69.9 (35.0-139.8) |

Key: 3HP - once-weekly isoniazid- rifapentine for 12 weeks; 4R – 4 months rafimpin; 6H - 6 months isoniazid monotherapy; BCG - Bacillus Calmette–Guérin; CXR – Chest X-ray; IGRA - Interferon Gamma Release Assay; INH – isoniazid; LTBI – Latent tuberculosis infection; MDR – multi drug resistant; NR – Not recorded; QFT – QuantiFERON; RIF – rafimpin; TSPOT - T-Spot TB test; TST – Tuberculin Skin Test

Table C.4 People with HIV: Sensitivity and specificity of LTBI tests

| Author/ Trial ID | Source of test accuracy | Sensitivity | | | | | Specificity | | | | |
| --- | --- | --- | --- | --- | --- | --- | --- | --- | --- | --- | --- |
|  |  | *IGRA* | *QFT* | *TSPOT* | *TST* | *CXR* | *IGRA* | *QFT* | *TSPOT* | *TST* | *CXR* |
| Capocci 2020 [26] | NR | NR | NR | NR | NR | NR | NR | NR | NR | NR | NR |
| Capocci 2016 [24] | NR | NR | NR | NR | NR | NR | NR | NR | NR | NR | NR |
| Capocci 2016a [25] | NR | NR | NR | NR | NR | NR | NR | NR | NR | NR | NR |
| Capocci 2015 [23] | Aichelburg 2009 | 91% (3% indeterminate rate) | NR | NR | NR | NR | NR | NR | NR | NR | NR |
| Capocci 2014 [22] | NR | NR | NR | NR | NR | NR | NR | NR | NR | NR | NR |
| Capocci 2012 [21] | NR | NR | NR | NR | NR | NR | NR | NR | NR | NR | NR |
| Kowada 2013 [27] | QFT sensitivity: Diel 2010 QFT specificity: Diel 2011  TSPOT sensitivity: Diel 2010 TSPOT specificity: Diel 2011  TST sensitivity: Pai 2008 TST specificity: Pai 2008  CXR sensitivity and specificity: Tattevin 1999, Cohen 1996 |  | LTBI:0.61 (95% CI: 0.54-0.67) | LTBI: 0.65 (95% CI: 0.56-0.74) | For LTBI: 0.43 (0.37-0.5) | Active TB: 0.70 (0.59-0.82) |  | LTBI: 0.99 (95% CI: 0.98-1.00) Active TB: 0.79 (95% CI: 0.75-0.82) | LTBI: 0.98 (95% CI: 0.94-0.99) Active TB: 0.59 (95% CI: 0.56-0.62) | **LTBI** BCG vaccinated: 0.59 (95% CI: 0.46-0.73) Non-BCG vaccinated: 0.97 (95% CI: 0.95-0.99) | Active TB: 0.60 (0./52-0.63) |
| Kowada 2014 [18] | Literature |  | 0.61 (95%CI:0.54-0.67) | 0.65 (95%CI:0.56-0.74) | 0.43 (0.37-0.5) | For active TB 0.7 (0.59-0.82) |  | 0.99 (95%CI:0.98-1.0) | 0.98 (95%CI:0.94-0.99) | For LTBI BCG vaccinated: 0.59 (95%CI: 0.46-0.73) Non-BCG vaccinated: 0.97 (95%CI:0.95-0.99) | For active TB 0.6 (0.52-0.63) |

Key: 3HP - once-weekly isoniazid- rifapentine for 12 weeks; 4R – 4 months rafimpin; 6H - 6 months isoniazid monotherapy; BCG - Bacillus Calmette–Guérin; CXR – Chest X-ray; IGRA - Interferon Gamma Release Assay; INH – isoniazid; LTBI – Latent tuberculosis infection; MDR – multi drug resistant; NR – Not recorded; QFT – QuantiFERON; RIF – rafimpin; TSPOT - T-Spot TB test; TST – Tuberculin Skin Test

Table C.5 People with HIV: Treatments for LTBI

| Author/ Trial ID | LTBI treatments considered | Proportion of patients starting treatment | Efficacy of treatment | Percentage maintaining treatment | INH related hepatitis | INH related deaths | Percentage with drug resistant TB | Percentage with mulit-drug resistant TB | TB death rates |
| --- | --- | --- | --- | --- | --- | --- | --- | --- | --- |
| Capocci 2020 [26] | INH/RIF | 50% | Efficacy: 0.62 | NR | NR | NR | NR | NR | NR |
| Capocci 2016 [24] | NR | NR | NR | NR | NR | NR | NR | NR | NR |
| Capocci 2016a [25] | INH/RIF | NR | NR | NR | NR | NR | NR | NR | NR |
| Capocci 2015 [23] | INH/RIF | 87% | Efficacy: 0.62 | NR | NR | NR | NR | NR | NR |
| Capocci 2014 [22] | INH/RIF | NR | NR | NR | NR | NR | NR | NR | NR |
| Capocci 2012 [21] | INH/RIF | 100% | Efficacy: 0.62 | NR | NR | NR | NR | NR | NR |
| Kowada 2013 [27] | 9H | NR | Efficacy: 0.8 | Adherence rate of 9H chemoprophylaxis: 0.365 (0-1) | Probability of drug-related hepatotoxicity by 9H chemoprophylaxis: 0.021 (0.01-0.04) | NR | 0.60% | NR | TB mortality: 0.366 (0.2-0.5) |
| Kowada 2014 [18] | INH | NR | Efficacy of LTBI treatment: 0.68 | Adherence rate of standard 6-month INH chemoprophylaxis protocol for IGRAs: 0.8 (0-1) Adherence rate of standard 6-month INH chemoprophylaxis protocol for TST: 0.5 (0-1) | Probability of INH induced hepatitis by INH prophylaxis: 0.011 (0.003-0.061) | NR | NR | NR | Increased likelihood of mortality by active TB among HIV+ve pregnant women: 5.2 (95% CI:1.7-15.6) |

Key: 3HP - once-weekly isoniazid- rifapentine for 12 weeks; 4R – 4 months rafimpin; 6H - 6 months isoniazid monotherapy; BCG - Bacillus Calmette–Guérin; CXR – Chest X-ray; IGRA - Interferon Gamma Release Assay; INH – isoniazid; LTBI – Latent tuberculosis infection; MDR – multi drug resistant; NR – Not recorded; QFT – QuantiFERON; RIF – rafimpin; TSPOT - T-Spot TB test; TST – Tuberculin Skin Test

Table C.6 People with HIV: Results

| Author/ Trial ID | Total QALYs by strategy | Total costs by strategy | Incremental analyses, e.g. ICER (per QALY gained) | Sensitivity analyses outcomes | Author conclusions | Limitations identified by author |
| --- | --- | --- | --- | --- | --- | --- |
| Capocci 2020 [26] | QALYs gained per 10,000 over no testing TST in BA: 2.1 TST in BA and MI: 2.4  TST+CXR BA: 3.7 IGRA in BA: 3.8 TST in all: 2.9 TST+CXR in BA and MI: 4.5 IGRA+CXR in BA: 5.4 IGRA in BA and MI: 5.1 CXR in all: 3.0  IGRA in all: 5.7 IGRA+TST in all: 5.9 IGRA+CXR in all: 8.7 IGRA+TST+CXR in all: 8.9 | Costs per 10,000 No testing: £889,527 TST in BA: £922,744 TST in BA and MI: £933,733 TST+CXR BA: £970,468 IGRA in BA: £997,509 TST in all: £1,000,540 TST+CXR in BA and MI: £1,015,540 IGRA+CXR in BA: £1,045,234 IGRA in BA and MI: £1,056,145 CXR in all: £1,095,328  IGRA in all: £1,303,929 IGRA+TST in all: £1,475,296 IGRA+CXR in all: £1,559,576 IGRA+TST+CXR in all: £1,730,943 | ICER vs no testing TST in BA: £15,868 TST in BA and MI: £18,221 TST+CXR BA: £21,900 IGRA in BA: £28,074 TST in all: £38,801 TST+CXR in BA and MI: £28,059 IGRA+CXR in BA: £28,575 IGRA in BA and MI: £32,660 CXR in all: £85,768 IGRA in all: £72,397 IGRA+TST in all: £99,566 IGRA+CXR in all: £76,975 IGRA+TST+CXR in all: £94,926 | Testing in BA only was most cost effective strategy with choice of test being dependent on test costs (IGRA had to be below £22). Doubling of treating active TB costs resulted in TST in BA+MI being cost effective. As uptake and efficacy of LBTI increased to over 80% then IGRA alone became cost effective at £30,000/QALY. If onward transmission was included then testing all BA and MI with IGRA and CXR becomes cost effective at £29,818. PSA showed no testing was most cost-effective up to £20,000 WTP in 100% of iterations with TST in BA most likely to be cost effective for a WTP up to £50,000. | Testing all HIV patients in the UK with high uptake ART is unlikely to be cost-effective regardless of testing strategy employed. Testing of migrants from high or middle income countries may be cost effective | Less than 50% of those approached agreed to take part in the observational study Difficulty of knowing actual risk of developing TB from LTBI Small numbers in study Transmission not included in base case but when included actually reduced cost effectiveness of testing |
| Capocci 2016 [24] | NR | Costs per 10,000 No testing: £501,345 TST in sSA: £550,389 TST in sSA and MI: £562,526  IGRA in sSA: £612,777 IGRA in sSA and MI: £666,688 TST in all: £628,807 IGRA in all: £851,577 TST and IGRA in all: £1,014,029 IGRA and CXR in all: £1,181,280 | ICER per QALY gained over no testing TST in sSA: £22,410 TST in sSA and MI: £24,121 IGRA in sSA: £27,710 IGRA in sSA and MI: £30,999 TST in all: £48,166 IGRA in all: £65,663 TST and IGRA in all: £93,211 IGRA and CXR in all: £63,074 | PSA showed 'No testing' to be the most likely cost effective approach up to a threshold of £30,000 per QALY gained | Testing for TB in an adult HIV population seems marginally cost effective, but IGRA testing of only patients from sub Saharan Africa is the most cost effective strategy | None reported |
| Capocci 2016a [25] | QALYs gained per 10,000 over no testing TST in BA: 2.09 TST in BA and MI: 2.43  IGRA in BA: 3.85 IGRA in BA and MI: 5.1  IGRa in all: 5.72 TST and IGRA in all: 5.88 | Costs per 10,000 over no testing TST in BA: £749,660 TST in BA and MI: £761,797  IGRA in BA: £812,048 IGRA in BA and MI: £865,959 IGRA in all: £1,056,702 TST and IGRA in all: £1,219,154 | ICER per QALY gained over no testing TST in BA: £23,429 TST in BA and MI: £25,218  IGRA in BA: £28,971 IGRA in BA and MI: £32,410 IGRa in all: £62,209 TST and IGRA in all: £88,139 | With PSA, no strategy was most likely cost-effective up to £30,000/QALY compared to no testing | Testing all HIV patients in the UK with high uptake ART is unlikely to be cost-effective regardless of testing strategy employed. Testing of Black Africans or migrants from middle incidence countries is, at best, marginally cost effective. | None reported |
| Capocci 2015 [23] | **2000-2005 (n=2209)** BHIVA vs no testing: 6.44 NICE vs BHIVA: 2.45 All vs NICE: 0.72  **2005-2010 (n=2902)**  BHIVA vs no testing: 6.36 NICE vs BHIVA: 1.80 All vs NICE: 0.66 | **Average cost per patient 2000-2005** No testing: €153 BHIVA: €171 NICE: €179 All:€190  **2005-2010**  No testing: €110 BHIVA: €131 NICE: €151 All: €168 | **2000-2005 (n=2209)** BHIVA vs no testing: €6270 NICE vs BHIVA: €6998 All vs NICE: €33,473  **2005-2010 (n=2902)**  BHIVA vs no testing: €9332 NICE vs BHIVA: €32,564 All vs NICE: €74,067 | Testing became more cost-effective as IGRA cost fell, uptake and effectiveness of LTBI increased and cost of active TB treatment rose. PSA showed that at a WTP of £20,000/QALY, the NICE testing strategy had the highest number of iterations where it was most cost-effective between 2000 and 2005 and the BHIVA strategy the most between 2005 and 2010 | The percentage of patients on ART increased between 2000 and 2010 and the percentage of new HIV patients from high incidence TB countries decreased. This impacted on the cost effectiveness of different strategies. All strategies were cost-effective compared to no testing up to 2005, but more targeted strategies found in the BHIVA guideline were most cost-effective after 2005. If the changes seen between 2000 and 2010 were to continue, increasingly targeted testing strategies would be required for testing to be cost-effective | The study was retrospective and the landscape seemed to be changing rapidly meaning modelling is required to look at how the landscape may change in the future Onward transmission was not included Costs of hepatoxicity from LTBI treatment not included in the base case and costs of IGRA are probably overestimated |
| Capocci 2014 [22] | NR | NR | ICER of £21,475 for BHIVA strategy over no testing.  BHIVA dominated the NICE strategy.  All-comers strategies (not described) were all above £20,000-£30,000 per QALY gained | NR | Only testing HIV patients with the highest risk of progression to active TB in a population with high ART use is cost-effective. The BHIVA strategy dominated the NICE strategy. | NR |
| Capocci 2012 [21] | **QALYs gained for 3306 people in study over no testing**  NICE: 25 BHIVA: 16 | **Costs gained for 3306 people in study** No testing: £367,200 NICE: £335,886 BHIVA: £345,848 | No ICERs as NICE strategy dominates all strategies and BHIVA dominates no testing | NR | Both the BHIVA and NICE strategies were less expensive and produced more QALYs than no testing, but the NICE strategy dominated the BHIVA strategy | NR |
| Kowada 2013 [27] | TST/QFT: 4.34437 TST/T-SPOT: 4.34419 QFT: 4.34376 T-SPOT: 4.34331 TST: 4.33961 No screening: 1.34383  CXR: 4.34950 | TST/QFT: 491.7 TST/T-SPOT: 499.0 QFT: 559.8 T-SPOT: 577.4 TST: 687.7 No screeni761.7ng:  CXR: 6801.0 | TST/QFT: - TST/T-SPOT: Dominated QFT: Dominated T-SPOT: Dominated TST: Dominated No screening: Dominated CXR: 1,231,827.4 | At a WTP threshold of $50,000 per QALY gained, QFT was more cost effective than TST/QFT when LTBI rate was <0.18 and when BCG vaccination rate was >0.95 | Effective LTBI screening using IGRA is recommended to prevent TB transmission not only in nursing homes but also in local communities in low-incidence countries | Sensitivities and specifities of TB screening kits (IGRA and TST) were obtained from meta-analyses of immunocompetent individuals, not older people with waning immunity There is little data on LTBI rates using IGRAs in nursing home residents Harm from radiation exposure by repeating CXR not considered Use of rifapentine plus isoniazid for 3 months (higher treatment completion rate) was not considered as a chemoprophylaxis regimen Further epidemiology of TB in elderly is needed to make a convincing case for TB policy change No method for diagnosing whether LTBI differentiates first infection with TB from reinfection Few epidemiological studies of TB outbreaks in nursing homes Different costs and medical systems between countries may impact on generalisability of findings |
| Kowada 2014 [18] | Non-BCG vaccinated cohort during pregnancy TST/QFT: 20. TST/T-SPOT: 20. TST: 20. QFT:20. T-SPOT: 20.  BCG vaccinated cohort during pregnancy TST/QFT: 20. TST/T-SPOT: 20. QFT: 20. T-SPOT: 20. TST: 20.  Non-BCG vaccinated cohort in postpartum period TST/QFT: 20. TST/T-SPOT: 20. TST: 20. QFT: 20. T-SPOT: 20.  BCG vaccinated cohort in postpartum period TST/QFT: 20. TST/TSPOT: 20. QFT: 20. T-SPOT: 20. TST: 20. | Non-BCG vaccinated cohort during pregnancy TST/QFT: 3760.54 TST/T-SPOT: 3769.78 TST: 3966.83 QFT:3971.73 T-SPOT: 4042.17  BCG vaccinated cohort during pregnancy TST/QFT: 3813.75 TST/T-SPOT: 3841.22 QFT: 3966.83 T-SPOT: 4042.17 TST: 6174.14  Non-BCG vaccinated cohort in postpartum period TST/QFT: 3760.54 TST/T-SPOT: 3769.78 TST: 3966.83 QFT: 3971.73 T-SPOT: 4042.17  BCG vaccinated cohort in postpartum period TST/QFT: 3813.75 TST/TSPOT: 3841.22 QFT: 3966.83 T-SPOT: 4042.17 TST: 6174.14 | Non-BCG vaccinated cohort during pregnancy TST/QFT: -  TST/T-SPOT: Dominated  TST: Dominated  QFT: Dominated  T-SPOT: Dominated   BCG vaccinated cohort during pregnancy TST/QFT: - TST/T-SPOT: Dominated  QFT: Dominated  T-SPOT: Dominated  TST: Dominated   Non-BCG vaccinated cohort in postpartum period TST/QFT: - TST/T-SPOT: Dominated  TST: Dominated  QFT: Dominated  T-SPOT: Dominated   BCG vaccinated cohort in postpartum period TST/QFT: - TST/TSPOT: Dominated  QFT: Dominated  T-SPOT: Dominated  TST: Dominated | One way SA results were sensitive to the sensitivity of T-SPOT and the sensitivity of QFT in non-BCG vaccinated occasional screening pregnant women | Using an IGRA for TB screening of high-risk HIV +ve pregnant women in low TB incidence countries is recommended on the basis of cost effectiveness | Estimates of each of the variables in the model were obtained from studies conducted in numerous countries The sensitivity and specificity estimates for IGRAs in HIV+ve pregnant women were obtained from meta-analyses of published literature and assumptions gut little is known on effect of pregnancy on sensitivity and specificity of IGRAs and TST Prevention of TB spread by pregnant women is a more urgent problem in developing countries than in developed countries Routine use of chest radiography in the absence of clear symptoms of active TB may be unwarranted and raise ethical considerations Women may be less likely than men to submit good-quality sputum The use of chemoprophylaxis for pregnant women is controversial There is little data of TB incidence and LTBI prevalence in HIV +ve pregnant women There is no data of TB risk in pregnancy trimester Different countries have different policies and resources for TB screening - generalisability of results unclear Costs of the side effects of MDR-TB therapy was not included in the model Use of chemoprophylaxis for pregnant women is controversial |

Key: 3HP - once-weekly isoniazid- rifapentine for 12 weeks; 4R – 4 months rafimpin; 6H - 6 months isoniazid monotherapy; BCG - Bacillus Calmette–Guérin; CXR – Chest X-ray; IGRA - Interferon Gamma Release Assay; INH – isoniazid; LTBI – Latent tuberculosis infection; MDR – multi drug resistant; NR – Not recorded; QFT – QuantiFERON; RIF – rafimpin; TSPOT - T-Spot TB test; TST – Tuberculin Skin Test; ICER – Incremental Cost-Effectiveness Ratios; PSA – Probabilistic Sensitivity Analysis; QALY – Quality Adjusted Life Year;

# APPENDIX D

# Included studies full extraction tables: Contacts

Table D.1 Contacts: Population information and tests considered

| Author/ Trial ID | Country/region | Description of population | Tests assessed | Prevalence rate of LTBI | BCG vaccination rate | TB activation rate | Secondary transmission rate |
| --- | --- | --- | --- | --- | --- | --- | --- |
| Abubakar 2018 [16] | UK | All over 16 years of age attending participating TB clinics or primary care centres for screening  Contacts of active TB  Individuals with a cumulative duration of exposure of >8 hours to the relevant index case in a confined space during the period of infectiousness (prior to initiation of treatment). Migrants New entrants arriving in the UK in the last 5 years from high-incidence countries  Born in high-incidence countries who entered the UK > 5 years ago, but who had spent >1 year (cumulative) in the past 5 years in a high-incidence country | TST, IGRA, T-SPOT.TB, QFT-GIT | Year 1: 0.00932 per person year Year 2: 0.00115 per person year Declines further over time with a Weibull (0.00374 mean with 2.5 hazard multiplier in year 1 and shape parameter of 6.0) | 79.8% of migrants had BCG vaccination. Assumed 94% uptake in those without LTBI who are offered BCG vaccination. BCG vaccination averts 49% of TB cases | NR | Secondary transmission not included in base case but scenario analysis explored contact tracing 4 cases for every case of active TB that developed |
| Hayama 2017 [29] | UK | A London TB clinic | TST alone, IGRA (QFT-GIT or T-SPOT) alone, TST +ve followed by IGRA, TST -ve followed by IGRA | NR | 75.3% were vaccinated | NR | NR |
| Kowada 2015 [30] | Japan | Recent arrivals | IGRA ((QFT-GIT or T-SPOT), TST | Base case (range for SA) 0.0237 (0.0150-0.0345) | NR | Proportion still infected post-LTBI treatment: 0.345 Average number of secondary cases from one index case: 0.2 (0.1-0.3) Average delay from infection to activation (secondary cases): 2.88 | NR |
| Kowada 2014 [18] | Japan | Pregnant HIV +ve women | TST QFT T-SPOT TST/QFT TST/T-SPOT CXR | Incidence of TB among 20 year old HIV+ve pregnant women in postpartum period: 0.0003 (0.00002-0.00065) TB risk during pregnancy: 1.29 (95% cI: 0.82-2.03) TB risk in postpartum period: 1.95 (1.24-3.07) | NR | Increased likelihood of progression from LTBI to active TB among HIV positive pregnant women: 4.5 (95%CI: 1.1-18.0) | NR |
| Linas 2016 [7, 8] | USA | High risk populations | TST, IGRA | Adult: 41.4% Children: 7.0% | NR | Adult: 0.079 per 100 person years.  Children: 0.079 per 100 person years Reduced by 10% per decade | 0.31 |

Key: 3HP - once-weekly isoniazid- rifapentine for 12 weeks; 4R – 4 months rafimpin; 6H - 6 months isoniazid monotherapy; BCG - Bacillus Calmette–Guérin; CXR – Chest X-ray; IGRA - Interferon Gamma Release Assay; INH – isoniazid; LTBI – Latent tuberculosis infection; MDR – multi drug resistant; NR – Not recorded; QFT – QuantiFERON; RIF – rafimpin; TSPOT - T-Spot TB test; TST – Tuberculin Skin Test

Table D.2 Contacts: Modelling methods

| Author/ Trial ID | Model type | Time horizon | Health states | Perspective | Discounting |
| --- | --- | --- | --- | --- | --- |
| Abubakar 2018 [16] | Decision tree and patient level discrete event simulation model | Lifetime | **Decision tree**  Determinate/indeterminate result (TST only) +ve/-ve tests in isolation or combination  +ve/-ve CXR Gastric lavage Active TB and treated/no active TB (LTBI) Treatment adherence/non-adherence Accept/refuse LTBI treatment LTBI treatment adherence/non-adherence  **DES model** Active TB on treatment Active TB not on treatment No TB and on CPX (chemoprophylaxis) No TB and non on CPX | NHS | 3.5% pa (costs and benefits) |
| Hayama 2017 [29] | Markov decision analytic model | Lifetime | NR In supplement but supplement not available | NHS perspective | Costs and QALYs discounted at 3.5% per year |
| Kowada 2015 [30] | Decision tree followed by a Markov model. | 50 years | Decision tree Test result  CXR if +ve  LTBI treatment adherence w/wout complication   Markov model Healthy (no TB and no LTBI) LTBI TB Dead | Public health | 3% for costs and benefits |
| Kowada 2014 [18] | Decision tree followed by a Markov model. | 30 years | Decision tree Test result  CXR if +ve  LTBI treatment adherence w/wout complication   Markov model Healthy (no TB and no LTBI) LTBI TB Dead | Public health payer | 3% for costs and benefits |
| Linas 2016 [7, 8] | Decision trees with Markov models | Lifetime | **In decision tree (nodes)** Test result (positive or negative) Adherence to therapy Complications from therapy  **In Markov mode**l INH therapy without toxicity Non-fatal INH hepatitis Active TB Post Active TB Death | Public health payer | 3% pa (costs and benefits) |

Key: 3HP - once-weekly isoniazid- rifapentine for 12 weeks; 4R – 4 months rafimpin; 6H - 6 months isoniazid monotherapy; BCG - Bacillus Calmette–Guérin; CXR – Chest X-ray; IGRA - Interferon Gamma Release Assay; INH – isoniazid; LTBI – Latent tuberculosis infection; MDR – multi drug resistant; NR – Not recorded; QFT – QuantiFERON; RIF – rafimpin; TSPOT - T-Spot TB test; TST – Tuberculin Skin Test

Table D.3 Contacts: Cost and utility parameter values

| Author/ Trial ID | Sources of utility values | Utility values used in model (mean (SD)) | Cost year | Currency | Source of test costs | Test costs in model | Sources of treatment costs | Treatment costs in model | Sources of other costs | Other costs in model |
| --- | --- | --- | --- | --- | --- | --- | --- | --- | --- | --- |
| Abubakar 2018 [16] | Kowada | **Decrements** Active TB (while on treatment): 0.15 Treatment for LTBI: 0.001 | 2012/13 | GB£ | Pooran et al and NHS Reference costs 2012/13 | TST: 17.48 QFT-GIT: 48.73 T-SPOT.TB: 59.57 CXR:35.00 | Bothamley et al NHS drug tariff Pareek et al | Adherence to active TB treatment: 5461.12 Non-adherence to active TB treatment: 910.19 Adherence to LTBI treatment: 677.07 Non-adherence to LTBI treatment: 112.85 Treatment of isoniazid-induced hepatitis: 389.51 | NHS Reference Costs 2012/13 | Gastric lavage procedure: 916.00 Sputum examination: 7.00 |
| Hayama 2017 [29] | Tsevat 1988 | Healthy: 1 LTBI: 1 LTBI with chemoprophylaxis (9 months): 0.996 Non-fatal active TB during and before treatment: 0.85 Dead: 0 | 2009 | US$ | Medical insurance re-imbursement Yoshiyama 2000 | QFT: 64.5 (32.3-129) TST: 17.2 (8.6-34.4) CXR: 40.5 (20.3-81) | Yoshiyama 2000 | Chemoprophylaxis by INH for 9 months: 864.3 (432.2-1,728.6) Treatment of INH-induced hepatitis by INH chemoprophylaxis: 13,298 (6,649-26,596) Treatment of TB for 6 months: 16,623 (8311-33,246) | Medical insurance re-imbursement | Smear and culture of sputum examination: 78.1 (39.1-156.2) |
| Kowada 2015 [30] | Guo 2009 | Well: 1.00 LTBI: 1.00 LTBI with chemoprophylaxis without complication: 0.98 LTBI with chemoprophylaxis with liver dysfunction: 0.85 Active TB during and before treatment: 0.80 Dead: 0.00 | 2012 | Japanese yen converted to US$ | Medical insurance reimbursement table in Japan Ministry of Health, Labour and Welfare (Japan) | 2012 US$ QFT: 60.6 (30.3-120.2) T-SPOT: 60.6 (30.3-0.2) TST: 15.4 (7.7-30.8) CXR: 36.3 (18.2-61.6) | National fee schedule and medical insurance reimbursement, Japan | 2012 US$ Chemoprophylaxis (INH), 9 months: 773.6 (386-1547.2) Treatment of INH-induced hepatitis: 11,903 (5951.5-23,809) Treatment of TB (6 months): 14,879 (7440-29,758) Physicians (hourly): 52.6 (26.3 (105.3) Radiology/laboratory technicians (hourly): 23.0 (11.5-46.0) | Japanese standard procedure document for non-smoking medical treatment | NRT: 411.5 (205.8-823) |
| Kowada 2014 [18] | Guo 2009 Resch 2006 | Non-LTBI and non-TB in HIV +ve pregnant state: 1 LTBI taking no chemoprophylaxis in HIV positive pregnant state: 1 LTBI taking chemoprophylaxis without complication in HIV positive pregnant state: 0.99 LTBI taking chemoprophylaxis with liver dysfunction in HIV positive pregnant state: 0.85 Active non MDR-TB during treatment and before in HIV +ve pregnant state: 0.80 Active MDR-TB during treatment and before in HIV positive pregnant state: 0.58 Dead: 0 | 2012 | US$ | Medical insurance reimbursement table | QFT: 60.6 (30.3-120.2) T-SPOT: 60.6 (30.3-120.2) TST: 15.4 (7.7-30.8) CXR: 36.3 (18.2-61.6) | Kowada 2008 Resch 2006 | INH chemoprophylaxis for 6 months: 515.7 (257.9-1,035.4) Treatment of INH-induced hepatitis by chemoprophylaxis: 11,903 (5951.5-23,806) Treatment of non MDR-TB for 6 months: 14,879 (7440-29578) Treatment of MDR-TB: 192,921 (96,461-385842) | Medical insurance reimbursement table | Smears and cultures of sputum examination: 69.9 (35.0-139.8) |
| Linas 2016 [7, 8] | Published sources (Dion 2002, Dion 2004, Marra 2008,) | INH therapy without toxicity: 1.0 Non-fatal INH hepatitis: 0.85 Active TB: 0.8 Month of TB or INH death: 0.3 Post TB: 1.0 | 2011 | US$ | Published sources (including government data and literature - unclear where test costs were actually derived) | IGRA: $51.77 TST: $22.09 (placing and reading) | Published sources (including government data and literature - unclear where costs were actually derived) | INH per month: $2.83 Physician visit per month on INH: $48.51 | Published sources (including government data and literature - unclear where costs were actually derived) | Cost of nonfatal INH hepatitis: £182.61 Cost of hospitalisation with nonfatal INH hepatitis: $7,906.66 First month active TB: $11,021.28 (of which $10,071.78 hospitalisation and drugs) Second month active TB: $1,116.21 Third month+ active TB: $309.92 |

Key: 3HP - once-weekly isoniazid- rifapentine for 12 weeks; 4R – 4 months rafimpin; 6H - 6 months isoniazid monotherapy; BCG - Bacillus Calmette–Guérin; CXR – Chest X-ray; IGRA - Interferon Gamma Release Assay; INH – isoniazid; LTBI – Latent tuberculosis infection; MDR – multi drug resistant; NR – Not recorded; QFT – QuantiFERON; RIF – rafimpin; TSPOT - T-Spot TB test; TST – Tuberculin Skin Test

Table D.4 Contacts: Sensitivity and specificity of LTBI tests

| Author/ Trial ID | Source of test accuracy | Sensitivity | | | | | Specificity | | | | |
| --- | --- | --- | --- | --- | --- | --- | --- | --- | --- | --- | --- |
|  |  | *IGRA* | *QFT* | *TSPOT* | *TST* | *CXR* | *IGRA* | *QFT* | *TSPOT* | *TST* | *CXR* |
| Abubakar 2018 [16] | Drawn directly from PREDICT trial. HRs for combinations of tests also provided. Highest HR (and so highest identification of cases) was 0.02003 for TST+(≥6mm or ≥15mm with BCG) in combination with a positive T-SPOT test | NR | **Hazard of developing TB** **per person year** Positive: 0.01039  Negative: 0.00187 | **Hazard of developing TB** **per person year** Positive: 0.01360  Negative: 0.00149 | **Hazard of developing TB** **per person year** TST+(>=5mm): 0.00682  TST-(<5mm):0.00117 TST+(>=6mm or >=15mm with BCG): 0.01121 TST-(<6mm or <15mm with BCG): 0.00157 | NR | NR | NR | NR | NR | NR |
| Hayama 2017 [29] | NR | NR | NR | NR | NR | NR | NR | NR | NR | NR | NR |
| Kowada 2015 [30] | NR | NR | 0.84 (0.81-0.87) | 0.89 (0.86-0.91) | 0.77 (0.71-0.82) | 0.7 (0.59-0.82) |  | 0.99 (0.98-1.00) | 0.98 (0.94-0.99) | Among BCG-vaccinated contacts: 0.59 (0.46-0.73) Among non-BCG vaccinated contacts: 0.97 (0.95-0.99) | 0.6 (0.52-0.63) |
| Kowada 2014 [18] | Literature | NR | 0.61 (95%CI:0.54-0.67) | 0.65 (95%CI:0.56-0.74) | 0.43 (0.37-0.5) | For active TB 0.7 (0.59-0.82) |  | 0.99 (95%CI:0.98-1.0) | 0.98 (95%CI:0.94-0.99) | For LTBI BCG vaccinated: 0.59 (95%CI: 0.46-0.73) Non-BCG vaccinated: 0.97 (95%CI:0.95-0.99) | For active TB 0.6 (0.52-0.63) |
| Linas 2016 [7, 8] | Mazurek 2010, Oxlade 2007, Harada 2008, Detjen 2007, Lee 2006, Palazzo 2008, Ruhwald 2008, Aichelburg 2009, Bartu 2008, Chee 2008, Tsiouris 2006 | 0.83 | NR | NR | 0.89 | NR | 0.99 | NR | NR | Foreign born: 0.92 US born: 0.98 | NR |

Key: 3HP - once-weekly isoniazid- rifapentine for 12 weeks; 4R – 4 months rafimpin; 6H - 6 months isoniazid monotherapy; BCG - Bacillus Calmette–Guérin; CXR – Chest X-ray; IGRA - Interferon Gamma Release Assay; INH – isoniazid; LTBI – Latent tuberculosis infection; MDR – multi drug resistant; NR – Not recorded; QFT – QuantiFERON; RIF – rafimpin; TSPOT - T-Spot TB test; TST – Tuberculin Skin Test

Table D.5 Contacts: Treatments for LTBI

| Author/ Trial ID | LTBI treatments considered | Proportion of patients starting treatment | Efficacy of treatment | Percentage maintaining treatment | INH related hepatitis | INH related deaths | Percentage with drug resistant TB | Percentage with mulit-drug resistant TB | TB death rates |
| --- | --- | --- | --- | --- | --- | --- | --- | --- | --- |
| Abubakar 2018 [16] | 3HP | 94% (but only offered to under 35s reflecting UK practice) | TB cases with complete CPX: 0.33 TB cases with incomplete CPX: 0.78 | CPX completion rate: 0.85 | 0.0040 (CPX AE) | 0.00002 (death from CPX AE) | NR | NR | 15-44: 0.012 45-64: 0.048 ≥65: 0.176 |
| Hayama 2017 [29] | NR | NR | NR | NR | NR | NR | NR | NR | NR |
| Kowada 2015 [30] | INH | NR | Efficacy: 0.8 | Rate of adherence to standard 9-month INH chemoprophylaxis protocol for IGRAs: 0.8 (0-1) Rate of adherence to standard 9-month INH chemoprophylaxis protocol for TST: 0.5 (0-1) [assumption] | Probability of INH-induced hepatitis by chemoprophylaxis: 0.003 (0.001-0.04) | NR | NR | NR | Increased rate of TB mortality among contacts who smoke cf general population: 8.56 (2.5-12.0) |
| Kowada 2014 [18] | INH | NR | Efficacy of LTBI treatment: 0.68 | Adherence rate of standard 6-month INH chemoprophylaxis protocol for IGRAs: 0.8 (0-1) Adherence rate of standard 6-month INH chemoprophylaxis protocol for TST: 0.5 (0-1) | Probability of INH induced hepatitis by INH prophylaxis: 0.011 (0.003-0.061) | NR | NR | NR | Increased likelihood of mortality by active TB among HIV+ve pregnant women: 5.2 (95% CI:1.7-15.6) |
| Linas 2016 [7, 8] | INH | 0.9 | Risk reduction with INH: 3-5mth completed: 30% 6-8mth completed: 60% 9mth completed: 90% | 48% | <35 years: 0.001 >=35 years: 0.01 | 0.01 per case of hepatitis | NR | NR | No medical comorbidities: 0.05 Chronic conditions: 0.06 |

Key: 3HP - once-weekly isoniazid- rifapentine for 12 weeks; 4R – 4 months rafimpin; 6H - 6 months isoniazid monotherapy; BCG - Bacillus Calmette–Guérin; CXR – Chest X-ray; IGRA - Interferon Gamma Release Assay; INH – isoniazid; LTBI – Latent tuberculosis infection; MDR – multi drug resistant; NR – Not recorded; QFT – QuantiFERON; RIF – rafimpin; TSPOT - T-Spot TB test; TST – Tuberculin Skin Test

Table D.6 Contacts: Results

| Author/ Trial ID | Total QALYs by strategy | Total costs by strategy | Incremental analyses, e.g. ICER (per QALY gained) | Sensitivity analyses outcomes | Author conclusions | Limitations identified by author |
| --- | --- | --- | --- | --- | --- | --- |
| Abubakar 2018 [16] | TST+(>=5mm): 22.131076 TST+(>=5mm)+IGRA: 22.131048 TST+(>=6mm or >=15mm with BCG): 22.131074 TST+(>=5mm)+T-SPOT: 22.131021 TST+(>=6mm or >=15mm with BCG)+IGRA: 22.131047 TST+(>=5mm)+QFT: 22.131019 T-SPOT: 22.131031 TST+(>=6mm or >=15mm with BCG)+T-SPOT: 22.131021 QFT: 22.131027 TST+(>=6mm or >=15mm with BCG)+QFT: 22.131019 No test: 22.13084 | TST+(>=5mm): £301.53 TST+(>=5mm)+IGRA: £241.73 TST+(>=6mm or >=15mm with BCG): £224.24 TST+(>=5mm)+T-SPOT: £212.93 TST+(>=6mm or >=15mm with BCG)+IGRA: £205.05 TST+(>=5mm)+QFT: £204.41 T-SPOT: £204.07 TST+(>=6mm or >=15mm with BCG)+T-SPOT: £191.09 QFT: £187.25 TST+(>=6mm or >=15mm with BCG)+QFT: £184.73 No test: £28.57 | **INB (vs no test)**  TST+(>=5mm): -£268.22 TST+(>=5mm)+IGRA: -£208.99 TST+(>=6mm or >=15mm with BCG): -£190.97 TST+(>=5mm)+T-SPOT: -£180.74 TST+(>=6mm or >=15mm with BCG)+IGRA: -£172.33 TST+(>=5mm)+QFT: -£172.26 T-SPOT: -£171.66 TST+(>=6mm or >=15mm with BCG)+T-SPOT: -£158.90 QFT: -£154.57 TST+(>=6mm or >=15mm with BCG)+QFT: -£152.57 | Testing becomes more cost-effective as baseline incidence of TB increases. If T-SPOT costs were equal to QFT then T-SPOT becomes the optimal testing strategy. If CPX uptake was lower (59.9%) and completion was lower (57%) then QFT becomes the most cost-effective strategy. In a scenario with secondary contacts no testing remained the dominant strategy. | There were modest differences between tests or combinations of tests, in identifying individuals who would go on to develop active TB. However, a two-step approach that combined TST based upon prior BCG vaccination with an IGRA test was most cost-effective although no strategy had a positive NMB at £20,000 QALY compared to no test | Participants who have left the UK would not have been included in the progression data resulting in an underestimate of the overall progression rate.  Comorbidities were self-reported.  Unable to model the full population effect of better LTBI testing and treatment on the transmission of M. tuberculosis and so results likely underestimate the health benefits and cost savings associated with preventing progression through LTBI testing. The model included a number of simplifying assumptions and some of the sources for input parameter estimates were weak |
| Hayama 2017 [29] | QFT-Git alone averted 1.6 TB cases per 1000 contacts compared to TST +ve followed by QFT-GIT | NR | QFT-GIT was the most cost-effective strategy ( £6876 per QALY gained) compared to TST +ve followed by QFT-GIT | NR | Of the considered strategies, QFT-GIT is preferable for LTBI screening in adult close contacts of TB cases in London | NR |
| Kowada 2015 [30] | QFT: 25.40801 T-SPOT: 25.44099 TST: 24.30704 TST+NRT: 24.38557 QFT+NRT: 25.4616 T-SPOT+NRT: 25.52039 | QFT: 11,061.87 T-SPOT: 11,197.93 TST: 11,322.96 TST+NRT: 11,517.99 QFT+NRT: 11,697.02 T-SPOT+NRT: 11,773.47 | QFT: - T-SPOT: 4,125.05 TST: -237.15 TST+NRT: -446.11 QFT+NRT: 11,850.45 T-SPOT+NRT: 6,332.23 | Results were sensitive to the effectiveness of NRT and the rate of mortality reduction by NRT (WTP US$50,000 per QALY gained) T-SPOT was more CE than TST+NRT when NRT effectiveness was <0.016 and when the mortality reduction rate due to NRT was <0.035 | Screening using IGRA, rather than TST, combined with NRT is more cost-effective among contacts who smoke | Assumption that reductions in TB mortality and age-specific all-cause mortality occur during the first year of NRT (data on the reduction of mortality using NRT are limited and further studies on the reduction of TB risk among smokers with tobacco cessation are limited) Uncertainty around the RR of TB due to tobacco smoking value used in the model Sources of values for model parameters were obtained from studies carried out in multiple countries Passive smoking was not taken into account (this has been shown to increase risk of TB) Generalisability of findings unclear (costs and policies vary by country) Different smoking cessation strategies not considered Benefits (other than TB risks and mortality) of not smoking not included in the model |
| Kowada 2014 [18] | Non-BCG vaccinated cohort during pregnancy T-SPOT: 20.82595 QFT: 20.82516 TST: 20.81890 TST/T-SPOT:20.81856 TST/QFT: 20.81812  BCG vaccinated cohort during pregnancy T-SPOT: 20.82595 QFT: 20.82516 TST/T-SPOT: 20.81745 TST/QFT: 20.81748 TST: 20.75916  Non-BCG vaccinated cohort in postpartum period T-SPOT: 20.62162 QFT: 20.60887 TST: 20.57214 TST/T-SPOT: 20.54227 TST/QFT: 20.53851  BCG vaccinated cohort in postpartum period T-SPOT: 20.62162 QFT: 20.60887 TST/T-SPOT: 20.54135 TST/QFT: 20.53796 TST: 20.52988 | Non-BCG vaccinated cohort during pregnancy T-SPOT: 8917.53 QFT: 8983.62 TST: 9513.82 TST/T-SPOT:9837.47 TST/QFT: 9882.02  BCG vaccinated cohort during pregnancy T-SPOT: 8917.53 QFT: 8983.62 TST/T-SPOT:9884.47 TST/QFT9917.62 TST: 11159.91  Non-BCG vaccinated cohort in postpartum period T-SPOT: 7552.91 QFT: 7567.28 TST: 7810.27 TST/T-SPOT: 7911.25 TST/QFT: 7928.35  BCG vaccinated cohort in postpartum period T-SPOT: 7552.91 QFT: 7567.28 TST/T-SPOT:7947.93 TST/QFT7954.60 TST: 8942.45 | Non-BCG vaccinated cohort during pregnancy T-SPOT: - QFT: Dominated TST: Dominated TST/T-SPOT: Dominated TST/QFT: Dominated  BCG vaccinated cohort during pregnancy T-SPOT: - QFT: Dominated TST/T-SPOT: Dominated TST/QFT: Dominated TST: Dominated  Non-BCG vaccinated cohort in postpartum period T-SPOT: - QFT: Dominated TST: Dominated TST/T-SPOT: Dominated TST/QFT: Dominated  BCG vaccinated cohort in postpartum period T-SPOT: - QFT:  TST/T-SPOT: TST/QFT: TST: | One way SA results were sensitive to the - sensitivity and specificity of T-SPOT and QFT during pregnancy - sensitivity of T-SPOT and QFT in postpartum | Using an IGRA for TB screening of high-risk HIV +ve pregnant women in low TB incidence countries is recommended on the basis of cost effectiveness | Estimates of each of the variables in the model were obtained from studies conducted in numerous countries The sensitivity and specificity estimates for IGRAs in HIV+ve pregnant women were obtained from meta-analyses of published literature and assumptions gut little is known on effect of pregnancy on sensitivity and specificity of IGRAs and TST Prevention of TB spread by pregnant women is a more urgent problem in developing countries than in developed countries Routine use of chest radiography in the absence of clear symptoms of active TB may be unwarranted and raise ethical considerations Women may be less likely than men to submit good-quality sputum The use of chemoprophylaxis for pregnant women is controversial There is little data of TB incidence and LTBI prevalence in HIV +ve pregnant women There is no data of TB risk in pregnancy trimester Different countries have different policies and resources for TB screening - generalisability of results unclear Costs of the side effects of MDR-TB therapy was not included in the model Use of chemoprophylaxis for pregnant women is controversial |
| Linas 2016 [7, 8] | Discounted quality adjusted life months presented in table **Adults** No screening: 281.14 TST: 281.27 IGRA: 281.28 **Children** No screening: 350.29 TST: 350.39 IGRA: 350.40 | **Adults** No screening: $125,510 TST: $125,610 IGRA: $125,620 **Children** No screening: $129,920 TST: $129,970 IGRA: $129,980 | **Adults** IGRA has an ICER per QALY gained of $21,500 compared to TST **Children** IGRA has an ICER per QALY gained of $21,100 per QALY gained compared to TST. | ICERs were sensitive to rates of reactivation (lower rates resulted in higher ICERs) and higher costs of IGRA testing (above $64). Other parameters were not found to significantly alter ICERs | LBTI screening would be cost effective in high risk groups in the USA including contacts. IGRA screening is more cost effective than TST screening | True activation rate of TB unknown, no gold standard test for TB, only direct medical costs included |

Key: 3HP - once-weekly isoniazid- rifapentine for 12 weeks; 4R – 4 months rafimpin; 6H - 6 months isoniazid monotherapy; BCG - Bacillus Calmette–Guérin; CXR – Chest X-ray; IGRA - Interferon Gamma Release Assay; INH – isoniazid; LTBI – Latent tuberculosis infection; MDR – multi drug resistant; NR – Not recorded; QFT – QuantiFERON; RIF – rafimpin; TSPOT - T-Spot TB test; TST – Tuberculin Skin Test; ICER – Incremental Cost-Effectiveness Ratios; PSA – Probabilistic Sensitivity Analysis; QALY – Quality Adjusted Life Year;

# APPENDIX E

# Included studies full extraction tables: Immunocompromised

Table E.1 Immunocompromised: Population information and tests considered

| Author/ Trial ID | Country/region | Description of population | Tests assessed | Prevalence rate of LTBI | BCG vaccination rate | TB activation rate | Secondary transmission rate |
| --- | --- | --- | --- | --- | --- | --- | --- |
| Auguste 2016 [15] | UK | Children | TST QFT-GIT T-SPOT.TB CXR | Base case (range for SA) 0.0288 (0.0206-0.0384) | Model not stratified by BCG status | Annualised reactivate rate: 0.013 (0.004-0.025) | Proportion still infected post-LTBI treatment: 0.345 Average number of secondary cases from one index case: 0.2 (0.1-0.3) Average delay from infection to activation (secondary cases): 2.88 |
| Kowada 2019 [31] | Japan | Solid organ (kidney, liver and lung) transplant recipients | QFR TSPOT TST CXR | NR | NR | NR | NR |
| Laskin 2013 [32] | USA | Recent arrivals | TST IGRA Questionnaire | Base case (range for SA) 0.0237 (0.0150-0.0345) | NR | Proportion still infected post-LTBI treatment: 0.345 Average number of secondary cases from one index case: 0.2 (0.1-0.3) Average delay from infection to activation (secondary cases): 2.88 | NR |
| Linas 2016 [7,8] | USA | High risk populations | TST, IGRA | Adult: 41.4% Children: 7.0% | NR | Adult: 0.079 per 100 person years.  Children: 0.079 per 100 person years Reduced by 10% per decade | 0.31 |
| Van der Have 2014 [33] | Europe | 30-60 year old patients with luminal CD about to commence TNF-alpha therapy | TST with CXR TST with CXR followed by QFT | LTBI: 9% | NR | 0.4% every three months on anti-TNF therapy | NR |

Key: 3HP - once-weekly isoniazid- rifapentine for 12 weeks; 4R – 4 months rafimpin; 6H - 6 months isoniazid monotherapy; BCG - Bacillus Calmette–Guérin; CXR – Chest X-ray; IGRA - Interferon Gamma Release Assay; INH – isoniazid; LTBI – Latent tuberculosis infection; MDR – multi drug resistant; NR – Not recorded; QFT – QuantiFERON; RIF – rafimpin; TSPOT - T-Spot TB test; TST – Tuberculin Skin Test

Table E.2 Immunocompromised: Modelling methods

| Author/ Trial ID | Model type | Time horizon | Health states | Perspective | Discounting |
| --- | --- | --- | --- | --- | --- |
| Auguste 2016 [19] | Decision tree followed by DES | 100 years | **Decision tree**  Determinate/indeterminate result (TST only) +ve/-ve tests in isolation or combination  +ve/-ve CXR Gastric lavage Active TB and treated/no active TB (LTBI) Treatment adherence/non-adherence Accept/refuse LTBI treatment LTBI treatment adherence/non-adherence  **Dynamic transmission model** No LTBI/TB LTBI Active TB Resolved TB Secondary infections Death all causes Death TB | NHS and Personal Social Services | 3.5% costs and outcomes |
| Kowada 2019 [37] | Decision tree followed by a Markov model.  Decision tree followed by a Markov model.  Decision tree followed by a Markov model. | Lifetime (length not reported) | Decision tree Test result  CXR if +ve  LTBI treatment adherence w/wout complication   Markov model Healthy (no TB and no LTBI) LTBI TB  Dead | Societal | 3% for costs and benefits |
| Laskin 2013 [38] | Decision tree followed by a Markov model. | Lifetime (length not reported) | Decision tree  T- Test result  CXR if +ve  LTBI treatment adherence w/wout complication  Markov model Well LTBI TB Dead | Societal | 3% for costs and benefits |
| Linas 2016 [11, 12] | Decision tree followed by a Markov model. | Lifetime | Decision tree  T- Test result  CXR if +ve  LTBI treatment adherence w/wout complication  Markov model Well LTBI TB Dead | Societal | 3% per year |
| Van der Have 2014 [39] | Decision trees with Markov models | Lifetime | **In decision tree (nodes)** Test result (positive or negative) Adherence to therapy Complications from therapy  **In Markov mode**l INH therapy without toxicity Non-fatal INH hepatitis Active TB Post Active TB Death | Health care costs | 3% pa (costs and benefits) |

Key: 3HP - once-weekly isoniazid- rifapentine for 12 weeks; 4R – 4 months rafimpin; 6H - 6 months isoniazid monotherapy; BCG - Bacillus Calmette–Guérin; CXR – Chest X-ray; DES – Discrete event simulation; IGRA - Interferon Gamma Release Assay; INH – isoniazid; LTBI – Latent tuberculosis infection; MDR – multi drug resistant; NR – Not recorded; QFT – QuantiFERON; RIF – rafimpin; TSPOT - T-Spot TB test; TST – Tuberculin Skin Test

Table E.3 Immunocompromised: Cost and utility parameter values

| Author/ Trial ID | Sources of utility values | Utility values used in model (mean (SD)) | Cost year | Currency | Source of test costs | Test costs in model | Sources of treatment costs | Treatment costs in model | Sources of other costs | Other costs in model |
| --- | --- | --- | --- | --- | --- | --- | --- | --- | --- | --- |
| Auguste 2016 [15] | Kowada | **Decrements** Active TB (while on treatment): 0.15 Treatment for LTBI: 0.001 | 2012/13 | GB£ | Pooran et al and NHS Reference costs 2012/13 | TST: 17.48 QFT-GIT: 48.73 T-SPOT.TB: 59.57 CXR:35.00 | Bothamley et al NHS drug tariff Pareek et al | Adherence to active TB treatment: 5461.12 Non-adherence to active TB treatment: 910.19 Adherence to LTBI treatment: 677.07 Non-adherence to LTBI treatment: 112.85 Treatment of isoniazid-induced hepatitis: 389.51 | NHS Reference Costs 2012/13 | Sputum examination: 7.00 |
| Kowada 2019 [31] (Kidney) | Marra 2008 | Healthy: 1.0 LTBI: 1.0 Non-MDR-TB taking chemoprophylaxis (9 months) with no complication: 1.0 Non-MDR-TB taking chemoprophylaxis (9 months) with complication (liver dysfunction): 0.85 Active non-MDR-TB during treatment and before: 0.8 Dead: 0.0 | 2012 | US$ | Medical insurance reimbursement table | QFT: 75.9 (38.0-151.8) TST: 19.3 (9.6-38.6) CXR: 45.4 (22.7-90.8) | Yoshiyama 2000 | Chemoprophylaxis by INH for 9 months: 968.4 (484.2-1,936.9) Treatment of INH-induced hepatitis by INH chemoprophylaxis: 14,900.2 (7,450.1-29,800.3) Treatment of TB for 6 months: 18,625.8 (9312.9-37,251.5) | Medical insurance reimbursement table | Smear and culture of sputum examination: 87.5 (43.8-175.0) |
| Kowada 2019 [31] (Liver) |  |  | US$ | 2016 | National fee schedule and medical reimbursement table in Japan Ministry of Health, Labor and Welfare (wage survey) | QFT: 62.0 (31.0-124.0) TSPOT: 62.0 (31.0-124.0) TST: 18.3: 9.1-36.6) CXR: 37.1 (18.6-74.2) | National fee schedule and medical reimbursement table in Japan Ministry of Health, Labor and Welfare (wage survey) | LTBI treatment with 9H: 1219.3 (609.7-2438.6) Treatment of drug induced hepatitis by LTBI treatment: 21,350 (10674-42700) Treatment of TB for 6 months: 33,573 (16,787-67,146) | National fee schedule and medical reimbursement table in Japan Ministry of Health, Labor and Welfare (wage survey) | Smear and culture of sputum examination: 163.3 (81.7-326.6-156.2) |
| Kowada 2019 [31] (Lung) | Guo 2009 Dion 2004 Vitale 2015 Villa 2012 | Well: 0.81 (fixed) LTBI: 0.81 (fixed) LTBI taking treatment without complication: 0.78 (fixed) LTBI taking treatment with liver dysfunction: 0.69 (fixed) TB during and before treatment: 0.65 Death: 0 | US$ | 2016 | National fee schedule and medical reimbursement table in Japan Ministry of Health, Labor and Welfare (wage survey) | QFT: 62.0 (31.0-124.0) TSPOT: 62.0 (31.0-124.0) TST: 18.3: 9.1-36.6) CXR: 37.1 (18.6-74.2) | National fee schedule and medical reimbursement table in Japan Ministry of Health, Labor and Welfare (wage survey) | LTBI treatment with 9H: 1219.3 (609.7-2438.6) Treatment of drug induced hepatitis by LTBI treatment: 21,350 (10674-42700) Treatment of TB for 6 months: 33,573 (16,787-67,146) | National fee schedule and medical reimbursement table in Japan Ministry of Health, Labor and Welfare (wage survey) | Smear and culture of sputum examination: 163.3 (81.7-326.6-156.2) |
| Laskin 2013 [32] | Guo 2009 Dion 2004 Vitale 2015 | Well: 0.71 (fixed) LTBI: 0.71 (fixed) LTBI taking treatment without complication: 0.69 (fixed) LTBI taking treatment with liver dysfunction: 0.60 (fixed) TB during and before treatment: 0.57 Death: 0 | US$ | 2016 | National fee schedule and medical reimbursement table in Japan Ministry of Health, Labor and Welfare (wage survey) | QFT: 62.0 (31.0-124.0) TSPOT: 62.0 (31.0-124.0) TST: 18.3: 9.1-36.6) CXR: 37.1 (18.6-74.2) | National fee schedule and medical reimbursement table in Japan Ministry of Health, Labor and Welfare (wage survey) | LTBI treatment with 9H: 1219.3 (609.7-2438.6) Treatment of drug induced hepatitis by LTBI treatment: 21,350 (10674-42700) Treatment of TB for 6 months: 33,573 (16,787-67,146) | National fee schedule and medical reimbursement table in Japan Ministry of Health, Labor and Welfare (wage survey) | Smear and culture of sputum examination: 163.3 (81.7-326.6-156.2) |
| Linas 2016 [7, 8] | Guo 2009 Dion 2004 Vitale 2015 Anyanwu 2001 | Well: 0.82 (fixed) LTBI: 0.82 (fixed) LTBI taking treatment without complication: 0.80 (fixed) LTBI taking treatment with liver dysfunction: 0.70(fixed) TB during and before treatment: 0.66 Death: 0 | US$ | 2016 | National fee schedule and medical reimbursement table in Japan Ministry of Health, Labor and Welfare (wage survey) | QFT: 62.0 (31.0-124.0) TSPOT: 62.0 (31.0-124.0) TST: 18.3: 9.1-36.6) CXR: 37.1 (18.6-74.2) | National fee schedule and medical reimbursement table in Japan Ministry of Health, Labor and Welfare (wage survey) | LTBI treatment with 9H: 1219.3 (609.7-2438.6) Treatment of drug induced hepatitis by LTBI treatment: 21,350 (10674-42700) Treatment of TB for 6 months: 33,573 (16,787-67,146) | National fee schedule and medical reimbursement table in Japan Ministry of Health, Labor and Welfare (wage survey) | Smear and culture of sputum examination: 163.3 (81.7-326.6-156.2) |
| Van der Have 2014 [33] | de Perio 2009 Eckman 2009 Gage 1996 Guo 2008 Tsevat 1988 Piccoli 1994 | Well: 1 Latent TB: 0.997 Active TB: 0.68 (0.65-0.72) NS: 0.90 INH hepatitis x 1 month: 0.85 Death: 0 | 2010 | US$ | Flaherman 2007 Centers for Medicare and Medicaid Services | TST:39.64 Questionnaire1.62 IGRA:84.56 | Anon, Paediatrics 2004 Centers for Medicare and Medicaid Services Statistics on Hospital Stays (Red Book)  de Perio 2009 | LTBI: 772.29 Active TB: 60,000.00 | de Perio 2009  Centers for Medicare and Medicaid Services Finnell 2009 Colquitt 2007 | Death (TB, hepatic failure or NS): 100,000 NS onset: 420.48 NS relapse: 140.16 |

Key: 3HP - once-weekly isoniazid- rifapentine for 12 weeks; 4R – 4 months rafimpin; 6H - 6 months isoniazid monotherapy; BCG - Bacillus Calmette–Guérin; CXR – Chest X-ray; IGRA - Interferon Gamma Release Assay; INH – isoniazid; LTBI – Latent tuberculosis infection; MDR – multi drug resistant; NR – Not recorded; QFT – QuantiFERON; RIF – rafimpin; TSPOT - T-Spot TB test; TST – Tuberculin Skin Test

Table E.4 Immunocompromised: Sensitivity and specificity of LTBI tests

| Author/ Trial ID | Source of test accuracy | Sensitivity | | | | | Specificity | | | | |
| --- | --- | --- | --- | --- | --- | --- | --- | --- | --- | --- | --- |
|  |  | *IGRA* | *QFT* | *TSPOT* | *TST* | *CXR* | *IGRA* | *QFT* | *TSPOT* | *TST* | *CXR* |
| Auguste 2016 [15] | NR | NR | 0.5548 (0.2473-0.8373)  Determinate QFT-GIT: 0.97 | 0.6665 (0.3517-0.9144)  Determinate T-SPOT.TB: 0.97 | ≥5mm: 0.3242 (0.1119-0.5848) ≥10mm: 0.1682 (0.0252-0.3899)  Conditional on -ve QFT-GIT (LTBI arm): 0.2775 (0.0121-0.7989) Conditional on +ve QFT-GIT (LTBI arm):0.4206 (0.0023-0.3891)  TST read: 0.94 (0.6-1.0) | NR | NR | 0.8227 (0.8052-0.8396) | 0.6846 (0.6346-0.7331) | <5mm: 0.7422 (0.7288-0.7557) <10mm: 0.8397 (0.7899-0.8831)  Conditional on -ve QFT-GIT (no LTBI arm): 0.44655 (0.3909-0.4993) Conditional on +ve QFT-GIT (no LTBI arm): 0.8058 (0.00006-0.8058) | NR |
| Kowada 2019 [31] | Literature | NR | For LTBI 0.70 (0.63-0.78) | NR | For LTBI 0.77 (0.71-0.82) | NR | NR | For LTBI 0.99 (0.98-1.0) | NR | For LTBI BCG vaccinated: 0.59 (0.46-0.73) Non-BCG vaccinated: 0.97 (0.95-0.99) | NR |
| Laskin 2013 [32] | QFT sensitivity: Diel 2010 QFT specificity: Diel 2011  TSPOT sensitivity: Diel 2010 TSPOT specificity: Diel 2011  TST sensitivity: Pai 2008 TST specificity: Pai 2008  CXR sensitivity and specificity: Tattevin 1999, Cohen 1996 | NR | 0.53 (95% CI:0.46-0.59) | 0.50 (95% CI: 0.42-0.59) | 0.31 (95% CI: 0.26-0.36) | NR | NR | 0.69 (95% CI: 0.65-0.72) | 0.67 (95% CI: 0.61-0.73) | Non-vaccinated BCG recipients: 0.63 (95%CI:0.6-0.65) Vaccinated BCG recipients: 0.38 (0.33-0.42) | NR |
| Linas 2016 [7, 8] | NR | 0.9 (0.77-0.99)  IGRA indeterminate: 0.03 (0.02-0.05) | NR | NR | 0.90 (0.88-1.00) | Questionnaire 0.46 | 0.97 (0.90-1.0) | NR | NR | 0.68 (0.52-0.84) | Questionnaire 0.94 |
| Van der Have 2014 [33] | Mazurek 2010, Oxlade 2007, Harada 2008, Detjen 2007, Lee 2006, Palazzo 2008, Ruhwald 2008, Aichelburg 2009, Bartu 2008, Chee 2008, Tsiouris 2006 | 0.83 | NR | NR | 0.89 | NR | 0.99 | NR | NR | Foreign born: 0.92 US born: 0.98 | NR |

Key: 3HP - once-weekly isoniazid- rifapentine for 12 weeks; 4R – 4 months rafimpin; 6H - 6 months isoniazid monotherapy; BCG - Bacillus Calmette–Guérin; CXR – Chest X-ray; IGRA - Interferon Gamma Release Assay; INH – isoniazid; LTBI – Latent tuberculosis infection; MDR – multi drug resistant; NR – Not recorded; QFT – QuantiFERON; RIF – rafimpin; TSPOT - T-Spot TB test; TST – Tuberculin Skin Test

Table E.5 Immunocompromised: Treatments for LTBI

| Author/ Trial ID | LTBI treatments considered | Proportion of patients starting treatment | Efficacy of treatment | Percentage maintaining treatment | INH related hepatitis | INH related deaths | Percentage with drug resistant TB | Percentage with mulit-drug resistant TB | TB death rates |
| --- | --- | --- | --- | --- | --- | --- | --- | --- | --- |
| Auguste 2016 [15] | INH | Accepting LTBI treatment: 0.9400 (0.50-1.0) | Proportion still infected at end of treatment: 0.345 | TB treatment adherence: 1.0 (not varied) | 0.0040 (0.001-0.010) | 0.00002 (0.00001-0.0001) | NR | NR | NR |
| Kowada 2019 [31] (Kidney) | 9H | NR | Efficacy: 0.8 | Adherence rate of 9H: 0.47 | Complication rate: 0.05 | NR | NR | NR | Mortality due to TB: 0.18 (0.1-0.5) |
| Kowada 2019 [31] (Liver) | 9H | NR | Efficacy: 0.8 | Adherence rate of 9H: 0.65 | Complication rate: 0.05 | NR | NR | NR | Mortality due to TB: 0.18 (0.1-0.5) |
| Kowada 2019 [31] (Lung) | 9H | NR | Efficacy: 0.8 | Adherence rate of 9H: 0.69 | Complication rate: 0.05 | NR | NR | NR | Mortality due to TB: 0.18 (0.1-0.5) |
| Laskin 2013 [32] | INH | NR | Efficacy of LTBI treatment: 0.75 | Assumed 100% | 0.004 | 0.00002 | NR | NR | Probability of death from TB on steroids: 0.01 (no range) |
| Linas 2016 [7, 8] | INH | 0.9 | Risk reduction with INH: 3-5mth completed: 30% 6-8mth completed: 60% 9mth completed: 90% | 50% | <35 years: 0.001 >=35 years: 0.01 | 0.01 per case of hepatitis | NR | NR | No medical comorbidities: 0.05 Chronic conditions: 0.06 |
| Van der Have 2014 [33] | INH | Assumed 100% | Activation rate falls from 0.40% to 0.16% per three months with INH | NR | NR | NR | NR | NR | 6% for active TB |

Key: 3HP - once-weekly isoniazid- rifapentine for 12 weeks; 4R – 4 months rafimpin; 6H - 6 months isoniazid monotherapy; BCG - Bacillus Calmette–Guérin; CXR – Chest X-ray; IGRA - Interferon Gamma Release Assay; INH – isoniazid; LTBI – Latent tuberculosis infection; MDR – multi drug resistant; NR – Not recorded; QFT – QuantiFERON; RIF – rafimpin; TSPOT - T-Spot TB test; TST – Tuberculin Skin Test

Table E.6 Immunocompromised: Results

| Author/ Trial ID | Total QALYs by strategy | Total costs by strategy | Incremental analyses, e.g. ICER (per QALY gained) | Sensitivity analyses outcomes | Author conclusions | Limitations identified by author |
| --- | --- | --- | --- | --- | --- | --- |
| Auguste 2016 [15] | TST (≥5mm):15.516 TST (≥10mm): 15.516 QFT-GIT: 15.517 T-SPOT.TB: 15.523 QFT-GIT +ve then TST (≥5mm): 15.524 QFT-GIT -ve then TST (≥5mm): 15.526 | TST (≥5mm):269.42 TST (≥10mm): 289.31 QFT-GIT: 276.01 T-SPOT.TB: 258.61 QFT-GIT +ve then TST (≥5mm): 280.90 QFT-GIT -ve then TST (≥5mm): 318.26 | TST (≥5mm): Dominated TST (≥10mm): Dominated QFT-GIT: Dominated T-SPOT.TB: NR QFT-GIT +ve then TST (≥5mm): 10,402.63 (vs QFT-GIT) QFT-GIT -ve then TST (≥5mm): 18,746.01 (vs T-SPOT.TB) | Univariate When test sensitivity was equal or higher than base case, the QFT-GIT -ve followed by TST (≥5mm) strategy was consistently the most cost-effective strategy at a WTP threshold of £20,000 per QALY gained In scenarios where the relative importance of test specificity was increased, QFT-GIT often became the most cost-effective strategy PSA At a WTP threshold of £20,000 per QALY gained QFT-GIT -ve followed by TST (≥5mm) is cost effective in the highest proportion of simulations (approx. 50%) | In the immunocompromised population , based on the limited evidence available, QFT-GIT negative followed by TST (≥5mm) was the most cost effective strategy for diagnosing LTBI that progresses to active TB | Evidence available is limited (particularly in terms of inconsistent performance of tests in high- compared with low-incidence TB settings) the prospective assessment of progression to active TB for those at high risk the relative benefits of two- compared with one-step testing with different tests improved classification of people at high and low risk of LTBI |
| Kowada 2019 [31] (Kidney) | Vaccinated (non-vaccinated) No screening: 2.558 (2.558) QFT: 3.026 (3.005) TSPOT: 3.022 (3.026) TST:2.951 (3.022) | Vaccinated (non-vaccinated) No screening: 1001 (1001) QFT: 5679 (5604) TSPOT: 5738 (5679) TST:8049 (5738) | Vaccinated (non-vaccinated) No screening: - (-) QFT: 9990 (10,293) TSPOT: Dominated (9990) TST: Dominated (Dominated) | Cost-effectiveness was not sensitive to BCG vaccination rate, and the costs of screening tests and treatment. Cost effectiveness was sensitive to the sensitivities and specificities of QFT and TSPOT at a WTP of US$100,000 per QALY gained | TB screening using IGRA and individualised TB risk assessment and follow-up monitoring of drug toxicity during LTBI treatment is recommended for solid organ transplantation, on the basis of the benefits and costs | Scarce data of diagnostic accuracies of ITRAs and TST in solid organ transplant recipients Completion of LTBI treatment was difficult due to Aes, the interactions with immunosuppressive drugs and the immunocompromised states of the transplant recipients Risk of donor derived TB is not considered in the study There is no complete TB data on what is happening in the world of human cell, tissue and organ transplantation Cost of education and cost of maintaining IGRAs and labour not included in the model TB risk factors (history of TB, TB exposures, HIV infection, country of origin, higher intensity immunosuppression, diabetes mellitus and increase recipient age) not considered in the model Different costs and medical systems so results may not be generalisable to other countries |
| Kowada 2019 [31] (Liver) | Vaccinated (non-vaccinated) No screening: 1.785 (1.785) QFT: 2.365 (2.350) TSPOT: 2.359 (2.365) TST:2.294 (2.359) | Vaccinated (non-vaccinated) No screening: 936 (936) QFT: 5914 (5566) TSPOT: 5928 (5914) TST:7804 (5928) | Vaccinated (non-vaccinated) No screening: - (-) QFT: 8583 (8192) TSPOT: Dominated (23,566) TST: Dominated (Dominated) | Cost-effectiveness was not sensitive to BCG vaccination rate, and the costs of screening tests and treatment. Cost effectiveness was sensitive to the sensitivities and specificities of QFT and TSPOT at a WTP of US$100,000 per QALY gained | TB screening using IGRA and individualised TB risk assessment and follow-up monitoring of drug toxicity during LTBI treatment is recommended for solid organ transplantation, on the basis of the benefits and costs | Scarce data of diagnostic accuracies of ITRAs and TST in solid organ transplant recipients Completion of LTBI treatment was difficult due to Aes, the interactions with immunosuppressive drugs and the immunocompromised states of the transplant recipients Risk of donor derived TB is not considered in the study There is no complete TB data on what is happening in the world of human cell, tissue and organ transplantation Cost of education and cost of maintaining IGRAs and labour not included in the model TB risk factors (history of TB, TB exposures, HIV infection, country of origin, higher intensity immunosuppression, diabetes mellitus and increase recipient age) not considered in the model Different costs and medical systems so results may not be generalisable to other countries |
| Kowada 2019 [31] (Lung) | Vaccinated (non-vaccinated) No screening: 3.232 (3.232) TSPOT: 3.756 (3.743) QFT: 3.761 (3.756) TST:3.698 (3.761) | Vaccinated (non-vaccinated) No screening: 1559 (1559) TSPOT: 6072 (5904) QFT: 6092 (6072) TST:7981 (6092) | Vaccinated (non-vaccinated) No screening: - (-) TSPOT: 8621 (8508) QFT: 8580 (13,172) TST: Dominated (10,683) | Cost-effectiveness was not sensitive to BCG vaccination rate, and the costs of screening tests and treatment. Cost effectiveness was sensitive to the sensitivities and specificities of QFT and TSPOT at a WTP of US$100,000 per QALY gained | TB screening using IGRA and individualised TB risk assessment and follow-up monitoring of drug toxicity during LTBI treatment is recommended for solid organ transplantation, on the basis of the benefits and costs | Scarce data of diagnostic accuracies of ITRAs and TST in solid organ transplant recipients Completion of LTBI treatment was difficult due to Aes, the interactions with immunosuppressive drugs and the immunocompromised states of the transplant recipients Risk of donor derived TB is not considered in the study There is no complete TB data on what is happening in the world of human cell, tissue and organ transplantation Cost of education and cost of maintaining IGRAs and labour not included in the model TB risk factors (history of TB, TB exposures, HIV infection, country of origin, higher intensity immunosuppression, diabetes mellitus and increase recipient age) not considered in the model Different costs and medical systems so results may not be generalisable to other countries |
| Laskin 2013 [32] | Primary model No screen: 29.33559 Questionnaire/TST if +: 29.33555 Universal TST: 29.33471  Secondary model No screen: 29.33559 Questionnaire/IGRA if +: 29.33560 Questionnaire/TST if +:29.33555 TST/IGRA if +: 29.33558 Universal IGRA: 29.33553 Universal TST: 29.33471 | Primary model No screen: 2,200.96 Questionnaire/TST if +: 2,218.20 Universal TST: 2,480.97  Secondary model No screen: 2,200.96 Questionnaire/IGRA if +: 2,208.01 Questionnaire/TST if +: 2,218.20 TST/IGRA if +: 2,272.43 Universal IGRA: 2,304.89 Universal TST: 2,480.97 | Primary model No screen: - Questionnaire/TST if +: Dominated Universal TST: Dominated  Secondary model No screen: - Questionnaire/IGRA if +: 705,000 Questionnaire/TST if +: Dominated TST/IGRA if +: Dominated Universal IGRA: Dominated Universal TST: Dominated | For targeted screening with a questionnaire followed by IGRA testing if positive results were sensitive to LTBI prevalence and the sensitivity, specificity and cost of the IGRA test | Prior to starting steroid therapy, only patients in areas with a high prevalence of LTBI will benefit from universal TST. More evidence required, but IGRA may become a component of cost-effective screening protocols in populations with a higher burden of LTBI | There is no established gold standard for the diagnosis of LTBI Results of any modelling are limited by the accuracy of the data inputs |
| Linas 2016 [7, 8] | Discounted quality adjusted life months presented in table No screening: 181.66 TST: 181.67 IGRA: 181.67 | Adults No screening: $230,700 TST: $230,750 IGRA: $230,760 | IGRA has an ICER per QALY gained of $286,100 per QALY gained compared to no testing. | ICERs were sensitive to rates of reactivation (lower rates resulted in higher ICERs) and higher costs of IGRA testing (above $64). Other parameters were not found to significantly alter ICERs | LBTI screening would be cost effective in some high risk groups in the USA but not for those on immunosuppressive medications | True activation rate of TB unknown, no gold standard test for TB, only direct medical costs included |
| Van der Have 2014 [33] | TST/CXR: 26.5138 TST/CXR/QFT: 26.5296 | TST/CXR: €44.8 TST/CXR/QFT: €44.9 | €64,340 per QALY gained with TST/CXR/IGRA compared to TST/CXR | Model was insensitive to reactivation rates but adding IGRA became cost effective if prevalence of latent TB increased to 12% or TST+/IGRA- rate increased above 20% | Extensive TB screening and HBV screening are not cost-effective compared with conventional TB screening and no HBV screening, respectively. However, when targeted at high-risk patient groups, these screening strategies are likely to become cost-effective. | Base case derived from literature with heterogenous populations and in some cases based on expert option that may not reflect reality. Vaccination strategies were not considered |

Key: 3HP - once-weekly isoniazid- rifapentine for 12 weeks; 4R – 4 months rafimpin; 6H - 6 months isoniazid monotherapy; BCG - Bacillus Calmette–Guérin; CXR – Chest X-ray; IGRA - Interferon Gamma Release Assay; INH – isoniazid; LTBI – Latent tuberculosis infection; MDR – multi drug resistant; NR – Not recorded; QFT – QuantiFERON; RIF – rafimpin; TSPOT - T-Spot TB test; TST – Tuberculin Skin Test; ICER – Incremental Cost-Effectiveness Ratios; PSA – Probabilistic Sensitivity Analysis; QALY – Quality Adjusted Life Year

# APPENDIX F

# Included studies full extraction tables: Healthcare workers

Table F.1 Healthcare workers: Population information and tests considered

| Author/ Trial ID | Country/region | Description of population | Tests assessed | Prevalence rate of LTBI | BCG vaccination rate | TB activation rate | Secondary transmission rate |
| --- | --- | --- | --- | --- | --- | --- | --- |
| Eralp 2012 [34] | UK | Healthcare workers aged 20-30 | TST IGRA TST+IGRA  (X-ray used to confirm +ve test result) | Prevalence of LTBI: 0.035 (0.035-0.35) Prevalence of TB:0.0001 (0.0001-0.001) | 0.528 | Probability of developing TB from LTBI: 20-35 years: 0.0048 36-55 years: 0.0036 56-70 years: 0.0015 | NR |
| Kowada 2011 [35] | Japan (assumed) | Hypothetical cohort of 40 year old health care workers on a TB ward | Annual QFT alone vs an initial QFT followed by annual CXR | NR | NR | NR | NR |
| Kowada 2015 [20] | Japan | Recent arrivals | For LTBI: TST, QFT For active TB: CXR | Base case (range for SA) 0.0237 (0.0150-0.0345) | NR | Proportion still infected post-LTBI treatment: 0.345 Average number of secondary cases from one index case: 0.2 (0.1-0.3) Average delay from infection to activation (secondary cases): 2.88 | Probability of successful TB treatment: 0.39 (0.1-0.6) Probability of recurrence of active TB after treatment: 0.035 (0.02-0.05) |
| Png 2019 [36] | Tertiary care hospital in Singapore | New and existing HCWs (HCWs with prior history of TB or LTBI were excluded) | NR | Prevalence of LTBI International: 0.63 (0.33-0.79) Singaporean: 0.24 (0.04-0.46) High-risk: 0.3 (0.225-0.375) Low-risk: 0.04 (0.03-0.05)  Prevalence of TB International: 0.00089 (0.00067-0.00395) Singaporean: 0.00040 (0.00035-0.00045) | NR | Annual risk  Develop LTBI High-risk: 0.03 (0.0225-0.0375) Low risk: 0.003 (0.00225-0.01)  Develop TB High risk: 0.0057 (0.004275-0.007125) Low risk: 0.023 (0.001725-0.004) | NR |

Key: 3HP - once-weekly isoniazid- rifapentine for 12 weeks; 4R – 4 months rafimpin; 6H - 6 months isoniazid monotherapy; BCG - Bacillus Calmette–Guérin; CXR – Chest X-ray; IGRA - Interferon Gamma Release Assay; INH – isoniazid; LTBI – Latent tuberculosis infection; MDR – multi drug resistant; NR – Not recorded; QFT – QuantiFERON; RIF – rafimpin; TSPOT - T-Spot TB test; TST – Tuberculin Skin Test

Table F.2 Healthcare workers: Modelling methods

| Author/ Trial ID | Model type | Time horizon | Health states | Perspective | Discounting |
| --- | --- | --- | --- | --- | --- |
| Eralp 2012 [34] | Decision tree in Markov chains | 20 years | Decision tree Test result  Decision tree  CXR if +ve  LTBI treatment adherence w/wout complication  Markov heath states active TB LTBI LTBI with treatment Death Healthy Healthy with unnecessary treatment for LTBI Hepatitis Hepatitis and TB Hepatitis and LTBI | NHS and societal | Future costs discounted at 5% per annum (not clear if benefits discounted) |
| Kowada 2011 [35] | Decision tree followed by a Markov model. | 20 years | Decision tree Test result  CXR if +ve  LTBI treatment adherence w/wout complication   Markov model Healthy (no TB and no LTBI) LTBI TB Dead | NR | 3% for costs and benefits |
| Kowada 2015 [20] | Decision tree followed by a Markov model. | Up to age 60 years | Decision tree Test result  CXR if +ve  LTBI treatment adherence w/wout complication   Markov model Healthy (no TB and no LTBI) LTBI TB Dead | Hospital payer | 3% per year |
| Png 2019 [36] | Decision tree | 3 years | NR In supplement but supplement not available | Hospital perspective | 3% for costs and outcomes |

Key: 3HP - once-weekly isoniazid- rifapentine for 12 weeks; 4R – 4 months rafimpin; 6H - 6 months isoniazid monotherapy; BCG - Bacillus Calmette–Guérin; CXR – Chest X-ray; IGRA - Interferon Gamma Release Assay; INH – isoniazid; LTBI – Latent tuberculosis infection; MDR – multi drug resistant; NR – Not recorded; QFT – QuantiFERON; RIF – rafimpin; TSPOT - T-Spot TB test; TST – Tuberculin Skin Test

Table F.3 Healthcare workers: Cost and utility parameter values

| Author/ Trial ID | Sources of utility values | Utility values used in model (mean (SD)) | Cost year | Currency | Source of test costs | Test costs in model | Sources of treatment costs | Treatment costs in model | Sources of other costs | Other costs in model |
| --- | --- | --- | --- | --- | --- | --- | --- | --- | --- | --- |
| Eralp 2012 [34] | NR | NR | NR Source NICE CG117, 2011 but this guideline has been superseded by NG33 | GB£ | NICE CG117 | TST: £16 IGRA: £44.78 Chest radiograph: £28 | Cambridge TB Service 2010 NHS National Tariff | TB treatment: £1637 (0.5-2 times) LTBI treatment:£647 (0.5 - 2 times) Hepatitis treatment: £640 (0.5-2 times) | Cambridge TB Service 2010 NHS Pay 2/2010 | Contact tracing: £426 (0.5-2 times)  Time to attend for TB treatment: £662 (0.5-2 times) Time to attend for contact tracing: £95 (0.5-2 times) Time to attend for LTBI treatment: £172 (0.5-2 times) Time for hepatitis treatment: £114 (0.5-2 times) |
| Kowada 2011 [35] | NR | NR | NR | Dollars | NR | NR | NR | NR | NR | NR |
| Kowada 2015 [20] | Kowada 2011 [43] | Non-LTBI: 1 LTBI taking no chemoprophylaxis: 1 LTBI taking chemoprophylaxis without complications: 0.99 LTBI taking chemoprophylaxis with liver dysfunction: 0.85 Active TB during treatment and before: 0.8 Dead: 0 | 2012 | US$ | Japanese medical insurance reimbursement table | QFT: 60.6 (30.3-120.2) T-SPOT: 60.6 (30.3-120.2) TST: 15.4 (7.7-30.8) CXR: 36.3 (18.2-61.6) | Unclear (possibly previous Kowada publication) | INH chemoprophylaxis for 6 months: 515.7 (257.9-1035.4) Treatment of TB for 6 months: 14,879 (7440-29,758) | Unclear | Smears and cultures of sputum examination: 69.9 (35.0-139.8) Treatment of INH-induced hepatitis by prophylaxis: 11,903 (5951.5-23,806) |
| Png 2019 [36] | Dobler et al 2015 | During 6-month INH treatment: 0.975 (0.85-1.00) INH-induced hepatitis: 0.667 (0.4-0.8) During treatment of active TB: 0.827 (0.5-0.98) | 2016 | Singaporean dollars converted to 2016 US$ | National University Hospital | Screening programme: 4 (2-8) QFT-G: 72 (36-144) | National University Hospital | Treatment of INH-induced hepatitis: 11,607 Treatment of active TB: 12,305 (6,153-19,688) | NR | NR |

Key: 3HP - once-weekly isoniazid- rifapentine for 12 weeks; 4R – 4 months rafimpin; 6H - 6 months isoniazid monotherapy; BCG - Bacillus Calmette–Guérin; CXR – Chest X-ray; IGRA - Interferon Gamma Release Assay; INH – isoniazid; LTBI – Latent tuberculosis infection; MDR – multi drug resistant; NR – Not recorded; QFT – QuantiFERON; RIF – rafimpin; TSPOT - T-Spot TB test; TST – Tuberculin Skin Test

Table F.4 Healthcare workers: Sensitivity and specificity of LTBI tests

| Author/ Trial ID | Source of test accuracy | Sensitivity | | | | | Specificity | | | | |
| --- | --- | --- | --- | --- | --- | --- | --- | --- | --- | --- | --- |
|  |  | *IGRA* | *QFT* | *TSPOT* | *TST* | *CXR* | *IGRA* | *QFT* | *TSPOT* | *TST* | *CXR* |
| Eralp 2012 [34] | Literature Dosanjh 2008 Menzies 2007 Diel 2011 Pai 2008 | 0.90 (0.82-0.98)  Probability a second IGRA is required: 0.0343 (0.15-0.15) | NR | NR | 0.70 (0.65-0.74)  Probability a second TST is placed: 0.1737 (0.025-0.25) | NR | 0.98 (0.90-0.99) | NR | NR | 0.66 (0.46-0.86) | NR |
| Kowada 2011 [35] | NR | NR | NR | NR | NR | NR | NR | NR | NR | NR | NR |
| Kowada 2015 [20] | NR | NR | 0.84 (0.81-0.87) | 0.89 (0.86-0.91) | 0.77 (0.71-0.82) | 0.70 (0.59-0.82) | NR | 0.99 (0.98-1.00) | 0.98 (0.94-0.99) | In BCG vaccinated HCWs: 0.59 (0.46-0.73) In non-BCG vaccinated HCWs: 0.97 (0.95-0.99) | 0.6 (0.52-0.63) |
| Png 2019 [36] | Assumption | NR | 100% (assumed) | NR | NR | NR | NR | 100% (assumed) | NR | NR | NR |

Key: 3HP - once-weekly isoniazid- rifapentine for 12 weeks; 4R – 4 months rafimpin; 6H - 6 months isoniazid monotherapy; BCG - Bacillus Calmette–Guérin; CXR – Chest X-ray; IGRA - Interferon Gamma Release Assay; INH – isoniazid; LTBI – Latent tuberculosis infection; MDR – multi drug resistant; NR – Not recorded; QFT – QuantiFERON; RIF – rafimpin; TSPOT - T-Spot TB test; TST – Tuberculin Skin Test

Table F.5 Healthcare workers: Treatments for LTBI

| Author/ Trial ID | LTBI treatments considered | Proportion of patients starting treatment | Efficacy of treatment | Percentage maintaining treatment | INH related hepatitis | INH related deaths | Percentage with drug resistant TB | Percentage with mulit-drug resistant TB | TB death rates |
| --- | --- | --- | --- | --- | --- | --- | --- | --- | --- |
| Eralp 2012 [34] | INH+RIF | NR | Efficacy of LTBI treatment: 0.65 | NR | Risk of hepatitis caused by treatment: 0.0177 (0.0177-0.177) | Probability of death due to hepatitis: 0 | NR | NR | Probability of death due to TB: 0.018 (0.018-0.18) Probability of all causes of death: 0.0045 (0.0045-0.045) |
| Kowada 2011 [35] | NR | NR | NR | NR | NR | NR | NR | NR | NR |
| Kowada 2015 [20] | INH | NR | Probability of recurrence of active TB after treatment: 0.035 (0.02-0.05) | Adherence rate of standard 6 month INH chemoprophylaxis protocol for IGRA: 0.8 (0-1) Adherence rate of standard 6 month INH chemoprophylaxis protocol of TST: 0.003 (0.001-0.02) | Probability of INH-induced hepatitis by INH prophylaxis: 0.003 (0.001-0.02) | NR | NR | NR | NR |
| Png 2019 [36] | INH | INH treatment accepted by HCW: 0.64 (0.375-0.85) | Proportion still infected at end of treatment: 0.35 | NR | INH induced hepatitis: 0.003 (0.001-0.02) Hepatitis resolved after INH treatment: 0.95 (0.8-0.99) | NR | NR | NR | NR |

Key: 3HP - once-weekly isoniazid- rifapentine for 12 weeks; 4R – 4 months rafimpin; 6H - 6 months isoniazid monotherapy; BCG - Bacillus Calmette–Guérin; CXR – Chest X-ray; IGRA - Interferon Gamma Release Assay; INH – isoniazid; LTBI – Latent tuberculosis infection; MDR – multi drug resistant; NR – Not recorded; QFT – QuantiFERON; RIF – rafimpin; TSPOT - T-Spot TB test; TST – Tuberculin Skin Test

Table F.6 Healthcare workers: Results

| Author/ Trial ID | Total QALYs by strategy | Total costs by strategy | Incremental analyses, e.g. ICER (per QALY gained) | Sensitivity analyses outcomes | Author conclusions | Limitations identified by author |
| --- | --- | --- | --- | --- | --- | --- |
| Eralp 2012 [34] | **Measured as life years gained** Base case IGRA+TST: 19.07569 IGRA: 19.07714 TST: 19.07088  Market costs IGRA+TST: 19.0757 IGRA: 19.0771 TST: 19.0709 | Base case (£) IGRA+TST: 76.60 IGRA: 99.52 TST: 333.42  Market costs (£) IGRA+TST: 127.13 IGRA: 146.29 TST: 367.45 | Base case (£) IGRA+TST: 0 IGRA: 15,757 TST: -37,358 (Dominated)  Market costs (£) IGRA+TST: 0 IGRA: 13,1173 TST: 35,324 (Dominated) | Findings are robust for wide differences in disease and test parameters (including increasing IGRA cost x3) | Incremental costs per healthy life year gained (a conservative estimate of benefit) are in the £20,000-£30,000 range across wide differences in disease and test parameters. Health gains justify IGRA costs even when IGRA costs 3 times TST cost | Neither TST or IGRA differentiate latent from active TB - the specificity of IGRA is inferred from studies in populations at low risk of TB |
| Kowada 2011 [35] | QFT alone: 16.36 QALYs QFT/CXR: 16.35 QALYs | QFT alone: $2557.11 QFT/CXR: $2940.09 | QFT alone dominated QFT/CXR (more effective and less costly) | At a WTP threshold of $50,000 QFT was more cost effective than QFT/CXR when the prevalence of LTBI in 49 year olds was >0.065 and when QFT reversion rate was >0.28  QFT was less cost-effective than QFT/CXR when incidence of new cases of LTBI was >0.0093  PSA (10,000) trials showed that when compared with QFT/CXR, QFT was cost-effective in all simulations | Annual QFT alone is more effective and less costly than initial QFT followed by annual CXR. | NR |
| Kowada 2015 [20] | At time of employment TST/QFT: 21.065 QFT: 21.071 TST/T-SPOT: 21.057 T-SPOT: 21.014 TST: 20.380 CXR: 19.649  HCWs on a high-risk ward TST/QFT: 20.899 TST/T-SPOT: 20.886 QFT: 20.968 T-SPOT:20.766 TST: 20.176 CXR: 19.395 | At time of employment TST/QFT: 293.76 QFT: 334.91 TST/T-SPOT:380.84  T-SPOT: 406.50 TST: 2623.32 CXR: 6207.42  HCWs on a high-risk ward TST/QFT: 824.23 TST/T-SPOT: 1044.39 QFT: 1050.32 T-SPOT: 1142.99 TST: 2932.33 CXR: 6728.85 | At time of employment TST/QFT: - QFT: 6868.97 TST/T-SPOT: Dominated T-SPOT: Dominated TST: Dominated CXR: Dominated  HCWs on a high-risk ward TST/QFT: - TST/T-SPOT: Dominated QFT: 3262.61 T-SPOT: Dominated TST: Dominated CXR: Dominated | Cost-effectiveness was sensitive to LTBI rate and BCG vaccination rate. TST followed by QFT was more cost effective than QFT when the LTBI rate was <0.026 at the time of employment and <0.08 on a high risk ward Simulations of 10,000 trials demonstrated that QFT was the optimal strategy | The authors found that TST followed by QFT was more cost-effective than QFT when the LTBI rate was <0.026 at the time of employment and <0.08 on a high risk ward. They concluded that systemic TB screening using QFT is cost-effective for screening HCWs and is recommended in low-incidence countries | Higher conversion rates of serial IGRA testing of HCWs not considered Estimates of LTBI and TB rates among HCWs were used based on values from hospital data and assumptions Impact of risk factors not assessed IGRA positivity cannot distinguish between previous infection and recent transmission Birth country, results of prior TST and history of occupational TB exposure not evaluated HCW who accepted LTBI treatment from the group of +ve tests were not determined and followed in the hospital study population Secondary health benefits of reducing transmission to patients not considered Due to different polices and resources for TB screening of HCWs results from this study may not be generalisable to other countries |
| Png 2019 [36] | QALYs per HCW No screening: 2.91 New: 2.98 New international+triennial high risk: 3.03 New international+annual high risk: 3.07 New+triennial universal: 3.09 New+triennial universal+annual high risk: 3.12 New+annual universal: 3.13 | No screening: 46 New: 55 New international+triennial high risk: 53 New international+annual high risk: 70 New+triennial universal: 86 New+triennial universal+annual high risk: 103 New+annual universal: 113 | No screening: - New: 122 New international+triennial high risk: 58 New international+annual high risk: 157 New+triennial universal: 223 New+triennial universal+annual high risk: 275 New+annual universal: 311 | ICERs were most sensitive to the cost of QFT-G but all were below US$50,0000. At lower WTP thresholds, the targeted screening strategy was most likely to be cost effective but if WTP threshold high, universal screening is likely to be most cost-effective | Targeted LTBI screening for HCWs can be highly cost-effective for hospitals in settings similar to Singapore. More inclusive screening strategies (including regular universal screening) can yield better outcomes but are less efficient and may even be unaffordable | NR |

Key: 3HP - once-weekly isoniazid- rifapentine for 12 weeks; 4R – 4 months rafimpin; 6H - 6 months isoniazid monotherapy; BCG - Bacillus Calmette–Guérin; CXR – Chest X-ray; IGRA - Interferon Gamma Release Assay; INH – isoniazid; LTBI – Latent tuberculosis infection; MDR – multi drug resistant; NR – Not recorded; QFT – QuantiFERON; RIF – rafimpin; TSPOT - T-Spot TB test; TST – Tuberculin Skin Test; ICER – Incremental Cost-Effectiveness Ratios; PSA – Probabilistic Sensitivity Analysis; QALY – Quality Adjusted Life Year

# APPENDIX G

# Included studies

# Included studies full extraction tables: Other high-risk populations

Table G.1 Other populations: Population information and tests considered

| Author/ Trial ID | Country/region | Description of population | Tests assessed | Prevalence rate of LTBI | BCG vaccination rate | TB activation rate | Secondary transmission rate |
| --- | --- | --- | --- | --- | --- | --- | --- |
| Kowada 2016 [37] | Japan (assumed) | Mental health patients who are smokers | QFT T-SPOT TST TST followed by QFT TST followed by T-SPOT CXR No screening | NR | NR | NR | NR |
| Kowada 2016 [28] | Japan | Hypothetical cohort of older people | TST  QFT T-SPOT CXR | Prevalence of active TB with chronic kidney disease: 0.0027 (0.0024-0.0032) | NR | Relative risk of reactivation rate: 2.4 (2.1-2.8) | NR |
| Li 2018 [38] | Hong Kong | Older people on entry to residential care | QFT followed by confirmatory CXR and then smear test | LTBI: 0.57 TB: 0.012 | NR | Annual risk: 0.0025 | NR |

Key: 3HP - once-weekly isoniazid- rifapentine for 12 weeks; 4R – 4 months rafimpin; 6H - 6 months isoniazid monotherapy; BCG - Bacillus Calmette–Guérin; CXR – Chest X-ray; IGRA - Interferon Gamma Release Assay; INH – isoniazid; LTBI – Latent tuberculosis infection; MDR – multi drug resistant; NR – Not recorded; QFT – QuantiFERON; RIF – rafimpin; TSPOT - T-Spot TB test; TST – Tuberculin Skin Test

Table G.2 Other populations: Modelling methods

| Author/ Trial ID | Model type | Time horizon | Health states | Perspective | Discounting |
| --- | --- | --- | --- | --- | --- |
| Kowada 2016 [37] | Decision tree followed by a Markov model. | Lifetime | NR | Societal | 3% per year |
| Kowada 2016 [28] | Decision tree followed by a Markov model.  Decision tree followed by a Markov model.  Decision tree followed by a Markov model. | Lifetime (length not reported although it is stated that average time spent living in a nursing home is 4 years) | Decision tree Test result  CXR if +ve  LTBI treatment adherence w/wout complication   Markov model Healthy (no TB and no LTBI) LTBI TB (drug sensitive or MDR) Dead | Societal | 3% for costs and benefits |
| Li 2018 [38] | Decision tree followed by a Markov model | 20 years | **Decision tree nodes** Accept screening LTBI/TB QFT+ve/-ve Conformation CXR +ve/-ve -ve CXR completed/uncompleted IPT  **Markov model** No LTBI TB LTBI Death | Health service provider | 5% pa (costs and benefits) |

Key: 3HP - once-weekly isoniazid- rifapentine for 12 weeks; 4R – 4 months rafimpin; 6H - 6 months isoniazid monotherapy; BCG - Bacillus Calmette–Guérin; CXR – Chest X-ray; IGRA - Interferon Gamma Release Assay; INH – isoniazid; LTBI – Latent tuberculosis infection; MDR – multi drug resistant; NR – Not recorded; QFT – QuantiFERON; RIF – rafimpin; TSPOT - T-Spot TB test; TST – Tuberculin Skin Test; ICER – Incremental Cost-Effectiveness Ratios; PSA – Probabilistic Sensitivity Analysis; QALY – Quality Adjusted Life Year

Table G.3 Other populations: Cost and utility parameter values

| Author/ Trial ID | Sources of utility values | Utility values used in model (mean (SD)) | Cost year | Currency | Source of test costs | Test costs in model | Sources of treatment costs | Treatment costs in model | Sources of other costs | Other costs in model |
| --- | --- | --- | --- | --- | --- | --- | --- | --- | --- | --- |
| Kowada 2016 [37] | NR | NR | NR | NR | NR | NR | NR | NR | NR | NR |
| Kowada 2016 [28] | Guo 2009 Dion 2004 | Well: 1 LTBI taking LTBI treatment without complication: 0.95 LTBI taking LTBI treatment with liver dysfunction: 0.85 DS-TB during treatment and before: 0.8 MDR-TB during treatment and before: 0.58 Dead: 0 | 2014 | US$ | Medical insurance re-imbursement data WHO guidelines Wage structure survey | QFT: 59.5 (22.5-97.1) T-SPOT: 59.5 (22.5-97.1) TST: 15.1 (10.9-31.5) CXR: 35.6 (17.8-71.2) | Literature | 9H chemoprophylaxis: 1219.3 (390.2-1817.2) Treatment of drug-induced hepatitis by chemoprophylaxis: 11,689 (5845-23,378) Treatment of DS-TB for 6 months: 14,612 (7306-29,224) Treatment of MDR-TB: 1,89,457 (94,729-378,914) | Literature | Smears, cultures and drug sensitivity test of sputum examination: 156.8 (78.4-313.6) |
| Li 2018 [38] | Choi 2013, Guo 2008 | Treated active TB disease: 0.85 Untreated active TB disease: 0.7 Drug-related hepatotoxicity: 0.8 | NR | US$ | Assumption (QFT) Government charges (CXR) | QFT: $70 CXR: $11 | Government charges | IPT (6 months): $60 | Government charges | TB hospitalisations per day: $600 First line drugs for TB (6 months): $162 TB follow up treatment (6 months): $293.50 |

Key: 3HP - once-weekly isoniazid- rifapentine for 12 weeks; 4R – 4 months rafimpin; 6H - 6 months isoniazid monotherapy; BCG - Bacillus Calmette–Guérin; CXR – Chest X-ray; IGRA - Interferon Gamma Release Assay; INH – isoniazid; LTBI – Latent tuberculosis infection; MDR – multi drug resistant; NR – Not recorded; QFT – QuantiFERON; RIF – rafimpin; TSPOT - T-Spot TB test; TST – Tuberculin Skin Test

Table G.4 Other populations: Sensitivity and specificity of LTBI tests

| Author/ Trial ID | Source of test accuracy | Sensitivity | | | | | Specificity | | | | |
| --- | --- | --- | --- | --- | --- | --- | --- | --- | --- | --- | --- |
|  |  | *IGRA* | *QFT* | *TSPOT* | *TST* | *CXR* | *IGRA* | *QFT* | *TSPOT* | *TST* | *CXR* |
| Kowada 2016 [37] | TST  QFT T-SPOT CXR | NR | NR | NR | NR | NR | NR | NR | NR | NR | NR |
| Kowada 2016 [28] | TST  QFT T-SPOT CXR | QFT sensitivity: Diel 2010 QFT specificity: Diel 2011  TSPOT sensitivity: Diel 2010 TSPOT specificity: Diel 2011  TST sensitivity: Pai 2008 TST specificity: Pai 2008  CXR sensitivity and specificity: Tattevin 1999, Cohen 1996 | NR | LTBI:0.84 (95% CI: 0.81-0.87) Active TB: 0.8 (95% CI: 0.75-0.84) | LTBI:0.89 (95% cI: 0.86-0.91) Active TB: 0.81 (95% CI: 0.78-0.84) | For LTBI: 0.77 (95% CI: 0.71-0.82) | Active TB: 0.70 (0.59-0.82) | NR | LTBI: 0.99 (95% CI: 0.98-1.00) Active TB: 0.79 (95% CI: 0.75-0.82) | LTBI: 0.98 (95% CI: 0.94-0.99) Active TB: 0.59 (95% CI: 0.56-0.62) | **LTBI** BCG vaccinated: 0.59 (95% CI: 0.46-0.73) Non-BCG vaccinated: 0.97 (95% CI: 0.95-0.99) |
| Li 2018 [38] | QFT sensitivity/specificity: Diel 2010  CXR sensitivity and specificity: WHO, Hjaltado ́ttir 2011, Barnes 1988, Dasgupta 2005 | NR | 0.84 (with 60% accepting screening) | NR | NR | 0.7 | NR | 0.99 | NR | NR | 0.6 |

Key: 3HP - once-weekly isoniazid- rifapentine for 12 weeks; 4R – 4 months rafimpin; 6H - 6 months isoniazid monotherapy; BCG - Bacillus Calmette–Guérin; CXR – Chest X-ray; IGRA - Interferon Gamma Release Assay; INH – isoniazid; LTBI – Latent tuberculosis infection; MDR – multi drug resistant; NR – Not recorded; QFT – QuantiFERON; RIF – rafimpin; TSPOT - T-Spot TB test; TST – Tuberculin Skin Test

Table G.5 Other populations: Treatments for LTBI

| Author/ Trial ID | LTBI treatments considered | Proportion of patients starting treatment | Efficacy of treatment | Percentage maintaining treatment | INH related hepatitis | INH related deaths | Percentage with drug resistant TB | Percentage with mulit-drug resistant TB | TB death rates |
| --- | --- | --- | --- | --- | --- | --- | --- | --- | --- |
| Kowada 2016 [37] | NR | NR | NR | NR | NR | NR | NR | NR | NR |
| Kowada 2016 [28] | INH | Efficacy: 0.8 | Adherence rate of 9H chemoprophylaxis: 0.365 (0-1) | Probability of drug-related hepatotoxicity by 9H chemoprophylaxis: 0.021 (0.01-0.04) | NR | 0.60% | NR | TB mortality: 0.366 (0.2-0.5) | NR |
| Li 2018 [38] | INH | NR | Efficacy 0.85 | 0.8 | 0.017 | NR | NR | NR | **Treated patients** 65-69: 0.028 70-74: 0.069 75-79: 0.068 80-84: 0.084  **Untreated patients** 0.113 (untreated smear positive) 0.022 (untreated smear negative) |

Key: 3HP - once-weekly isoniazid- rifapentine for 12 weeks; 4R – 4 months rafimpin; 6H - 6 months isoniazid monotherapy; BCG - Bacillus Calmette–Guérin; CXR – Chest X-ray; IGRA - Interferon Gamma Release Assay; INH – isoniazid; LTBI – Latent tuberculosis infection; MDR – multi drug resistant; NR – Not recorded; QFT – QuantiFERON; RIF – rafimpin; TSPOT - T-Spot TB test; TST – Tuberculin Skin Test

Table G.6 Other populations: Results

| Author/ Trial ID | Total QALYs by strategy | Total costs by strategy | Incremental analyses, e.g. ICER (per QALY gained) | Sensitivity analyses outcomes | Author conclusions | Limitations identified by author |
| --- | --- | --- | --- | --- | --- | --- |
| Kowada 2016 [37] | NR | NR | QFT: - TST: Dominated CXR: Dominated | Results were sensitive to the BCG vaccination rate (TST more CE than QFT at WTP of US$50,000 when rate of BCG vaccination was ≤0.18). PSA (10,000 trials) showed that QFT was more CE than TST or CXR for all trials at all WTP thresholds | TB screening using an IGRA for mental patients is recommended on the basis of the cost-effectiveness, especially with tobacco smoking | NR |
| Kowada 2016 [28] | No screening: 1.32143 QFT: 4.36405 T-SPOT: 4.36356 TST/QFT: 4.36619 TST/T-SPOT: 4.36588 TST: 4.36100 CXR: 4.37175 | No screening: 221.4 QFT: 413.5 T-SPOT: 428.9 TST/QFT: 499.7 TST/T-SPOT: 508.6 TST: 669.7 CXR: 6701.4 | No screening: - QFT: 91.3 T-SPOT: Dominated TST/QFT: 59,129.9 TST/T-SPOT: Dominated TST: Dominated CXR: 908,961.6 | Cost-effectiveness was sensitive to LTBI rate and BCG vaccination rate TST followed by QFT was the more cost-effective than QFT when the LTBI rate was ≥0.35 and when the BCG vaccination rate was <0.57 at a WTP threshold of $50,000 per QALY gained Hepatotoxicity by 9-month INH chemoprophylaxis had little impact on cost-effectiveness | Effective LTBI screening using IGRA is recommended to prevent TB transmission not only in nursing homes but also in local communities in low-incidence countries | Sensitivities and specifities of TB screening kits (IGRA and TST) were obtained from meta-analyses of immunocompetent individuals, not older people with waning immunity There is little data on LTBI rates using IGRAs in nursing home residents Harm from radiation exposure by repeating CXR not considered Use of rifapentine plus isoniazid for 3 months (higher treatment completion rate) was not considered as a chemoprophylaxis regimen Further epidemiology of TB in elderly is needed to make a convincing case for TB policy change No method for diagnosing whether LTBI differentiates first infection with TB from reinfection Few epidemiological studies of TB outbreaks in nursing homes Different costs and medical systems between countries may impact on generalisability of findings |
| Li 2018 [38] | QFT: 22.87049  No screening: 22.85452 CXR: 22.85453 | QFT: 262.85 No screening: 448.38  CXR: 543.50 | $19,712 per QALY gained with QFT vs no testing | Results were insensitive to all values varied in deterministic sensitivity analysis and screening for LTBI remained most cost effective strategy in majority of iterations of PSA at a WTP of $50,000/QALY | Using an IGRA for TB screening of high-risk HIV +ve pregnant women in low TB incidence countries is recommended on the basis of cost effectiveness | Estimates of each of the variables in the model were obtained from studies conducted in numerous countries The sensitivity and specificity estimates for IGRAs in HIV+ve pregnant women were obtained from meta-analyses of published literature and assumptions gut little is known on effect of pregnancy on sensitivity and specificity of IGRAs and TST Prevention of TB spread by pregnant women is a more urgent problem in developing countries than in developed countries Routine use of chest radiography in the absence of clear symptoms of active TB may be unwarranted and raise ethical considerations Women may be less likely than men to submit good-quality sputum The use of chemoprophylaxis for pregnant women is controversial There is little data of TB incidence and LTBI prevalence in HIV +ve pregnant women There is no data of TB risk in pregnancy trimester Different countries have different policies and resources for TB screening - generalisability of results unclear Costs of the side effects of MDR-TB therapy was not included in the model Use of chemoprophylaxis for pregnant women is controversial |

Key: 3HP - once-weekly isoniazid- rifapentine for 12 weeks; 4R – 4 months rafimpin; 6H - 6 months isoniazid monotherapy; BCG - Bacillus Calmette–Guérin; CXR – Chest X-ray; IGRA - Interferon Gamma Release Assay; INH – isoniazid; LTBI – Latent tuberculosis infection; MDR – multi drug resistant; NR – Not recorded; QFT – QuantiFERON; RIF – rafimpin; TSPOT - T-Spot TB test; TST – Tuberculin Skin Test; ICER – Incremental Cost-Effectiveness Ratios; PSA – Probabilistic Sensitivity Analysis; QALY – Quality Adjusted Life Year

# APPENDIX H Included studies

# Included studies full extraction tables: Children

Table H.1 Children: Population information and tests considered

| Author/ Trial ID | Country/region | Description of population | Tests assessed | Prevalence rate of LTBI | BCG vaccination rate | TB activation rate | Secondary transmission rate |
| --- | --- | --- | --- | --- | --- | --- | --- |
| Auguste 2016 [15] | UK | Children | TST QFT-GIT T-SPOT.TB CXR | Base case (range for SA) 0.0288 (0.0206-0.0384) | Model not stratified by BCG status | Annualised reactivate rate: 0.013 (0.004-0.025) | Proportion still infected post-LTBI treatment: 0.345 Average number of secondary cases from one index case: 0.2 (0.1-0.3) Average delay from infection to activation (secondary cases): 2.88 |
| Kowada 2012 [39] | Japan | Immunocompetent 16- and 19-year olds | QFT TST CXR | Probability of having TB (differed by 5 year age bands) Age 15: 0.000034 Age 80: 0.000798  Probability of having LTBI (differed by 5 year age bands) Age 15: 0.0078 Age 80: 0.7302  Probability of developing TB from LTBI Age 16-35: 0.0037 Age 36-55: 0.0028 Age 56-80: 0.0015 | NR | Probability of recurrence of active TB after treatment: 0.035 (0.02-0.05) | NR |

Key: 3HP - once-weekly isoniazid- rifapentine for 12 weeks; 4R – 4 months rafimpin; 6H - 6 months isoniazid monotherapy; BCG - Bacillus Calmette–Guérin; CXR – Chest X-ray; IGRA - Interferon Gamma Release Assay; INH – isoniazid; LTBI – Latent tuberculosis infection; MDR – multi drug resistant; NR – Not recorded; QFT – QuantiFERON; RIF – rafimpin; TSPOT - T-Spot TB test; TST – Tuberculin Skin Test

Table H.2 Children: Modelling methods

| Author/ Trial ID | Model type | Time horizon | Health states | Perspective | Discounting |
| --- | --- | --- | --- | --- | --- |
| Auguste 2016 [15] | Decision tree followed by DES | 100 years | **Decision tree**  Determinate/indeterminate result (TST only) +ve/-ve tests in isolation or combination  +ve/-ve CXR Gastric lavage Active TB and treated/no active TB (LTBI) Treatment adherence/non-adherence Accept/refuse LTBI treatment LTBI treatment adherence/non-adherence  **Dynamic transmission model** No LTBI/TB LTBI Active TB Resolved TB Secondary infections Death all causes Death TB | NHS and Personal Social Services | 3.5% costs and outcomes |
| Kowada 2012 [39] | Decision tree followed by a Markov model. | Lifetime (up to age 80 years) | Decision tree Test result  CXR if +ve  LTBI treatment adherence w/wout complication   Markov model Healthy (no TB and no LTBI) LTBI TB Dead | Societal | 3% for costs and benefits |

Key: 3HP - once-weekly isoniazid- rifapentine for 12 weeks; 4R – 4 months rafimpin; 6H - 6 months isoniazid monotherapy; BCG - Bacillus Calmette–Guérin; CXR – Chest X-ray; IGRA - Interferon Gamma Release Assay; INH – isoniazid; LTBI – Latent tuberculosis infection; MDR – multi drug resistant; NR – Not recorded; QFT – QuantiFERON; RIF – rafimpin; TSPOT - T-Spot TB test; TST – Tuberculin Skin Test

Table H.3 Children: Cost and utility parameter values

| Author/ Trial ID | Sources of utility values | Utility values used in model (mean (SD)) | Cost year | Currency | Source of test costs | Test costs in model | Sources of treatment costs | Treatment costs in model | Sources of other costs | Other costs in model |
| --- | --- | --- | --- | --- | --- | --- | --- | --- | --- | --- |
| Auguste 2016 [15] | Kowada | **Decrements** Active TB (while on treatment): 0.15 Treatment for LTBI: 0.001 | 2012/13 | GB£ | Pooran et al and NHS Reference costs 2012/13 | TST: 17.48 QFT-GIT: 48.73 T-SPOT.TB: 59.57 CXR:35.00 | Bothamley et al NHS drug tariff Pareek et al | Adherence to active TB treatment: 5461.12 Non-adherence to active TB treatment: 910.19 Adherence to LTBI treatment: 677.07 Non-adherence to LTBI treatment: 112.85 Treatment of isoniazid-induced hepatitis: 389.51 | NHS Reference Costs 2012/13 | Gastric lavage procedure: 916.00 Sputum examination: 7.00 |
| Kowada 2012 [39] | Tsevat 1988 | Healthy: 1 LTBI: 1 LTBI with chemoprophylaxis (9 months): 0.996 Non-fatal active TB during and before treatment: 0.85 Dead: 0 | 2009 | US$ | Medical insurance re-imbursement Yoshiyama 2000 | QFT: 64.5 (32.3-129) TST: 17.2 (8.6-34.4) CXR: 40.5 (20.3-81) | Yoshiyama 2000 | Chemoprophylaxis by INH for 9 months: 864.3 (432.2-1,728.6) Treatment of INH-induced hepatitis by INH chemoprophylaxis: 13,298 (6,649-26,596) Treatment of TB for 6 months: 16,623 (8311-33,246) | Medical insurance re-imbursement | Smear and culture of sputum examination: 78.1 (39.1-156.2) |

Key: 3HP - once-weekly isoniazid- rifapentine for 12 weeks; 4R – 4 months rafimpin; 6H - 6 months isoniazid monotherapy; BCG - Bacillus Calmette–Guérin; CXR – Chest X-ray; IGRA - Interferon Gamma Release Assay; INH – isoniazid; LTBI – Latent tuberculosis infection; MDR – multi drug resistant; NR – Not recorded; QFT – QuantiFERON; RIF – rafimpin; TSPOT - T-Spot TB test; TST – Tuberculin Skin Test

Table H.4 Children: Sensitivity and specificity of LTBI tests

| Author/ Trial ID | Source of test accuracy | Sensitivity | | | | | Specificity | | | | |
| --- | --- | --- | --- | --- | --- | --- | --- | --- | --- | --- | --- |
|  |  | *IGRA* | *QFT* | *TSPOT* | *TST* | *CXR* | *IGRA* | *QFT* | *TSPOT* | *TST* | *CXR* |
| Auguste 2016 [15] | Meta-analysis of published literature | NR | 0.6884 (0.5856-0.7820) Conditional on positive TST (LTBI arm): 0.6775 (0.4674-0.9233) Conditional on negative TST (LTBI arm): 0.7031 (0.1122-0.9921)  Determinate QFT-GIT: 0.97 | 0.5 (0.0245-0.9764) Determinate T-SPOT. TB: 0.97 | ≥5mm 0.7280 (0.0206-0.0384) ≥10mm: 0.5351 (0.3821-0.6769)  TST read: 0.9400 (0.6-1.00) | For diagnosing active TB: 0.7800 | NR | 0.6103 (0.6030-0.6176) Conditional on positive TST (LTBI arm): 0.3213 (0.3073-0.3353) Conditional on negative TST (LTBI arm): 0.9108 (0.9013-0.9200) | 0.7758 (0.6738-0.8640) | <5mm: 0.4903 (0.4796-0.5008) <10mm: 0.7481 (0.3434-0.7618) | For diagnosing active TB: 0.51 |
| Kowada 2012 [39] | Literature | NR | For LTBI: 0.84 (0.81-0.87) | NR | For LTBI:0.77 (0.71-0.82) | For active TB: 0.7 (0.59-0.82) | NR | For LTBI: 0.99 (0.98-1.0) | NR | BCG-vaccinated for LTBI: 0.59 (0.46-0.73) Non-BCG vaccinated for LTBI: 0.97 (0.95-0.99) | For active TB: 0.60 (0.52-0.63) |

Key: 3HP - once-weekly isoniazid- rifapentine for 12 weeks; 4R – 4 months rafimpin; 6H - 6 months isoniazid monotherapy; BCG - Bacillus Calmette–Guérin; CXR – Chest X-ray; IGRA - Interferon Gamma Release Assay; INH – isoniazid; LTBI – Latent tuberculosis infection; MDR – multi drug resistant; NR – Not recorded; QFT – QuantiFERON; RIF – rafimpin; TSPOT - T-Spot TB test; TST – Tuberculin Skin Test

Table H.5 Children: Treatments for LTBI

| Author/ Trial ID | LTBI treatments considered | Proportion of patients starting treatment | Efficacy of treatment | Percentage maintaining treatment | INH related hepatitis | INH related deaths | Percentage with drug resistant TB | Percentage with mulit-drug resistant TB | TB death rates |
| --- | --- | --- | --- | --- | --- | --- | --- | --- | --- |
| Auguste 2016 [15] | INH | Accepting LTBI treatment: 0.9400 (0.50-1.0) | Proportion still infected at end of treatment: 0.345 | Adherence to LTBI treatment: 0.8--- (0.50-0.90) | 0.0040 (0.001-0.010) | 0.00002 (0.00001-0.0001) | NR | NR | 0.05 (0.025-0.075) |
| Kowada 2012 [39] | INH | NR | Efficacy of LTBI treatment: 0.7 | Adherence rate of standard 9 month INH chemoprophylaxis protocol: 0.8 (0.5-0.9) | 0.003 (0.002-0.005) | NR | NR | NR | Mortality rate for active TB among TB patients (5 year age bands) from age 5: 0.000337 to age 80: 0.195661 |

Key: 3HP - once-weekly isoniazid- rifapentine for 12 weeks; 4R – 4 months rafimpin; 6H - 6 months isoniazid monotherapy; BCG - Bacillus Calmette–Guérin; CXR – Chest X-ray; IGRA - Interferon Gamma Release Assay; INH – isoniazid; LTBI – Latent tuberculosis infection; MDR – multi drug resistant; NR – Not recorded; QFT – QuantiFERON; RIF – rafimpin; TSPOT - T-Spot TB test; TST – Tuberculin Skin Test

| Author/ Trial ID | Total QALYs by strategy | Total costs by strategy | Incremental analyses, e.g. ICER (per QALY gained) | Sensitivity analyses outcomes | Author conclusions | Limitations identified by author |
| --- | --- | --- | --- | --- | --- | --- |
| Auguste 2016 [15] | TST (≥10mm): 19.909 T-SPOT.TB: 19.915 QFT-GIT: 19.917 TST (≥5mm): 19.922 TST (≥5mm) positive followed by QFT-GIT: 19.923 | TST (≥10mm): 300.10 T-SPOT.TB: 400.12 QFT-GIT: 291.13 TST (≥5mm): 298.75 TST (≥5mm) positive followed by QFT-GIT: 353.47 | TST (≥10mm): Dominated T-SPOT.TB: Dominated QFT-GIT: NR TST (≥5mm): 1524 (vs QFT-GIT) TST (≥5mm) positive followed by QFT-GIT: 58,720 (vs TST≥5mm) | **Univariate** In the majority of the scenarios results were similar to base case (TST (≥5mm)-alone strategy was the most cost effective). However, decrease in the prevalence of LTBI, increase in the sensitivity of QFT-GIT and decrease in the sensitivity of the TST all led to strategies involving QFT-GIT becoming cost-effective **PSA** At a WTP threshold of £20,000 per QALY gained TST (≥5mm) is cost effective in the highest proportion of simulations (approx. 50%) | In children, based on the limited evidence available, TST (≥5mm) negative followed by QFT-GIT was the most cost effective strategy for diagnosing LTBI that progresses to active TB | Evidence available is limited (particularly in terms of inconsistent performance of tests in high- compared with low-incidence TB settings) the prospective assessment of progression to active TB for those at high risk the relative benefits of two- compared with one-step testing with different tests improved classification of people at high and low risk of LTBI |
| Kowada 2012 [39] | 16 year olds QFT: reference  TST: -0.00069 CXR:-0.00303  19 year olds QFT: reference TST: -0.00073 CXR: -0.00450 | 16 year olds QFT: 627.89 TST: 943.50 CXR: 7286.24  19 year olds QFT: 646.04 TST: 998.62 CXR:7305.19 | 16 year olds QFT: -  TST: Dominated CXR: Dominated  19 year olds QFT: -  TST: Dominated CXR: Dominated | One way SA: the cost-effectiveness of QFT was not sensitivity to any variables two way SA: No variable was sensitivity. QFT yielded greater benefits at lower cost than TST and CXR at a WTP threshold of US$50,000 PSA (10,000 trials): QFT most cost effective for all iterations | For school-based TB screening, use of QFT provides greater benefits at a lower cost than screening via either TST or CXR, independent of BCG vaccination status | The assumed prevalence of LTBI estimate may be too high Probability of resolving LTBI using INH was not considered Potential psychologic stress caused by attending TB screening was not considered Th prevalence of student groups with high TB infection risks were not included in this analysis |

Table H.6 Children: Results

Key: 3HP - once-weekly isoniazid- rifapentine for 12 weeks; 4R – 4 months rafimpin; 6H - 6 months isoniazid monotherapy; BCG - Bacillus Calmette–Guérin; CXR – Chest X-ray; IGRA - Interferon Gamma Release Assay; INH – isoniazid; LTBI – Latent tuberculosis infection; MDR – multi drug resistant; NR – Not recorded; QFT – QuantiFERON; RIF – rafimpin; TSPOT - T-Spot TB test; TST – Tuberculin Skin Test; ICER – Incremental Cost-Effectiveness Ratios; PSA – Probabilistic Sensitivity Analysis; QALY – Quality Adjusted Life Year

# Appendix I

# Detailed risk of bias assessments

| **Study** | **1. Narrow perspective bias: Was a societal perspective adopted? If not has a different perspective been justified?** | | **2. Inefficient comparator bias: Was the best alternative chosen as comparators?** | | **3. Cost measurement omission bias: Were all costs relevant to the disease and intervention identified?** | | **4. Intermittent data collection bias: Was the resource use measured continuously?** | | **5. Invalid valuation bias: Is the price calculation presented in a detailed manner? Have reference prices been used?** | | **6. Ordinal ICER bias: Have cardinal scales for the outcomes measure in a CEA been used?** | |
| --- | --- | --- | --- | --- | --- | --- | --- | --- | --- | --- | --- | --- |
|  | ***Relevant to study? Yes/No/Partly/Unclear*** | ***Comment - provide brief justification for judgement*** | ***Relevant to study? Yes/No/Partly/Unclear*** | ***Comment - provide brief justification for judgement*** | ***Relevant to study? Yes/No/Partly/Unclear*** | ***Comment - provide brief justification for judgement*** | ***Relevant to study? Yes/No/Partly/Unclear*** | ***Comment - provide brief justification for judgement*** | ***Relevant to study? Yes/No/Partly/Unclear*** | ***Comment - provide brief justification for judgement*** | ***Relevant to study? Yes/No/Partly/Unclear*** | ***Comment - provide brief justification for judgement*** |
| Al Abri 2020 [24] | Yes | Omani healthcare sector | Unclear | Choice not justified | Yes | Test and treatment costs of TB included | No | Model parameter values extracted from literature or Omani data | Yes | Details of costs used provided | Yes | QALYS used |
| Campbell 2017 [16] | Yes | Canadian healthcare system | Unclear | Choice not justified | Yes | Test and treatment costs of TB included | No | Modelling based on published data | Yes | Details of costing provided. Costs sourced from the literature | Yes | QALYS used |
| Campbell 2019 [17] | Yes | Canadian healthcare system | Unclear | Choice not justified | Yes | Test and treatment costs of TB included | No | Modelling based on published data | Yes | Details of costing provided. Costs sourced from the literature | Yes | QALYS used |
| Campbell 2019 [18] | Yes | Canadian healthcare system | Unclear | Choice not justified | Yes | Test and treatment costs of TB included | No | Modelling based on published data | Yes | Details of costing provided. Costs sourced from the literature | Yes | QALYS used |
| Tasillo 2017 [13] | Yes | US healthcare system | Unclear | Choice not justified | Yes | Test and treatment costs of TB included | No | Modelling based on published data | Yes | Details of costing provided. Costs sourced from the literature | Yes | QALYS used |
| Auguste 2016 [2] | Yes | UK health service and PSS | Yes | Developed with clinical input and reported to represent the clinical pathways people would take while being screened for LTBI | Yes | Test and treatment costs of TB included | No | Modelling based on published data | Yes | Details of costing provided. Costs sourced from the literature | Yes | QALYS used |
| Eralp 2012 [40] | Yes | UK health service and time to attend for treatment and contact tracing | Yes | Partial justification provided | Yes | Test and treatment costs included | No | Modelling based on published data | Yes | Only simple costs reported | Yes | Time |
| Png [42] | Yes | Hospital perspective | Unclear | One intervention, seven strategies | Yes | Test and treatment costs included | No | Modelling based on published data | Yes | Hospital costs utilised | Yes | QALYS used |
| Hayama 2017 [34] | Yes | NHS perspective | Unclear | Choice not justified | Unclear | NR | No | Abstract - data sources NR | Yes | NR | Yes | QALYS used |
| Kowada 2011 [41] | Yes | NR | Unclear | Choice not justified | Unclear | Costs NR | Unclear | Letter - data sources NR | Yes | NR | Yes | QALYS used |
| Kowada 2015 [35] | Yes | Public health perspective only | Unclear | Choice not justified | Yes | Test and treatment costs included | No | Modelling based on published data | Yes | Details of costing provided. Costs sourced from the literature | Yes | QALYS used |
| Goodell [15] | Yes | Healthcare | Unclear | Choice not justified | Yes | Test and treatment costs included | No | Modelling based on published data | Yes | Details of costing provided. Costs sourced from the literature | Yes | QALYS used |
| Laskin [38] | Yes | Stated to be societal but only health care costs reported | Unclear | Choice not justified | Yes | Test and treatment costs included | No | Modelling based on published data | Yes | Details of costing provided. Costs sourced from the literature | Yes | QALYS used |
| Kowada 2015 [25] | Yes | Hospital payer | Unclear | Choice not justified | Yes | Test and treatment costs included, although source of treatment costs unclear | No | Modelling based on published data | Yes | Details of costing provided. Costs sourced from the literature | Yes | QALYS used |
| Kowada 2016 [44] | Yes | Reported to be societal but included costs not reported | Unclear | Choice: new vs old | Yes | Disaggregated costs not reported | Unclear | Data sources NR | Yes | NR | Yes | QALYS used |
| Kowada 2016 [33] | Yes | Reported to be societal but appears to be health care | Unclear | Choice not justified | Yes | Test and treatment costs included | No | Modelling based on published data | Yes | Details of costing provided. Costs sourced from published sources | Yes | QALYS used |
| Kowada 2012 [46] | Yes | Reported to be societal but appears to be health care | Unclear | Choice not justified | Yes | Test and treatment costs included | No | Modelling based on published data | Yes | Details of costing provided. Costs sourced from published sources | Yes | QALYS used |
| Kowada 2019 [37] | Yes | Reported to be societal but appears to be health care | Unclear | Choice not justified | Yes | Test and treatment costs included | No | Modelling based on published data | Yes | Details of costing provided. Costs sourced from published sources | Yes | QALYS used |
| Kowada 2011 [49] | Yes | Reported to be societal but appears to be health care | Unclear | Choice not justified | Yes | Test and treatment costs included | No | Modelling based on published data | Yes | Details of costing provided. Costs sourced from published sources | Yes | QALYS used |
| Kowada 2013 [36] | Yes | Reported to be societal but appears to be health care | Unclear | Choice not justified | Yes | Test and treatment costs included | No | Modelling based on published data | Yes | Details of costing provided. Costs sourced from published sources | Yes | QALYS used |
| Kowada 2013 [32] | Yes | Reported to be societal but appears to be health care | Unclear | Choice not justified | Yes | Test and treatment costs included | No | Modelling based on published data | Yes | Details of costing provided. Costs sourced from published sources | Yes | QALYS used |
| Kowada 2014 [22] | Yes | Public health payer perspective | Unclear | Choice not justified | Yes | Test and treatment costs included | No | Modelling based on published data | Yes | Details of costing provided. Costs sourced from published sources | Yes | QALYS used |
| Kowada 2016 [21] | Yes | Societal perspective adopted | Yes | CXR is current standard of care | Yes | Test and treatment costs of TB included | NR | Modelling based upon published data | Yes | Details of costing methodology given with sources provided which appear to be Japanese standard sources for healthcare and labour costs | Yes | QALYS used |
| Linas 2011 [11] | Yes | Health care | Yes | No testing is current paradigm | Yes | Test and treatment costs of TB included | NR | Modelling based upon published data | Unclear | Details of costs used provided but unclear exactly where sourced | Yes | QALYS used |
| Capocci 2016 [173] | Yes | NHS | Unclear | Choice not justified | Unclear | Detail not provided | Unclear | Detail not provided | Unclear | Detail not provided | Yes | QALYS used |
| Jo 2020 [174] | Yes | State healthcare system and QALYs so societal | Yes | No testing is current paradigm | Yes | Test and treatment costs of TB included | NR | Modelling based upon published data | Yes | Details of costs used provided | Yes | QALYS used |
| Abubakar 2018 [20] | Yes | NHS | Yes | Compared to NICE guideline | Yes | Test and treatment costs of TB included | NR | Modelling based upon published data | Yes | Details of costs used provided | Yes | QALYS used |
| Capocci 2020 [31] | Yes | NHS | Yes | Based on guidelines | Unclear | Detail not provided | Unclear | Detail not provided | Yes | Details of costs used provided | Yes | QALYS used |
| Capocci (2016) (a) [175] | Yes | NHS | Unclear | Choice not justified | Unclear | Detail not provided | Unclear | Detail not provided | Unclear | Detail not provided | Yes | QALYS used |
| Capocci 2015 [28] | Yes | NHS | Yes | Based on guidelines | Yes | Test and treatment costs of TB included | Unclear | Detail not provided | Yes | Details of costs used provided | Yes | QALYS used |
| Capocci 2014 [27] | Unclear | Not stated | Yes | Based on guidelines | Unclear | Detail not provided | Unclear | Detail not provided | Yes | Details of costs used provided | Yes | QALYS used |
| Capocci 2012 [26] | Unclear | Not stated | Yes | Based on guidelines | Unclear | Detail not provided | Unclear | Detail not provided | Unclear | Detail not provided | Yes | QALYS used |
| Li 2018 [176] | Yes | Healthcare provider | Yes | No testing is current paradigm | Yes | Test and treatment costs of TB included | Unclear | Detail not provided | Yes | Details of costs used provided | Yes | QALYS used |
| van der Have 2014 [39] | Yes | Third party payer | Yes | TST/CXR is current paradigm | Yes | Test and treatment costs of TB included | Unclear | Detail not provided | Yes | Test and treatment costs of TB included | Yes | QALYS used |

Part 2

| **Study** | **7. Double counting bias: Are variables adequately checked for double-counting?** | | **8. Inappropriate discounting bias: Have discount rates from guidelines been used?** | | **9. Limited sensitivity analysis bias: Have the four principles of uncertainty (methodological, structural, heterogeneity, parameter) been considered in sufficient detail?** | | **10. Sponsor bias: Have sponsorships been disclosed? Is the study protocol freely accessible?** | | **11. Reporting and dissemination bias: Has the study/trial been listed in a trial register? Have all results been reported according to the study protocol?** | | **12. Structural assumptions bias: Is the model structure in line with coherent theory? Do treatment pathways reflect the nature of disease?** | |
| --- | --- | --- | --- | --- | --- | --- | --- | --- | --- | --- | --- | --- |
|  | ***Relevant to study? Yes/No/Partly/Unclear*** | ***Comment - provide brief justification for judgement*** | ***Relevant to study? Yes/No/Partly/Unclear*** | ***Comment - provide brief justification for judgement*** | ***Relevant to study? Yes/No/Partly/Unclear*** | ***Comment - provide brief justification for judgement*** | ***Relevant to study? Yes/No/Partly/Unclear*** | ***Comment - provide brief justification for judgement*** | ***Relevant to study? Yes/No/Partly/Unclear*** | ***Comment - provide brief justification for judgement*** | ***Relevant to study? Yes/No/Partly/Unclear*** | ***Comment - provide brief justification for judgement*** |
| Al Abri 2020 [24] | Unclear | Not discussed in study | Yes | No report of discounting having been carried out | Partly | Only parameter uncertainty explored | Yes | Study sponsored by Qiagen | No | Not a trial | Yes | Decision tree followed by a Markov model |
| Campbell 2017 [16] | Unclear | Not discussed in study | Yes | Rate recommended by the Canadian Agency for Drugs and Technologies in Health | Partly | Only parameter uncertainty explored | Yes | Authors declared that there had been no specific funding for this work | No | Not a trial | Yes | DES |
| Campbell 2019 [17] | Unclear | Not discussed in study | Yes | Discount rate provided but rational for rate not given | Partly | Only parameter uncertainty explored | Yes | Funding has been disclosed | No | Not a trial | Yes | DES |
| Campbell 2019 [18] | Unclear | Not discussed in study | Yes | Rate recommended by the Canadian Agency for Drugs and Technologies in Health | Partly | Only parameter uncertainty explored | Yes | Funding has been disclosed | No | Not a trial | Yes | DES |
| Tasillo 2017 [13] | Unclear | Not discussed in study | Yes | Rationale not provided | Partly | Only parameter uncertainty explored | Yes | Funding has been disclosed | No | Not a trial | Yes | Decision tree followed by a Markov model |
| Auguste 2016 [2] | Unclear | Not discussed in study | Yes | UK accepted rate | Partly | Only parameter uncertainty explored | Yes | Funding has been disclosed | No | Not a trial | Yes | Decision trees followed by DESs |
| Eralp 2012 [40] | Unclear | Not discussed in study | Yes | Not discussed in study | Partly | Only parameter uncertainty explored | Yes | Specified that not funded | No | Not a trial | Yes | Decision tree followed by Markov model |
| Png [42] | Unclear | Not discussed in study | Yes | Commonly used value (3%) used | Partly | Only parameter uncertainty explored | Yes | Specified that financial support was not provided | No | Not a trial | Yes | Decision tree - details not available (link to supplementary material not valid) |
| Hayama 2017 [34] | Unclear | Not discussed in study | Yes | Discount rate provided but rational for rate not given | No | Sensitivity analyses NR (abstract0 | Yes | Funding not reported | No | Not a trial | Yes | Markov decision analytic model - details NR |
| Kowada 2011 [41] | Unclear | Not discussed in study | Yes | Rationale not provided | Partly | Some parameter uncertainty explored | Yes | No funding | No | Not a trial | Yes | Decision tree followed by a Markov model |
| Kowada 2015 [35] | Unclear | Not discussed in study | Yes | Rationale not provided | Partly | Only parameter uncertainty explored | Yes | No details about funding reported | No | Not a trial | Yes | Decision tree followed by a Markov model |
| Goodell [15] | Unclear | Not discussed in study | Yes | Rationale not provided | Partly | Only parameter uncertainty explored | Yes | Funding has been disclosed | No | Not a trial | Yes | Full details of model pathways not provided |
| Laskin [38] | Unclear | Not discussed in study | Yes | Rationale not provided | Yes | Rationale not provided | Yes | Declaration of no financial interests made | No | Not a trial | Yes | Decision tree followed by a Markov model |
| Kowada 2015 [25] | Unclear | Not discussed in study | Yes | Rationale not provided | Partly | Only parameter uncertainty explored | Yes | Funding has been disclosed | No | Not a trial | Yes | Decision tree followed by a Markov model |
| Kowada 2016 [44] | Unclear | Not discussed in study | Yes | Rationale not provided | Yes | Limited SA results reported | Yes | Funding not discussed | No | Not a trial | Yes | Decision tree followed by a Markov model |
| Kowada 2016 [33] | Unclear | Not discussed in study | Yes | Rationale not provided | Partly | Only parameter uncertainty explored | Yes | Declaration of no financial interests made | No | Not a trial | Yes | Decision tree followed by a Markov model |
| Kowada 2012 [46] | Unclear | Not discussed in study | Yes | Rationale not provided | Partly | Only parameter uncertainty explored | Yes | Declaration of no financial interests made | No | Not a trial | Yes | Decision tree followed by a Markov model |
| Kowada 2019 [37] | Unclear | Not discussed in study | Yes | Rationale not provided | Partly | Only parameter uncertainty explored | Yes | Declaration of no financial interests made | No | Not a trial | Yes | Decision tree followed by a Markov model |
| Kowada 2011 [49] | Unclear | Not discussed in study | Yes | Rationale not provided | Partly | Only parameter uncertainty explored | Yes | Declared no conflict of interests | No | Not a trial | Yes | Decision tree followed by a Markov model |
| Kowada 2013 [36] | Unclear | Not discussed in study | Yes | Rationale not provided | Partly | Only parameter uncertainty explored | Yes | Declared no conflict of interests | No | Not a trial | Yes | Decision tree followed by a Markov model |
| Kowada 2013 [32] | Unclear | Not discussed in study | Yes | Rationale not provided | Partly | Only parameter uncertainty explored | Yes | Declared no conflict of interests | No | Not a trial | Yes | Decision tree followed by a Markov model |
| Kowada 2014 [22] | Unclear | Not discussed in study | Yes | Rationale not provided | Partly | Only parameter uncertainty explored | Yes | Declared no conflict of interests | No | Not a trial | Yes | Decision tree followed by a Markov model |
| Kowada 2016 [21] | Unclear | Not discussed in study | No | Discount rate provided but rational for rate not given | Partly | Only parameter uncertainty explored | Yes | The author declared no funding had been provided for the study | NR | Not a trial | Yes | Simple decision tree followed by Markov with pathways in line with LTBI |
| Linas 2011 [11] | Unclear | Not discussed in study | No | Discount rate provided but rational for rate not given | Partly | Only parameter uncertainty explored | Yes | Funded by a national research body | NR | Not a trial | Yes | Decision tree followed by a Markov model |
| Capocci 2016 [173] | Unclear | Not discussed in study | Unclear | Not discussed in study | Partly | Only parameter uncertainty explored | No | Source of funding not provided | Unclear | Not clear if this was a trial | Unclear | Detail of model not provided |
| Jo 2020 [174] | Unclear | Not discussed in study | No | Discount rate provided but rational for rate not given | Partly | Only parameter uncertainty explored | Yes | Funded by CDC | NR | Not a trial | Unclear | Detail of model not provided |
| Abubakar 2018 [20] | Unclear | Not discussed in study | No | Discount rate provided but rational for rate not given | Partly | Only parameter uncertainty explored | Yes | Funded by NIHR | Yes | Trial registered | Yes | Decision tree + DES |
| Capocci 2020 [31] | Unclear | Not discussed in study | No | Discount rate provided but rational for rate not given | Partly | Only parameter uncertainty explored | Yes | The author declared no funding had been provided for the study | Yes | Trial registered | Yes | Simple decision tree with payoffs |
| Capocci (2016) (a) [175] | Unclear | Not discussed in study | Unclear | Not discussed in study | Partly | Only parameter uncertainty explored | No | Source of funding not provided | Unclear | Not clear if this was a trial | Unclear | Detail of model not provided |
| Capocci 2015 [28] | Unclear | Not discussed in study | No | Discount rate provided but rational for rate not given | Partly | Only parameter uncertainty explored | No | Source of funding not provided | NR | Not a trial | Yes | Simple decision tree with payoffs |
| Capocci 2014 [27] | Unclear | Not discussed in study | No | No discount rate included | No | Sensitivity analysis not undertaken | No | Source of funding not provided | Unclear | Not clear if this was a trial | Yes | Simple decision tree with payoffs |
| Capocci 2012 [26] | Unclear | Not discussed in study | No | No discount rate included | No | Sensitivity analysis not undertaken | No | Source of funding not provided | Unclear | Not clear if this was a trial | Yes | Simple decision tree with payoffs |
| Li 2018 [176] | Unclear | Not discussed in study | No | Discount rate provided but rational for rate not given | Partly | Not all key parameters - such as sensitivity of IGRA - were varied in SA | Yes | Funded by a national research body | NR | Not a trial | Yes | Decision tree followed by a Markov model |
| van der Have 2014 [39] | Unclear | Not discussed in study | No | Discount rate provided but rational for rate not given | Partly | Only parameter uncertainty explored | Yes | Funded by Abbott | NR | Not a trial | Yes | Markov model |

Part 3

| **Study** | **13. No treatment comparator bias: Is there an adequate comparator, i.e. care as usual?** | | **14. Wrong model bias: Is the model chosen adequate regarding the decision problem?** | | **15. Limited time horizon bias: Was a lifetime horizon chosen? Were shorter time horizons adequately justified?** | | **16. Bias related to data: Are the methods of data identification transparent? Are all choices justified adequately? Do the input parameters come from high-quality and well-designed studies?** | | **17. Bias related to baseline data: Are probabilities, for example, based in natural history data? Is transformation of rates into transition probabilities done accurately?** | | **18. Bias related to treatment effects: Are relative treatment effects synthesized using appropriate meta-analytic techniques? Are extrapolations documented and well justified? Are alternative assumptions explored regarding extrapolation?** | |
| --- | --- | --- | --- | --- | --- | --- | --- | --- | --- | --- | --- | --- |
|  | ***Relevant to study? Yes/No/Partly/Unclear*** | ***Comment - provide brief justification for judgement*** | ***Relevant to study? Yes/No/Partly/Unclear*** | ***Comment - provide brief justification for judgement*** | ***Relevant to study? Yes/No/Partly/Unclear*** | ***Comment - provide brief justification for judgement*** | ***Relevant to study? Yes/No/Partly/Unclear*** | ***Comment - provide brief justification for judgement*** | ***Relevant to study? Yes/No/Partly/Unclear*** | ***Comment - provide brief justification for judgement*** | ***Relevant to study? Yes/No/Partly/Unclear*** | ***Comment - provide brief justification for judgement*** |
| Al Abri 2020 [24] | Yes | Range of relevant comparators considered | Yes | The decision tree is appropriate to incorporate test outcomes and the markov model for long term disease outcomes | Yes | Lifetime horizon | Partly | Whilst the source of most values is published literature, the selection method is not described. The source of some variables is Oman data (no further details provided) | Unclear | Probabilities extracted from the literature. Selection method not reported | No | Efficacy of tests taken from literature but no systematic review was undertaken to identify and choose best evidence sources |
| Campbell 2017 [16] | Yes | Range of relevant comparators considered | Yes | A DES is appropriate | Yes | 10 years. Dominant strategy became more cost effective as time horizon increased | Partly | Whilst all values are referenced it is unclear the basis on which data was identified and chosen | Unclear | Probabilities extracted from the literature. Selection method not reported | No | Efficacy of tests taken from literature but no systematic review was undertaken to identify and choose best evidence sources |
| Campbell 2019 [17] | Yes | Range of relevant comparators considered | Yes | A DES is appropriate | Yes | 25 years | Partly | Whilst all values are referenced it is unclear the basis on which data was identified and chosen | Unclear | Probabilities extracted from the literature. Selection method not reported | No | Efficacy of tests taken from literature but no systematic review was undertaken to identify and choose best evidence sources |
| Campbell 2019 [18] | Yes | Range of relevant comparators considered | Yes | A DES is appropriate | Yes | 5 years | Partly | Whilst all values are referenced it is unclear the basis on which data was identified and chosen | Unclear | Probabilities extracted from the literature. Selection method not reported | No | Efficacy of tests taken from literature but no systematic review was undertaken to identify and choose best evidence sources |
| Tasillo 2017 [13] | Yes | Range of relevant comparators considered | Yes | Model is appropriate | Yes | lifetime horizon | Partly | Whilst all values are referenced it is unclear the basis on which data was identified and chosen | Unclear | Probabilities extracted from the literature. Selection method not reported | No | Efficacy of tests taken from literature but no systematic review was undertaken to identify and choose best evidence sources |
| Auguste 2016 [2] | Yes | Range of relevant comparators considered | Yes | Model is appropriate | Yes | DES run for 100 years | Yes | Systematic review used to inform the values of most inputs, some assumptions | Yes | Based on systematic review | Yes | Systematic review undertaken |
| Eralp 2012 [40] | Yes | Current and new treatment considered | Yes | Model is appropriate | Yes | Markov model run for 20 years | Partly | Whilst all values are referenced it is unclear the basis on which data was identified and chosen | Unclear | Probabilities extracted from the literature. Selection method not reported | No | Efficacy of tests taken from literature but no systematic review was undertaken to identify and choose best evidence sources |
| Png [42] | Yes | Comparators were strategies rather than interventions | Yes | Decision tree - only short-term costs and consequences considered | Yes | Medium and long-term costs and consequences not explored | Partly | Inputs referenced but SR not reported and unpublished hospital data used to populate some model parameters | Unclear | Probabilities extracted from the literature. Selection method not reported | Yes | Test efficacy assumed |
| Hayama 2017 [34] | Yes | Range of relevant comparators considered | Yes | Markov mode | Yes | lifetime horizon | No | Source of inputs NR | Unclear | Probabilities NR | Unclear | NR |
| Kowada 2011 [41] | Yes | Comparator appears appropriate | Yes | Model is appropriate | Yes | 20 years | No | Source of inputs NR | Unclear | Probabilities NR | Unclear | NR |
| Kowada 2015 [35] | Yes | Comparator appears appropriate | Yes | Model is appropriate | Yes | 50 years | Partly | Whilst all values are referenced, multiple sources have been used | Unclear | Probabilities extracted from the literature. Selection method not reported | No | Efficacy of tests taken from literature but no systematic review was undertaken to identify and choose best evidence sources |
| Goodell [15] | Yes | Comparator appears appropriate | Unclear | Full details of model structure not available | Yes | 48 years | Partly | Whilst all values are referenced it is unclear the basis on which data was identified and chosen | Unclear | Probabilities extracted from the literature. Selection method not reported | No | Efficacy of tests taken from literature but no systematic review was undertaken to identify and choose best evidence sources |
| Laskin [38] | Yes | Comparator appears appropriate, although IGRA not recommended for children - results including IGRA stated as exploratory |  |  | Yes | Lifetime (100 years) | Partly | Whilst all values are referenced it is unclear the basis on which data was identified and chosen | Unclear | Probabilities extracted from the literature. Selection method not reported | No | Efficacy of tests taken from literature but no systematic review was undertaken to identify and choose best evidence sources |
| Kowada 2015 [25] | Yes | Comparator appears appropriate | Yes | Model is appropriate | Yes | Up to age 60 | Partly | Whilst all values are referenced it is unclear the basis on which data was identified and chosen | Unclear | Probabilities extracted from the literature. Selection method not reported | No | Efficacy of tests taken from literature but no systematic review was undertaken to identify and choose best evidence sources |
| Kowada 2016 [44] | Yes | Comparator appears appropriate | Yes | Model is appropriate | Yes | Lifetime | Partly | Source of inputs NR | Unclear | Probabilities NR | Unclear | NR |
| Kowada 2016 [33] | Yes | Comparator appears appropriate | Yes | Model is appropriate | Yes | Lifetime but length not provided | Partly | Whilst all values are referenced it is unclear the basis on which data was identified and chosen | Unclear | Probabilities extracted from the literature. Selection method not reported | No | Efficacy of tests taken from literature but no systematic review was undertaken to identify and choose best evidence sources |
| Kowada 2012 [46] | Yes | Comparator appears appropriate | Yes | Model is appropriate | Yes | Up to 80 years of age | Partly | Whilst all values are referenced it is unclear the basis on which data was identified and chosen | Unclear | Probabilities extracted from the literature. Selection method not reported | No | Efficacy of tests taken from literature but no systematic review was undertaken to identify and choose best evidence sources |
| Kowada 2019 [37] | Yes | Comparator appears appropriate | Yes | Model is appropriate | Yes | Reported as lifetime but length not provided | Partly | Whilst all values are referenced it is unclear the basis on which data was identified and chosen | Unclear | Probabilities extracted from the literature. Selection method not reported | No | Efficacy of tests taken from literature but no systematic review was undertaken to identify and choose best evidence sources |
| Kowada 2011 [49] | Yes | Comparator appears appropriate | Yes | Model is appropriate | Yes | Reported as lifetime but length not provided | Partly | Whilst all values are referenced it is unclear the basis on which data was identified and chosen | Unclear | Probabilities extracted from the literature. Selection method not reported | No | Efficacy of tests taken from literature but no systematic review was undertaken to identify and choose best evidence sources |
| Kowada 2013 [36] | Yes | Comparator appears appropriate | Yes | Model is appropriate | Yes | Reported as lifetime but length not provided | Partly | Whilst all values are referenced it is unclear the basis on which data was identified and chosen | Unclear | Probabilities extracted from the literature. Selection method not reported | No | Efficacy of tests taken from literature but no systematic review was undertaken to identify and choose best evidence sources |
| Kowada 2013 [32] | Yes | Comparator appears appropriate | Yes | Model is appropriate | Yes | Reported as lifetime but length not provided | Partly | Whilst all values are referenced it is unclear the basis on which data was identified and chosen | Unclear | Probabilities extracted from the literature. Selection method not reported | No | Efficacy of tests taken from literature but no systematic review was undertaken to identify and choose best evidence sources |
| Kowada 2014 [22] | Yes | Comparator appears appropriate | Yes | Model is appropriate | Yes | 30 years | Partly | Whilst all values are referenced it is unclear the basis on which data was identified and chosen | Unclear | Probabilities extracted from the literature. Selection method not reported | No | Efficacy of tests taken from literature but no systematic review was undertaken to identify and choose best evidence sources |
| Kowada 2016 [21] | Yes | Range of relevant comparators considered | Yes | The decision tree is appropriate to incorporate test outcomes and the markov model for long term disease outcomes | Yes | Lifetime horizon | Partly | Whilst all values are referenced it is unclear the basis on which data was identified and chosen | Not relevant | No baseline data are presented | No | Efficacy of tests taken from literature but no systematic review was undertaken to identify and choose best evidence sources |
| Linas 2011 [11] | Yes | Range of relevant comparators considered | Yes | The decision tree is appropriate to incorporate test outcomes and the markov model for long term disease outcomes | Yes | Lifetime horizon | Partly | Whilst the source of most values is published literature, the selection method is not described. The source of costs is poorly reported | Unclear | Probabilities extracted from the literature. Selection method not reported | No | Efficacy of tests taken from literature but no systematic review was undertaken to identify and choose best evidence sources |
| Capocci 2016 [173] | Yes | Range of relevant comparators considered | Unclear | Detail of model not provided | Unclear | Time horizon not provided | No | Detail of sources not provided | Unclear | No baseline data are presented | No | Unclear where efficacy was derived |
| Jo 2020 [174] | No | Other testing options not considered | Unclear | Detail of model not provided | No | 30 year horizon not justified | Partly | Whilst the source of most values is published literature, the selection method is not described. The source of costs is poorly reported | Unclear | Probabilities extracted from the literature. Selection method not reported | No | Efficacy of tests taken from literature but no systematic review was undertaken to identify and choose best evidence sources |
| Abubakar 2018 [20] | Yes | Range of relevant comparators considered | Yes | DES is appropriate | Yes | Lifetime horizon | Partly | Whilst the source of most values is published literature, the selection method is not described. The source of costs is poorly reported | Yes | Probabilities taken from trial | NR | Taken from trial |
| Capocci 2020 [31] | Yes | Range of relevant comparators considered | No | Cannot account for long term changes in TB activation | Yes | Lifetime horizon | Partly | Whilst the source of most values is published literature, the selection method is not described. The source of costs is poorly reported | Yes | Probabilities taken from trial | NR | Taken from one data source |
| Capocci (2016) (a) [175] | Yes | Range of relevant comparators considered | Unclear | Detail of model not provided | Unclear | Time horizon not provided | No | Detail of sources not provided | Unclear | No baseline data are presented | No | Source(s) of efficacy data not reported |
| Capocci 2015 [28] | Yes | Range of relevant comparators considered | No | Cannot account for long term changes in TB activation | Yes | Lifetime horizon | Partly | Whilst the source of most values is published literature, the selection method is not described. The source of costs is poorly reported | Yes | Probabilities from database | NR | Taken from a database analysis |
| Capocci 2014 [27] | Yes | Range of relevant comparators considered | No | Cannot account for long term changes in TB activation | Yes | Lifetime horizon | Partly | Whilst the source of most values is published literature, the selection method is not described. The source of costs is poorly reported | Yes | Probabilities taken from trial | NR | Taken from one data source |
| Capocci 2012 [26] | Yes | Range of relevant comparators considered | No | Cannot account for long term changes in TB activation | Unclear | Time horizon not provided | Partly | Whilst the source of most values is published literature, the selection method is not described. The source of costs is poorly reported | Yes | Probabilities from database | NR | Taken from one data source |
| Li 2018 [176] | Yes | Range of relevant comparators considered | Yes | The decision tree is appropriate to incorporate test outcomes and the Markov model for long term disease outcomes | Partly | 20 year time horizon which is probably sufficient for patients aged 65 | Partly | Whilst the source of most values is published literature, the selection method is not described. | Unclear | Probabilities extracted from the literature. Selection method not reported | No | Efficacy of tests taken from literature but no systematic review was undertaken to identify and choose best evidence sources |
| van der Have 2014 [39] | Yes | Range of relevant comparators considered | No | Cannot account for long term changes in TB activation | No | Only five year time horizon | Partly | Whilst the source of most values is published literature, the selection method is not described. | Unclear | Probabilities extracted from the literature. Selection method not reported | No | Efficacy of tests taken from literature but no systematic review was undertaken to identify and choose best evidence sources |
